# Stillbirth risk by fetal size among 126.5 million births in 15 countries from 2000 to 2020: A fetuses‐at‐risk approach

**DOI:** 10.1111/1471-0528.17890

**Published:** 2024-07-11

**Authors:** Yemisrach B. Okwaraji, Lorena Suárez‐Idueta, Eric O. Ohuma, Ellen Bradley, Judith Yargawa, Veronica Pingray, Gabriela Cormick, Adrienne Gordon, Vicki Flenady, Erzsébet Horváth‐Puhó, Henrik Toft Sørensen, Liili Abuladze, Mohammed Heidarzadeh, Narjes Khalili, Khalid A. Yunis, Ayah Al Bizri, Arturo Barranco, Aimée E. van Dijk, Lisa Broeders, Fawzya Alyafei, Tawa O. Olukade, Neda Razaz, Jonas Söderling, Lucy K. Smith, Ruth J. Matthews, Rachael Wood, Kirsten Monteath, Isabel Pereyra, Gabriella Pravia, Sarka Lisonkova, Qi Wen, Joy E. Lawn, Hannah Blencowe, the National Vulnerable Newborn Mortality Collaborative Group and 15 Vulnerable Newborn Measurement Core Group, Veronica Pingray, Gabriela Cormick, José Belizan, Luz Gibbons, Carlos Guevel, Vicki Flenady, Adrienne Gordon, Kara Warrilow, Harriet Lawford, Jeremy Oats, Michael Humphrey, Erzsébet Horváth‐Puhó, Henrik T. Sørensen, Luule Sakkeus, Liili Abuladze, Khalid A. Yunis, Ayah Al Bizri, Pascale Nakad, Shamala Karalasingam, J Ravichandran, R Jeganathan, Nurakman Binti Baharum, Lorena Suárez‐Idueta, Arturo Barranco Flores, Jesus Felipe Gonzalez Roldan, Sonia Lopez Alvarez, Lisa Broeders, Fawziya Alyafei, Mai AlQubaisi, Tawa O. Olukade, Hamdy A. Ali, Mohamad Rami Alturk, Neda Razaz, Jonas Söderling, Lucy K Smith, Bradley N. Manktelow, Ruth J. Matthews, Elizabeth Draper, Alan Fenton, Jennifer J. Kurinczuk, Rachael Wood, Celina Davis, Kirsten Monteath, Samantha Clarke, Isabel Pereyra, Gabriella Pravia, Sarka Lisonkova, Qi Wen, Joy E. Lawn, Hannah Blencowe, Eric Ohuma, Yemisrach B Okwaraji, Judith Yargawa, Ellen Bradley, Bob Black, Joanne Katz, Dan Erchick, Elizabeth Hazel, Mike Diaz, Anne CC Lee

**Affiliations:** ^1^ Maternal, Adolescent, Reproductive and Child Health (MARCH) Centre London School of Hygiene & Tropical Medicine London UK; ^2^ Mexican Society of Public Health Mexico City Mexico; ^3^ Institute for Clinical Effectiveness and Health Policy Ciudad Autónoma de Buenos Aires Buenos Aires Argentina; ^4^ Universidad Nacional de la Matanza San Justo, Buenos Aires Argentina; ^5^ Faculty of Medicine and Health University of Sydney Sydney New South Wales Australia; ^6^ Centre of Research Excellence in Stillbirth, Mater Research Institute The University of Queensland (MRI‐UQ) Brisbane Queensland Australia; ^7^ Department of Clinical Epidemiology Aarhus University and Aarhus University Hospital Aarhus Denmark; ^8^ Estonian Institute for Population Studies, School of Governance, Law and Society Tallinn University Tallinn Estonia; ^9^ Paediatrics Department Alzahra Hospital Iran Tabriz Iran; ^10^ Preventive Medicine and Public Health Research Centre, School of Medicine Iran University of Medical Sciences Tehran Iran; ^11^ The National Collaborative Perinatal Neonatal Network (NCPNN) Coordinating Center at the Department of Paediatrics and Adolescent Medicine American University of Beirut Beirut Lebanon; ^12^ Directorate of Health Information Ministry of Health Mexico City Mexico; ^13^ Perined Utrecht the Netherlands; ^14^ Hamad General Hospital Doha Qatar; ^15^ Clinical Epidemiology Division, Department of Medicine Solna Karolinska Institute Stockholm Sweden; ^16^ Department of Population Health Sciences, College of Life Sciences University of Leicester Leicester UK; ^17^ Public Health Scotland Edinburgh UK; ^18^ Usher Institute University of Edinburgh Edinburgh UK; ^19^ Pregnancy, Birth and Child Health Team Public Health Scotland Edinburgh UK; ^20^ Faculty of Health Sciences Catholic University of Maule Talca Chile; ^21^ Catholic University of Uruguay Montevideo Uruguay; ^22^ Department of Obstetrics & Gynaecology University of British Columbia Vancouver Canada

**Keywords:** fetuses‐at‐risk approach, gestational age, preterm birth, size for gestational age, stillbirth

## Abstract

**Objective:**

To compare stillbirth rates and risks for small for gestational age (SGA), large for gestational age (LGA) and appropriate for gestational age (AGA) pregnancies at 24–44 completed weeks of gestation using a birth‐based and fetuses‐at‐risk approachs.

**Design:**

Population‐based, multi‐country study.

**Setting:**

National data systems in 15 high‐ and middle‐income countries.

**Population:**

Live births and stillbirths.

**Methods:**

A total of 151 country‐years of data, including 126 543 070 births across 15 countries from 2000 to 2020, were compiled. Births were categorised into SGA, AGA and LGA using INTERGROWTH‐21st standards. Gestation‐specific stillbirth rates, with total births as the denominator, and gestation‐specific stillbirth risks, with fetuses still in utero as the denominator, were calculated from 24 to 44 weeks of gestation.

**Main Outcome Measures:**

Gestation‐specific stillbirth rates and risks according to size at birth.

**Results:**

The overall stillbirth rate was 4.22 per 1000 total births (95% CI 4.22–4.23) across all gestations. Applying the birth‐based approach, the stillbirth rates were highest at 24 weeks of gestation, with 621.6 per 1000 total births (95% CI 620.9–622.2) for SGA pregnancies, 298.4 per 1000 total births (95% CI 298.1–298.7) for AGA pregnancies and 338.5 per 1000 total births (95% CI 337.9–339.0) for LGA pregnancies. Applying the fetuses‐at‐risk approach, the gestation‐specific stillbirth risk was highest for SGA pregnancies (1.3–1.4 per 1000 fetuses at risk) prior to 29 weeks of gestation. The risk remained stable between 30 and 34 weeks of gestation, and then increased gradually from 35 weeks of gestation to the highest rate of 8.4 per 1000 fetuses at risk (95% CI 8.3–8.4) at ≥42 weeks of gestation. The stillbirth risk ratio (RR) was consistently high for SGA compared with AGA pregnancies, with the highest RR observed at ≥42 weeks of gestation (RR 9.2, 95% CI 15.2–13.2), and with the lowest RR observed at 24 weeks of gestation (RR 3.1, 95% CI 1.9–4.3). The stillbirth RR was also consistently high for SGA compared with AGA pregnancies across all countries, with national variability ranging from RR 0.70 (95% CI 0.43–0.97) in Mexico to RR 8.6 (95% CI 8.1–9.1) in Uruguay. No increased risk for LGA pregnancies was observed.

**Conclusions:**

Small for gestational age (SGA) was strongly associated with stillbirth risk in this study based on high‐quality data from high‐ and middle‐income countries. The highest RRs were seen in preterm gestations, with two‐thirds of the stillbirths born as preterm births. To advance our understanding of stillbirth, further analyses should be conducted using high‐quality data sets from low‐income settings, particularly those with relatively high rates of SGA.

**Key findings**

**What was known?**

Size at birth reflects fetal growth in utero. Poor fetal growth can result from several underlying maternal, placental or fetal causes, all of which are associated with an increased risk of stillbirth. The risk of stillbirth is significantly influenced by fetal size. Small for gestational age (SGA) pregnancies have a higher risk than appropriate for gestational age (AGA) pregnancies.

**What was done that is new?**

This study compared the risk of stillbirth across different gestational weeks, starting from 24 weeks of gestation, in 15 countries, through a ‘birth‐based’ approach (with the total number of births as the denominator) and ‘fetuses‐at‐risk approach’ (with the number of pregnancies still in utero as the denominator). For each approach, stillbirth rates by week of gestation were calculated overall and by size for gestational age category (SGA, AGA and LGA), according to INTERGROWTH‐21st standards.

**What was found?**

Overall, 21.1% of stillbirths were SGA, 64.8% were AGA and 13.9% were LGA, although the distribution varied across the 15 included countries. Stillbirth rates peaked from 24 weeks of gestation, then progressively declined with advancing gestation. Throughout gestation, SGA pregnancies consistently had a higher risk of stillbirth compared with AGA pregnancies. LGA pregnancies also have an increased risk of stillbirth compared with AGA pregnancies, but this risk was not as high as that observed in SGA pregnancies. Although national variations in stillbirth risk were observed, the combined analysis across countries showed that SGA pregnancies had a consistently higher risk of stillbirth compared with AGA pregnancies. There was no evidence of an increased risk for LGA pregnancies compared with AGA pregnancies.

**What is next?**

The prospective risk of stillbirth during pregnancy varies according to fetal size, and the greatest risk occurs at 24 weeks of gestation. This elevated risk during early gestation underscores potential challenges in identifying fetuses at risk. Through the ‘fetuses‐at‐risk’ approach, wherein the gestational age is considered the survival time, the specific risk of stillbirth can be calculated for each gestational period. This approach offers valuable insights into the prevalence of stillbirth.

## 1 INTRODUCTION

Stillbirths, defined as the loss of a baby during pregnancy at or after 22^+0^ weeks of gestation, pose a major global burden on maternal and newborn health.^1^ Although most stillbirths are preventable, an estimated 1.9 million babies were stillborn globally after 28 weeks of gestation in 2021.^2^ Furthermore, progress in decreasing these numbers over the past two decades has been slow.^2^ Various risk factors contribute to this continuing heavy burden. In many cases the pathways to fetal death and stillbirth are similar to those observed in preterm birth (before 37 weeks of gestation) and fetal growth disorders, and both small for gestational age (SGA) and large for gestational age (LGA) fetuses are at elevated risk of stillbirth.^3^, ^4^ These risk factors underscore the complex interplay between gestational development and the occurrence of stillbirth. Therefore, a more granular approach to assessing newborn vulnerability has been called for to accelerate progress towards the UN Global Strategy for Women's, Children's and Adolescents Health and Every Newborn Action Plan targets.^5^

Recent studies have highlighted the significant effects of fetal size at birth on stillbirth and neonatal mortality risks, by using newborn types that combine categorised birthweight, categorised gestational age groups (preterm, <37 weeks of gestation vs term and ≥37 weeks of gestation) and categorised size for gestational age (SGA, with birthweight below the tenth centile for gestational age and sex; AGA, appropriate for gestational age, with birthweight between the tenth and 90th centiles; and LGA, with birthweight above the 90th centile).^6^, ^7^, ^8^ However, using a simple dichotomy between preterm and term births fails to capture the large variation in risk at different gestational ages along the preterm continuum through early term and post‐term.^9^, ^10^ The limited studies exploring this association have demonstrated that the risk of SGA stillbirth increases throughout pregnancy.^9^, ^10^ Beyond the vulnerability of small babies, LGA babies have also been shown to be at elevated risk of stillbirth from 41 weeks of gestation.^10^, ^11^

Here, we build on previous work on stillbirth risk among newborn types by considering both a birth‐based approach and the fetuses‐at‐risk approach when calculating gestational age‐specific rates of stillbirth.^12^ The birth‐based approach focuses on the gestational age‐specific outcomes of pregnancies ending in a given week, in this case assessing the proportion ending in stillbirth. This method, although informative regarding the proportion of stillbirth at various points in pregnancy, does not capture the complete risk profile during gestation and thus has limited value for assessing prospective risk in, for example, clinical decision‐making. In contrast, the fetuses‐at‐risk approach considers gestational age as a measure of survival time.^13^ Thus, rather than births, it uses surviving in utero fetuses (at risk of adverse outcomes) as the denominator for calculating gestational age‐specific stillbirth rates.^14^ By using this approach, researchers can estimate the risk of stillbirth in continuing pregnancies at any given gestational age and gain insights into the evolving dynamics of risk throughout pregnancy. This study used high‐quality, national administrative data sets collected over two decades to calculate gestational age‐specific stillbirth rates/risks by size‐for‐gestational age categories (SGA, AGA and LGA) using both birth‐based and fetuses‐at‐risk approaches from 24 to 44 weeks of gestation.

## 2 METHODS

### 2.1 Data source

**FIGURE 1:**
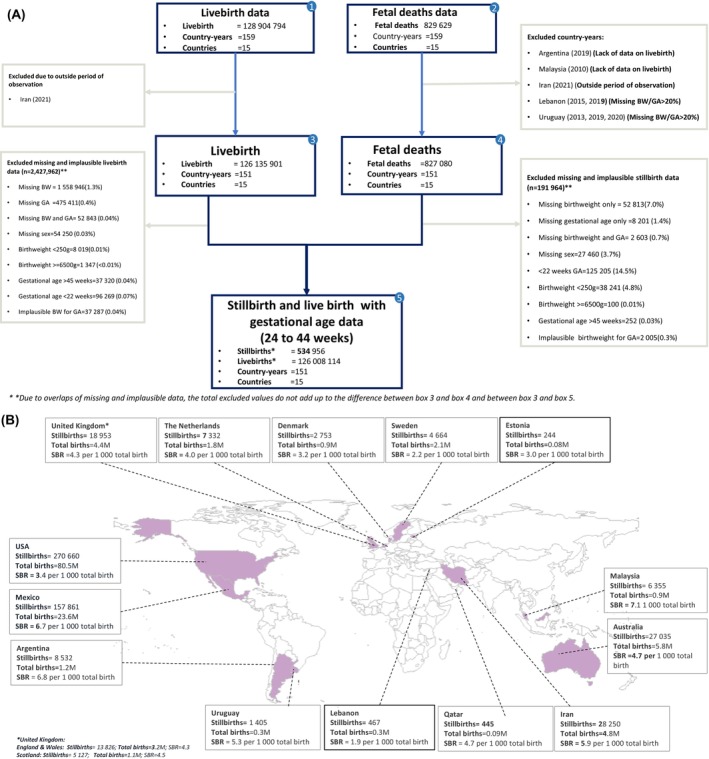
(A) Flow chart of stillbirth data inclusion and exclusion. (B) Number of stillbirths included by country (*n* = 534 956) for 24–44 weeks of gestation.

A detailed description of how data were collated has been published elsewhere.^6^, ^7^ In brief, stillbirth data from the years 2000–2020 were compiled from 15 countries, with a total of 151 country‐years. Country‐years with 20% or more missing data in any of the categories of birthweight, gestational age or sex were excluded (Figure 1A). The additional information on the RECORD statement and ethical approval can be found in the supporting materials (Tables S1 and S2). Individual birth records missing essential information, such as birthweight, gestational age or sex, for the assessment of size for gestational age were excluded from the data set. Furthermore, birth records falling outside the gestational age range of interest, of <24^+0^ or >44^+6^ weeks of gestation, as well as those with implausible combinations of birthweight and gestational age (defined as birthweight ±5 standard deviations from the mean birthweight for gestational age) were also excluded. To ensure data quality, an assessment of all included data sets was performed for each country‐year (Table S3).

### 2.2 Exposure definition

Each pregnancy was categorised based on gestational age and size for gestational age. We assessed SGA, LGA and AGA by comparing birthweights at each gestational age (in weeks) using a modified version of the INTERGROWTH‐21st international standards for newborn.^15^ We assessed SGA, LGA and AGA using a mid‐week standard when gestational age was recorded in completed weeks to reduce the potential risk of misclassification.^16^ Given the large variation in definitions and recording practices for stillbirth observed across countries, only births from 24^+0^ weeks of gestation were considered for analysis (Table S6). Each pregnancy was categorised as SGA, LGA or AGA, defined as follows^17^, ^18^, ^19^:

- AGA: fetus or infant whose birthweight falls between the 10th and the 90th percentiles on standard growth charts.
- SGA: fetus or infant whose birthweight falls below the 10th percentile on standard growth charts for their gestational age.
- LGA: fetus or infant whose birthweight falls above the 90th percentile on standard growth charts for their gestational age.

### 2.3 Measurements for association of stillbirth with gestational age and attained size

#### 2.3.1 Gestational age‐specific stillbirth rate (using a birth‐based approach with the total number of births in that week as the denominator)

The rate (proportion) was determined as the number of stillbirths reported in a specific gestational week divided by the total number of births that occurred during the same week expressed per 1000 total births. For example, for 24 weeks of gestation, the number of stillbirths between 24^+0^ and 24^+6^ weeks of gestation is divided by the total number of births (live births and stillbirths) between 24^+0^ and 24^+6^ week of gestation, multiplied by 1000. A meta‐analysis was conducted and the gestational age‐specific stillbirth rates with 95% CIs for each gestational week were presented in a forest plot. The 95% CI was calculated using the standard error of the rate estimate.

#### 2.3.2 Gestational age‐specific prospective risk of stillbirth (using a fetuses‐at‐risk approach, with fetuses still in utero at the beginning of each gestational week as the denominator)

This was calculated by dividing the number of stillbirths occurring at a specific gestational week by the total number of fetuses still in utero or delivered at the same gestational week up to 44 weeks of gestation. It is expressed as the risk of stillbirths per 1000 fetuses at risk. For example, the gestational age‐specific stillbirth risk at 24 weeks of gestation was calculated as the number of stillbirths between 24^+0^ and 24^+6^ week of gestation divided by the total number of fetuses in‐utero or delivered in that week of gestation, including all fetuses up to 44 weeks of gestation, multiplied by 1000. A meta‐analysis was conducted and the stillbirth risk with 95% CI for each gestational week was presented using a forest plot.

#### 2.3.3 Gestational age‐specific stillbirth risk ratio (using a fetuses‐at‐risk approach with fetuses still in utero as the denominator)

Gestational age‐specific stillbirth risk ratios comparing SGA pregnancies with AGA pregnancies and comparing LGA pregnancies with AGA pregnancies were calculated using a generalised linear mixed‐effect model. This allowed the estimation of risk ratio estimates from each gestational week. Summary statistics for the meta‐analysis model and the heterogeneity statistic (*I* ^2^) across gestational weeks were assessed. The forest plot displays the gestation‐specific risk ratio estimates, along with their corresponding 95% CIs. In addition, country‐specific risk ratios for SGA versus AGA and LGA versus AGA across all gestational age weeks were pooled. Similarly, country‐specific risk ratios were calculated by comparing SGA and LGA pregnancies with AGA pregnancies for each country. All analyses were conducted in R 4.3.2 (R Foundtion for Statistical Computing, Vienna, Austria).^20^

## 3 RESULTS

### 3.1 Data description

**FIGURE 2:**
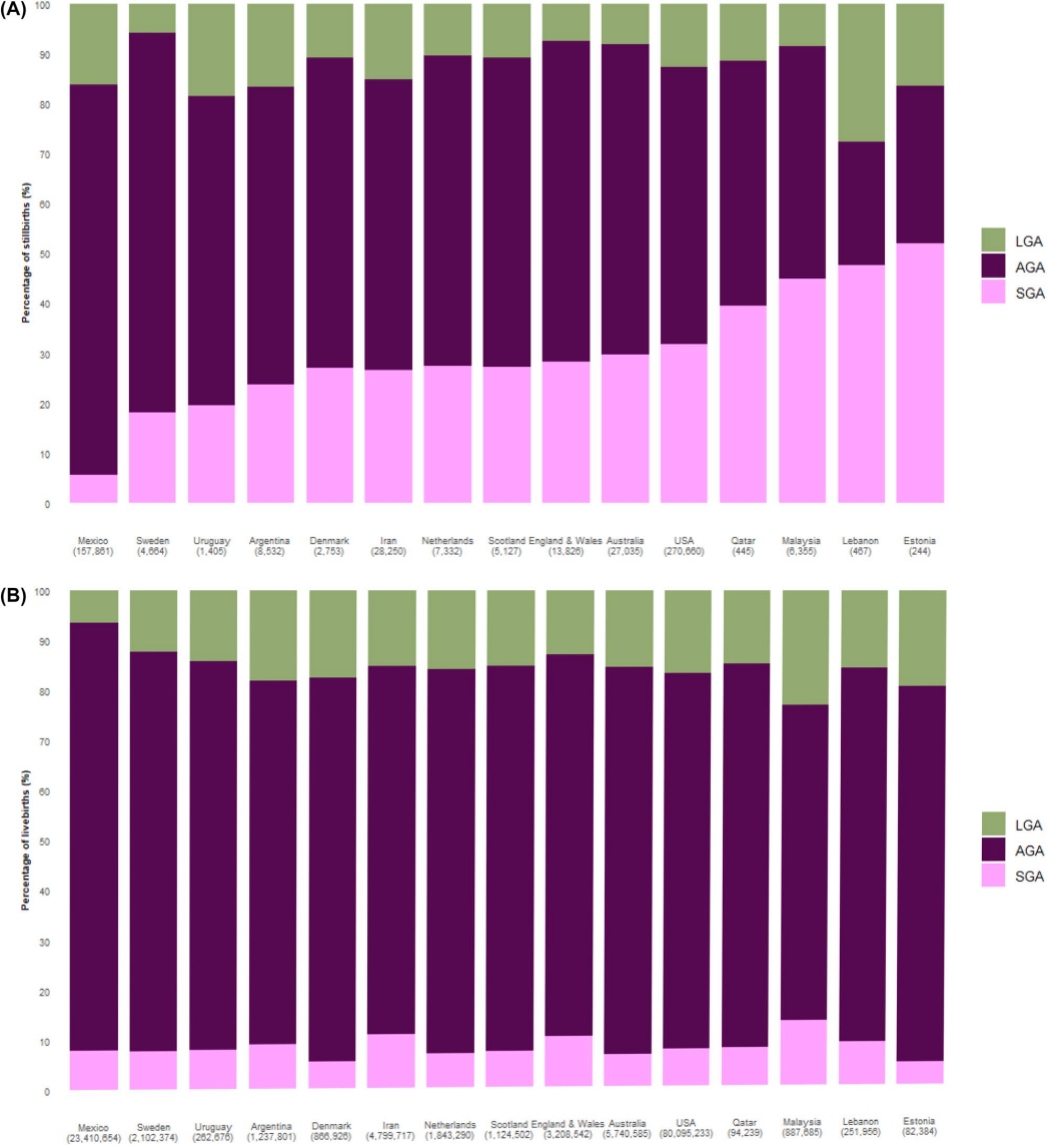
(A) Distribution of size for gestational age (for 24–44 weeks of gestation) among all stillbirths. (B) Distribution of size for gestational age (for 24–44 weeks of gestation) among all live births.

Data from a span of 151 country‐years, including a total of 126 543 070 births (comprising 126 008 114 live births and 534 956 stillbirths), were compiled from 15 countries for the period 2000–2020 for this analysis (Figure 1A,B). A relatively higher percentage of missing information on core variables was reported in fetal deaths recorded in Lebanon (with >20% missing birthweight in 2013, 2019 and 2020) and Uruguay (with >20% missing birthweight in 2019 and with >20% missing gestational age in 2018 and 2020). Consequently, these cases were excluded from the analysis (Table S3). The percentage of live births and stillbirths according to baseline maternal characteristics are presented in Table S4A,B. Stillbirths were more frequent amongst women with lower education levels, specifically those with primary education only in Latin American countries, notably Mexico (53% of stillbirths) and Argentina (43% of stillbirths). In contrast, stillbirths were less frequent amongst women with higher educational attainment in European countries. For example, in Denmark, 43% of stillbirths were recorded amongst women with upper secondary education, whereas in Sweden, the corresponding figure was 40% (Table S4B). The proportion of all stillbirths categorised as SGA at birth varied widely, ranging from 4.1% in Mexico to 52.5% in Malaysia, whereas the proportion categorised as LGA ranged from 7.9% in England and Wales to 17.6% in Mexico (Figure 2A). In contrast, most live births (77%) were born AGA, whereas 17.7% were born LGA, and the remaining 5.3% were born SGA (Figure 2B).

The prevalence of SGA, AGA and LGA pregnancies was analysed across gestational weeks (Table 1). At 24 weeks of gestation, the percentages of SGA, AGA and LGA pregnancies were 26.4% (95% CI 24.7%–28.0%), 61.3% (95% CI 60.6%–62.0%) and 12.7% (95% CI 10.8%–14.6%), respectively. The prevalence varied across gestational ages, with fluctuations observed in the percentages of SGA, AGA and LGA pregnancies. For instance, at 39 weeks of gestation, the prevalence of SGA pregnancies decreased to 34.7% (95% CI 33.7%–35.8%), whereas the prevalence of AGA pregnancies increased to 56.2% (95% CI 55.6%–56.9%) and the prevalence of LGA pregnancies increased to 11.3% (95% CI 9.5%–13.0%). Although there were fluctuations in prevalence over gestational weeks, no consistent decreasing or increasing trend was observed for SGA, AGA and LGA pregnancies (Table 1).

**TABLE 1:** Prevalence of small for gestational age (SGA), appropriate for gestational age (AGA) and large for gestational age (LGA) by gestational week (24–44 weeks of gestation) in 15 countries, 2000–2020.

Gestational week	Fetuses at risk	Total births	Live births	Stillbirths	Prevalence of SGA, AGA and LGA		
					% of SGA (95% CI)	% of AGA (95% CI)	% of LGA (95% CI)
24	126 543 070	142 813	100 553	42 260	26.4 (24.7–28.0)	61.3 (60.6–62.0)	12.7 (10.8–14.6)
25	126 400 257	141 263	110 063	31 200	34.7 (33.7–35.8)	56.2 (55.6–56.9)	11.3 (9.5–13.0)
26	126 258 994	157 295	129 195	28 100	37.9 (37.1–38.8)	56.6 (56.1–57.1)	12.0 (10.5–13.6)
27	126 101 699	172 800	148 583	24 217	40.0 (39.2–40.7)	53.6 (53–54.2)	12.4 (11.2–13.7)
28	125 928 899	217 298	189 435	27 863	41.8 (41.1–42.6)	53.4 (52.8–54.0)	12.1 (10.8–13.4)
29	125 711 601	227 595	206 826	20 769	39.3 (38.6–40.1)	55.8 (55.1–56.5)	12.2 (11–13.4)
30	125 484 006	313 859	289 044	24 815	29.6 (28.2–31.0)	59.9 (59.2–60.5)	12.6 (11.5–13.8)
31	125 170 147	374 273	352 566	21 707	30.5 (29.5–31.5)	61.2 (60.6–61.7)	10.0 (7.8–12.3)
32	124 795 874	587 744	561 021	26 723	24.0 (22.3–25.6)	64.7 (64–65.3)	9.9 (8.1–11.7)
33	124 208 130	806 845	782 300	24 545	28.0 (27.0–28.9)	69.9 (69.4–70.4)	10.0 (8.0–12.0)
34	123 401 285	1 443 752	1 415 460	28 292	28.6 (27.6–29.7)	67.7 (67.2–68.2)	11.3 (9.8–12.8)
35	121 957 533	2 283 714	2 253 979	29 735	31.8 (30.9–32.8)	64.9 (64.4–65.5)	11.8 (10.5–13.1)
36	119 673 819	4 673 270	4 637 071	36 199	30.9 (30.1–31.7)	62.6 (62.1–63.0)	13.0 (11.5–14.4)
37	115 000 549	10 727 308	10 689 577	37 731	24.2 (22.7–25.7)	60.0 (59.5–60.5)	14.7 (13.5–15.9)
38	104 273 241	24 150 363	24 108 155	42 208	26.4 (24.7–28.0)	61.3 (60.6–62.0)	12.7 (10.8–14.6)
39	80 122 878	38 793 915	38 757 919	35 996	34.7 (33.7–35.8)	56.2 (55.6–56.9)	11.3 (9.5–13.0)
40	41 328 963	30 450 243	30 418 412	31 831	37.9 (37.1–38.8)	56.6 (56.1–57.1)	12.0 (10.5–13.6)
41	10 878 720	9 549 633	9 537 317	12 316	40.0 (39.2–40.7)	53.6 (53.0–54.2)	12.4 (11.2–13.7)
≥42	1 329 087	1 329 087	1 320 638	8449	41.8 (41.1–42.6)	53.4 (52.8–54.0)	12.1 (10.8–13.4)

### 3.2 Stillbirth rates (using a birth‐based approach)

**FIGURE 3:**
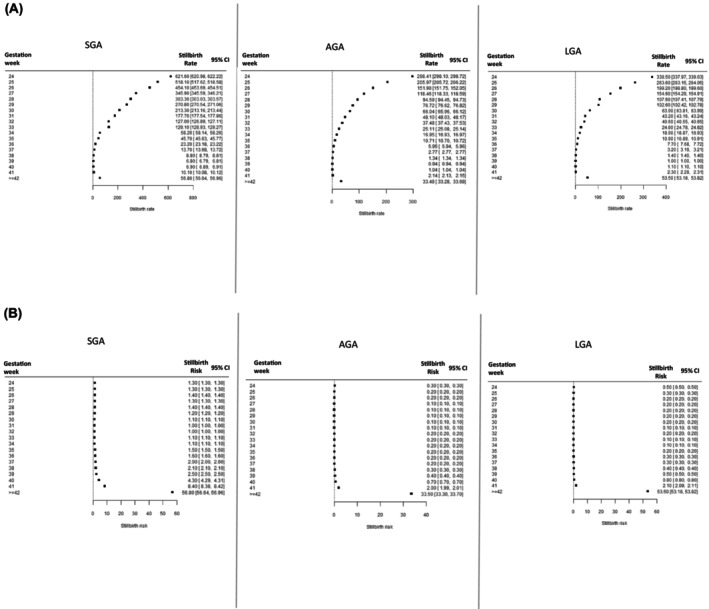
(A) Stillbirth rate for small for gestational age (SGA), appropriate for gestational age (AGA) and large for gestational age (LGA) pregnancies by gestational week using a birth‐based approach across 15 countries, 2000–2020. (B) Stillbirth risk for SGA, AGA and LGA pregnancies by gestational week using a fetuses‐at‐risk approach across 15 countries, 2000–2020.

The overall stillbirth rate was 4.22 per 1000 total births (95% CI 4.22–4.23) across all gestations. The gestational age‐specific stillbirth rates were highest at 24 weeks of gestation, with 621.6 stillbirths per 1000 total births (95% CI 620.9–622.2) for SGA pregnancies, 298.4 per 1000 total births (95% CI 298.1.5–298.7) for AGA pregnancies and 338.5 per 1000 total births (95% CI 337.9–339.0) for LGA pregnancies (Figure 3A). As pregnancy progressed, the rates gradually decreased at each gestational age, reaching the lowest rates at 39 weeks of gestation, with 6.8 stillbirths per 1000 total births (95% CI 6.8–6.9) for SGA pregnancies, 0.9 stillbirths per 1000 total births (95% CI 0.9–0.9) for AGA pregnancies and 1.0 stillbirths per 1000 total births (95% CI 1.0–1.0) for LGA pregnancies, and then increased slightly up to ≥42 weeks of gestation, with 56.8 stillbirths per 1000 total births (95% CI 56.6–56.9) for SGA pregnancies, 33.5 stillbirths per 1000 total births (95% CI 33.3–33.7) for AGA pregnancies and 53.5 stillbirths per 1000 total births (95% CI 53.2–53.8) for LGA pregnancies (Figure 3A). Notably, the stillbirth rates were consistently higher among SGA pregnancies than among AGA and LGA pregnancies throughout the gestational weeks (Figure 3A).

### 3.3 Stillbirth risk (using a fetuses‐at‐risk approach)

The gestational age‐specific stillbirth risk was highest for SGA pregnancies (1.3–1.4 per 1000 fetuses at risk) prior to 29 weeks of gestation. The risk remained stable between 30 and 34 weeks of gestation, and then increased gradually from 35 weeks of gestation, with the highest rate of 8.4 per 1000 fetuses at risk (95% CI 8.3–8.4) seen at ≥42 weeks of gestation. A similar pattern was observed for AGA and LGA pregnancies, with the highest stillbirth risk occurring at ≥42 weeks of gestation: 33.5 per 1000 fetuses at risk (95% CI 33.3–33.7) for AGA pregnancies and 53.5 per 1000 fetuses at risk (95% CI 53.2–53.8) for LGA pregnancies (Figure 3B). Overall, the stillbirth risks were consistently higher among SGA pregnancies than among AGA or LGA pregnancies throughout the gestational weeks (Figure 3B).

### 3.4 Stillbirth risk ratio (using a fetuses‐at‐risk approach)

**FIGURE 4:**
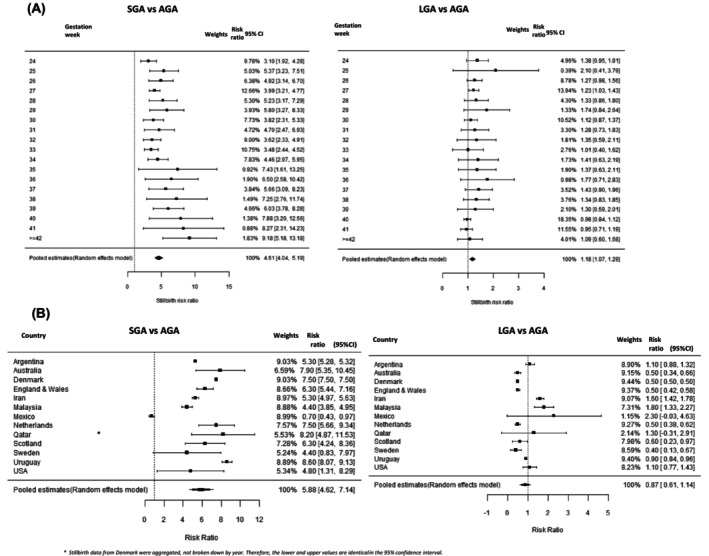
(A) Stillbirth risk ratio comparing small for gestational age (SGA) versus appropriate for gestational age (AGA) and comparing SGA versus large for gestational age (LGA) pregnancies by gestational week using a fetuses‐at‐risk approach across 15 countries, 2000–2020. (B) Stillbirth risk ratio comparing SGA versus AGA and comparing LGA versus AGA pregnancies by country using a fetuses‐at‐risk approach across all gestations (24–44 weeks of gestation), 2000–2020. The square symbol represents the risk ratio for each country, and the size of the square is proportional to the study weight. The whiskers extending from each side of the square represent the range of the 95% confidence interval (95% CI). The diamond symbol indicates the overall pooled effect size with a random‐effect model, which is centred at the point estimate, and the width of the diamond represents the 95% CI. Estonia and Lebanon were excluded from the meta‐analysis because of wide 95% CIs, as their inclusion would compromise the quality of the forest plot.

The stillbirth risk ratios were consistently higher among SGA pregnancies than among AGA pregnancies across all gestations, with the highest risk ratio at post‐term (RR 9.2, 95% CI 5.18–13.18) and with the lowest risk ratio at 24 weeks of gestation (RR 3.1, 95% CI 1.92–4.28) (Figure 4A). The overall stillbirth risk ratio (RR 4.6, 95% CI 4.04–5.19) was also higher among LGA pregnancies compared with AGA pregnancies (Figure 4A).

At the country level, the risk of stillbirth for SGA was around eightfold greater than that for AGA in Uruguay (RR 8.6, 95% CI 8.1–9.1), Qatar (RR 8.2, 95% CI 4.8–11.5), Australia (RR 7.9, 95% CI 5.3–10.4), Denmark (RR 7.5, 95% CI 7.5–7.5) and the Netherlands (RR 7.5, 95% CI 5.6–9.3), was around sixfold greater in England and Wales (RR 6.3, 95% CI 5.4–7.2) and Scotland (RR 6.3, 95% CI 4.2–8.4), was around fivefold greater in Argentina (RR 5.3, 95% CI 5.3–5.3) and the USA (RR 4.8, 95% CI 1.3–8.3) and around fourfold greater in Iran (RR 4.4, 95% CI 3.8–4.9), Malaysia (RR 4.4, 95% CI 3.8–4.9) and Sweden (RR 4.4, 95% CI 0.8–7.9) (Figure 4B; Figure S1A,B and Table S5).

As shown in Figure 4B, we observed strong evidence that the risk of stillbirth was higher in SGA pregnancies than in AGA pregnancies (overall pooled RR 5.9, 95% CI 4.6–7.1), although high heterogeneity was evident (*I* ^2^ = 97.1%). In contrast, we observed no evidence of a difference in the risk of stillbirth between LGA pregnancies and AGA pregnancies, on the basis of the overall pooled result for LGA (RR 0.87, 95% CI 0.61–1.14; *I* ^2^ = 50.5%; *P* < 0.001) (Figure 4B).

## 4 DISCUSSION

This article presents the first multi‐country study to explore variations in size for gestational age‐specific stillbirth risk across pregnancy using a fetuses‐at‐risk approach. Data were included from 15 high‐ and upper‐middle income countries across a range of geographies, encompassing 125 million births and 0.5 million stillbirths. Our findings highlight the increased risk of stillbirth associated with pregnancies resulting in an SGA birth, compared with those for an AGA birth. No such increased stillbirth risk was noted for LGA pregnancies.

The elevated risk of SGA stillbirth persisted across the pregnancy period from 24 to 44 weeks of gestation, with a notable increase between 36 and 42 weeks of gestation, consistent with findings from previous studies. For example, Lavin et al. have found a steady increase in stillbirth risk of all sizes for gestational age after 37 weeks of gestation, and this pattern has also been observed in a study by Gardosi et al., showing a consistent increase in stillbirth risk in SGA pregnancies with advancing gestational age.^10^, ^11^ In contrast, although previous studies have suggested a potential association between LGA and an increased risk of stillbirth, our analysis did not replicate this finding.^11^ It is important to note that our study utilised data from 15 countries and included gestational ages from 24 weeks, whereas the previous study included only term births. Additionally, other confounding factors could have accounted for these discrepancies in the findings. Further research is needed to explore the underlying mechanisms and clarify the relationship between LGA and stillbirth risk. Regarding birth‐based stillbirth rates, the expected decrease after 33 weeks of gestation was consistent with the existing literature, given that preterm birth is well established to be associated with increased perinatal mortality.^21^

This study demonstrated a major difference in gestation‐specific stillbirth risk as measured with the fetuses‐at‐risk approach, compared with the traditional birth‐based stillbirth rate (proportion per 1000 total births). The fetuses‐at‐risk approach provides a more comprehensive assessment of the probability of stillbirth as a pregnancy progresses, potentially providing useful information for planning and decision‐making for individual clinical care that is not available with the birth‐based approach.^13^ However, it is important to note that although the classification based on ‘revealed size at birth’ does detect all cases of SGA at birth, not all will have been recognised antenatally, especially in settings with weaker obstetric maternity services.

The main strength of the study lies in its robust sample size and comprehensive collection of pregnancy‐related variables from multiple countries. However, the variation in clinical care and data contexts across the countries may influence some of the findings. For example, the variation in stillbirth risk ratios, with the highest risks observed between 26 and 29 weeks of gestation, and the lowest risk ratio from term onwards, might potentially be attributed to measurement and recording artefacts, particularly among pregnancies at the earliest gestational ages (24–25 weeks of gestation).^22^ In contrast, the decline in stillbirth risk ratios from term onwards might suggest the influence of interventional obstetric practices in these geographic regions, including access to health care.^23^ These practices may potentially contribute to improved outcomes and decreased stillbirth risks for pregnancies reaching term.

In this study, antenatal fetal size was assessed using the proxy of size at birth. This is likely to result in the misclassification of growth status in the ‘fetuses in utero’ denominator used, especially at earlier gestations, as fetuses are more likely to develop abnormal growth patterns in the third trimester. For example, the denominator for SGA fetuses in utero at 24 weeks of gestation will include all fetuses that were truly SGA at 24 weeks of gestation as well as other AGA fetuses with suboptimal growth later in pregnancy resulting in them being SGA at birth. This is likely to lower the calculated stillbirth risk, and hence underestimate the true stillbirth risk associated with SGA. Future research should focus on prospective studies of fetal growth and survival to address these limitations. Although the results highlight variations in stillbirth risk by fetal size, they also underscore the importance of addressing data quality and measurement issues. Large variation in the definitions used and recording practices for stillbirth was observed across countries. Omission of both live births and stillbirths and potential misclassification with neonatal deaths varies across countries, but in all settings is highest around the thresholds of viability. For example, the Mexican data suggest an under‐capture of births before 28 weeks of gestation (Figure 1A; Table S6).

In addition, recognising that the steepest losses often happen in the first trimester, we initiated our analysis from the 22nd week of gestation.^24^, ^25^ However, in some countries the counts of early‐gestation stillbirths might also contain some cases of induced abortion, especially those occurring between 22 and 24 weeks of gestation.^26^ Given the substantial variation and lack of comparability in reporting births, including stillbirths, at 22 and 23 weeks of gestation, these births were excluded from the final analyses. Another limitation arises from the absence of detailed information on the assessment methods used to determine gestational age in each country included in the analysis. Methods of gestational age assessment vary in accuracy, with an underestimation of gestational age typically seen in assessment based on last menstrual period compared with assessment based on ultrasound, resulting in the potential misclassification of early term births as preterm births. In addition, in some settings the birthweights of fetal deaths may be less reliably recorded than for live births. As classification of births by size for gestation requires birthweight, this may explain the very low stillbirth rate observed in Lebanon, where a higher proportion of fetal deaths compared with live births were excluded for missing birthweights. Furthermore, the inclusion of data from different time periods and the potential presence of unaccounted confounding factors are additional study limitations.

## 5 CONCLUSION

The study revealed that the highest risk ratios were seen at preterm gestations, with more than half of stillbirths occurring at a preterm gestation. SGA was strongly associated with stillbirth risk based on high‐quality data from high‐ and middle‐income countries. To advance our understanding of stillbirth, further analyses using high‐quality data sets from low‐ and lower‐ to middle‐income settings, particularly those with relatively high rates of SGA, will be essential. These findings should support individual antenatal care and programmes to identify high‐risk pregnancies, inform decision‐making and accelerate progress towards the goal of ending preventable stillbirths by 2030.^27^

## AUTHOR CONTRIBUTIONS

The Vulnerable Newborn Measurement Collaboration was conceptualised by JL and Bob Black. All collaborators contributed to the design of the study protocol. YO and HB, with input from JEL and EO, developed the detailed research questions and overall analysis plan for this article. These were refined with inputs from the wider Vulnerable Newborn Measurement Collaboration, LS, JY, V P, GC, AG, VF, EH, HTS, LA, MH, NK, KY, AAB, AB, AEVD, LB, FA, TO, NR, JS, LKS, RJM, RW, KM, IP, GP, SL and QW. Analysis was undertaken by YO and HB, and EB provided statistical oversight. The article was drafted by YO and HB. All authors reviewed and helped to revise the article. All authors reviewed and agreed to publish the final version.

## FUNDING INFORMATION

The Children's Investment Fund Foundation (prime grant 1803–02535) and Bill & Melinda Foundation (INV‐065760) had no role in the study design, data collection, analysis, interpretation of the data or decision to submit for publication.

## CONFLICT OF INTEREST STATEMENT

The authors have no conflict of interest.

## ETHICS APPROVAL

The Vulnerable Newborn Measurement Collaboration was granted ethical approval from the Institutional Review Boards of the London School of Hygiene & Tropical Medicine (ref. 22 858) and Johns Hopkins University. All 15 country teams received ethical approval for the use of data or exemptions based on the current remit (Table S2).

## Supporting information


Appendix S1.

## Data Availability

Data sharing and transfer agreements were jointly developed and signed by all collaborating partners. The pooled summary table data generated during the current study have been deposited online with data access subject to approval at https://doi.org/10.17037/DATA.00003095.

## APPENDIX 1

### National Collaborative Group for Stillbirths Risk and Vulnerable Newborn Types

**Argentina**: Veronica Pingray; Gabriela Cormick; José Belizan; Luz Gibbons; Carlos Guevel

**Australia**: Vicki Flenady; Adrienne Gordon; Kara Warrilow; Harriet Lawford; Jeremy Oats; Michael Humphrey

**Denmark**: Erzsébet Horváth‐Puhó, Henrik T. Sørensen.

**Estonia**: Luule Sakkeus; Liili Abuladze.

**Lebanon**: Khalid A. Yunis; Ayah Al Bizri; Pascale Nakad.

**Malaysia**: Shamala Karalasingam; J Ravichandran; R Jeganathan; Nurakman Binti Baharum.

**Mexico**: Lorena Suárez‐Idueta; Arturo Barranco Flores; Jesus Felipe Gonzalez Roldan; Sonia Lopez Alvarez.

**The Netherlands**: Lisa Broeders; Aimée E. van Dijk.

**Qatar**: Fawziya Alyafei; Mai AlQubaisi; Tawa O. Olukade; Hamdy A. Ali; Mohamad Rami Alturk.

**Sweden**: Neda Razaz; Jonas Söderling.

**UK – England and Wales**: Lucy K Smith; Bradley N. Manktelow; Ruth J. Matthews; Elizabeth Draper; Alan Fenton; Jennifer J. Kurinczuk.

**UK – Scotland**: Rachael Wood; Celina Davis; Kirsten Monteath; Samantha Clarke.

**Uruguay**: Isabel Pereyra, Gabriella Pravia.

**USA**: Sarka Lisonkova; Qi Wen.

### Vulnerable Newborn Measurement Core Group

**LSHTM**: Joy E. Lawn; Hannah Blencowe; Eric Ohuma; Yemisrach B. Okwaraji; Judith Yargawa; Ellen Bradley.

**JHU**: Bob Black; Joanne Katz; Dan Erchick; Elizabeth Hazel; Mike Diaz; Anne CC Lee.
