# Supplementary material for: Stillbirth risk by fetal size among 126.5 million births in 15 countries from 2000 to 2020: A fetuses‐at‐risk approach
Source: BJOG. 2024 Jul 11;132(Suppl 8):S85–96. doi: 10.1111/1471-0528.17890 (PMC12678068; doi:10.1111/1471-0528.17890)
Supplement: Supplementary file 1 — Appendix S1. [file BJO-132-S85-s001.docx]

**SUPPLEMENT TITLE**

Vulnerable Newborn multi-country analyses related to preterm births and small-for-gestational age.

**PAPER TITLE:**

**Stillbirth risk by fetal size amongst 126,543,070 million births in 15 countries from 2000 to 2020: a fetuses-at-risk approach**

**PAPER RUNNING TITLE**

Stillbirth fetuses-at-risk by gestational age in 15 countries.

**SUPPORTING INFORMATION**

Contents

[Table S1. RECORD guidelines checklist 2](#_Toc167196490)

[Table S2. Ethics approval or exemptions of Institutional Review Boards 6](#_Toc167196491)

[Table S3. Overview of input data to inform stillbirth analyses from15 countries, 2000-2020 7](#_Toc167196492)

[Table S4a. Maternal baseline characteristics in 15 countries with stillbirth data 14](#_Toc167196493)

[Table S4b. Maternal baseline characteristics in 15 countries with stillbirth data 16](#_Toc167196494)

[Figure S1a. Stillbirth rate by gestational week for SGA, AGA and LGA pregnancies using birth based approach 18](#_Toc167196495)

[Figure S1b. Stillbirth rate by gestational week for SGA, AGA and LGA pregnancies using fetuses-at-risk approach. 19](#_Toc167196496)

[Table S5. Stillbirth rate per 1000 total birth and stillbirth risk per 1,000 fetuses- still in utero 20](#_Toc167196497)

[Table S6. Summary of metadata 51](#_Toc167196498)

# Table S1. RECORD guidelines checklist

|  | **#** | **STROBE items** | **Location** | **RECORD items** | **Location in manuscript where items are reported** |
| --- | --- | --- | --- | --- | --- |
|  | 1 | (a) Indicate the study’s design with a commonly used term in the title or the abstract (b) Provide in the abstract an informative and balanced summary of what was done and what was found |  | **RECORD 1.1:** The type of data used should be specified in the title or abstract. When possible, the -me of the databases used should be included.  **RECORD 1.2**: If applicable, the geographic region and timeframe within which the study took place should be reported in the title or abstract.  **RECORD 1.3**: If linkage between databases was conducted for the study, this should be clearly stated in the title or abstract. | Title: *“*Stillbirth risk by fetal size amongst 126,543,070‬ million births in 15 countries from 2000 to 2020: a fetuses-at-risk approach  ***”*** |
| Background ratio-le | 2 | Explain the scientific background and rationale for the investigation being reported |  |  | Introduction  (Paragraphs 1-3) |
| Objectives | 3 | State specific objectives, including any prespecified hypotheses |  |  | Introduction  (Paragraph 3) |
| Study Design | 4 | Present key elements of study design early in the paper |  |  | Methods  (Paragraph 1-5) |
| Setting | 5 | Describe the setting, locations, and relevant dates, including periods of recruitment, exposure, follow-up, and data collection |  |  | Methods  (Paragraph 1) |
| Participants | 6 | *(a) Cohort study* - Give the eligibility criteria, and the sources and methods of selection of participants. Describe methods of follow-up  *Case-control study* - Give the eligibility criteria, and the sources and methods of case ascertainment and control selection. Give the ratio-le for the choice of cases and controls.  *Cross sectional study* - Give the eligibility criteria, and the sources and methods of selection of participants.  *(b) Cohort study* - For matched studies, give matching criteria and number of exposed and unexposed  *Case-control study* - For matched studies, give matching criteria and the number of controls per case |  | **RECORD 6.1:** The methods of study population selection (such as codes or algorithms used to identify subjects) should be listed in detail. If this is not possible, an explanation should be provided.  **RECORD 6.2:** Any validation studies of the codes or algorithms used to select the population should be referenced. If validation was conducted for this study and not published elsewhere, detailed methods and results should be provided.  **RECORD 6.3:** If the study involved linkage of databases, consider use of a flow diagram or other graphical display to demonstrate the data linkage process, including the number of individuals with linked data at each stage. | Methods  (Paragraphs 1-5)  Figure 1a. Flowchart |
| Variables | 7 | Clearly define all outcomes, exposures, predictors, potential confounders, and effect modifiers. Give diagnostic criteria, if applicable. |  | **RECORD 7.1:** A complete list of codes and algorithms used to classify exposures, outcomes, confounders, and effect modifiers should be provided. If these cannot be reported, an explanation should be provided. | Methods  (Paragraphs 1-5) |
| Data sources/ measurement | 8 | For each variable of interest, give sources of data and details of methods of assessment (measurement).  Describe comparability of assessment methods if there is more than one group |  |  | Methods  (Paragraphs 2-5) |
| Bias | 9 | Describe any efforts to address potential sources of bias |  |  | Methods  (Paragraph 1)  (Table S1a) |
| Study size | 10 | Explain how the study size was arrived at |  |  | Methods  (Paragraphs 1-5) |
| Quantitative variables | 11 | Explain how quantitative variables were handled in the analyses. If applicable, describe which groupings were chosen, and why |  |  | Methods  (Paragraphs 1-5) |
| Statistical methods | 12 | (a) Describe all statistical methods, including those used to control for confounding. (b) Describe any methods used to examine subgroups and interactions. (c) Explain how missing data were addressed. (d) *Cohort study* - If applicable, explain how loss to follow-up was addressed  *Case-control study* - If applicable, explain how matching of cases and controls was addressed.  *Cross sectional study* - If applicable, describe analytical methods taking account of sampling strategy (e) Describe any sensitivity a-lyses |  |  | Methods  (Paragraphs 1-5) |
| Data access and cleaning methods |  | .. |  | **RECORD 12.1:** Authors should describe the extent to which the investigators had access to the database population used to create the study population.  **RECORD 12.2:** Authors should provide information on the data cleaning methods used in the study. | Methods  (Paragraphs 1-5) |
| Linkage |  | .. |  | **RECORD 12.3:** State whether the study included person-level, institutional-level, or other data linkage across two or more databases. The methods of linkage and methods of linkage quality evaluation should be provided. | Methods  (Paragraphs 1-5) |
| Participants | 13 | (a) Report the numbers of individuals at each stage of the study (*e.g.*, numbers potentially eligible, examined for eligibility, confirmed eligible, included in the study, completing follow-up, and a-lysed)  (b) Give reasons for non-participation at each stage.  (c) Consider use of a flow diagram |  | **RECORD 13.1:** Describe in detail the selection of the persons included in the study (*i.e.,* study population selection) including filtering based on data quality, data availability and linkage. The selection of included persons can be described in the text and/or by means of the study flow diagram. | Results (Paragraph 1 and Figure 1a) |
| Descriptive data | 14 | (a) Give characteristics of study participants (*e.g.*, demographic, clinical, social) and information on exposures and potential confounders. (b) Indicate the number of participants with missing data for each variable of interest  (c) *Cohort study* - summarise follow-up time (*e.g.*, average and total amount) |  |  |  |
| Outcome data | 15 | *Cohort study* - Report numbers of outcome events or summary measures over time  *Case-control study* - Report numbers in each exposure category, or summary measures of exposure  *Cross sectional study* - Report numbers of outcome events or summary measures |  |  |  |
| Main results | 16 | (a) Give unadjusted estimates and, if applicable, confounder-adjusted estimates and their precision (e.g., 95% confidence interval). Make clear which confounders were adjusted for and why they were included. (b) Report category boundaries when continuous variables were categorized. (c) If relevant, consider translating estimates of relative risk into absolute risk for a meaningful time period |  |  |  |
| Other a-lyses | 17 | Report other a-lyses done—e.g., a-lyses of subgroups and interactions, and sensitivity a-lyses |  |  |  |
| Key results | 18 | Summarise key results with reference to study objectives |  |  |  |
| Limitations | 19 | Discuss limitations of the study, taking into account sources of potential bias or imprecision. Discuss both direction and magnitude of any potential bias |  | **RECORD 19.1:** Discuss the implications of using data that were not created or collected to answer the specific research question(s). Include discussion of misclassification bias, unmeasured confounding, missing data, and changing eligibility over time, as they pertain to the study being reported. |  |
| Interpretation | 20 | Give a cautious overall interpretation of results considering objectives, limitations, multiplicity of a-lyses, results from similar studies, and other relevant evidence |  |  |  |
| Generalisability | 21 | Discuss the generalisability (external validity) of the study results |  |  |  |
| Funding | 22 | Give the source of funding and the role of the funders for the present study and, if applicable, for the original study on which the present article is based |  |  | The source of founding is included in the Abstract and the funding role is described at the end of the manuscript under the subheading Funding role |
| Accessibility of protocol, raw data, and programming code |  | .. |  | **RECORD 22.1:** Authors should provide information on how to access any supplemental information such as the study protocol, raw data, or programming code. | Under the subheading Availability of data and material |

Source: Bechamel et al (2015)^4^

# Table S2. Ethics approval or exemptions of Institutional Review Boards

| Country of origin for data | Institutional Review Board(s) or data access provider | Ref/Number | Date of approval |
| --- | --- | --- | --- |
| London School of Hygiene & Tropical Medicine (LSHTM) | LSHTM - Observational / Interventions Research Ethics Committee | 22858 | 17^th^ May 2021 |
| Australia | Australian Institute of Health and Welfare Ethics Committee | EO2018/2/451 | 4^th^ May 2021 |
| Estonia | Ethics Committee of national Institute for Health Development | 770 | 09^th^ August 2021 |
| Iran | Iran University of Medical Sciences, Tehran, Iran | IR.IUMS.REC.1400.758 | 21^st^ November 2021 |
| Lebanon | Institutional Review Board, American University of Beirut | PED.KY.01 | 13^th^ July 2021 |
| Malaysia | Medical Research & Ethics Committee, Ministry of Health Malaysia | KKM/NIHSEC/ P21-718 (4) | 5^the^ May 2021 |
| Mexico | Centre of Investigation in Health Sciences, Anahuac University, Mexico | 202214 | 31^st^ March 2022 |
| Qatar | Medical Research Centre, Hamad Medical Corporation, Doha-Qatar | MRC-01-21-277 | 25^th^ April 2021 |
| UK England and Wales | 1. National Information Governance Board  2. Confidentiality Advisory Group of the Health Research Authority  3. Health & Social Care Information Centre (HSCIC), Data Access Advisory Group | 1. ECC 5-05 (f)/2012  2. 15/CAG/0119  3. DARS-NIC-359651-H3R1P-v5.2. | 10^th^ October 2012 and  1^st^ May2015 |
| UK Scotland | Public Health Scotland | 20210218-Vulnerable Newborn Measurement | 30^th^ March 2021 |
| Exemptions (*e.g., IRB approval not required for public or aggregate data, existing ethics approval in place, etc)* | | | |
| Argentina | | | |
| Denmark | | | |
| The Netherlands | | | |
| Sweden | | | |
| Uruguay | | | |
| USA publicly available data from https://www.cdc.gov/nchs/data_access/Vitalstatsonline.htm | | | |

# Table S3. Overview of input data to inform stillbirth analyses from15 countries, 2000-2020

| **Country** | **Year** | **Live birth** | **Livebirth Missing values** | | | | **Livebirth Excluded values** | | | | | **Fetal deaths (>20 weeks)** | **Stillbirth**  **(>22 weeks)** | **Stillbirth missing values** | | | | **Stillbirth Excluded values** | | | | |
| --- | --- | --- | --- | --- | --- | --- | --- | --- | --- | --- | --- | --- | --- | --- | --- | --- | --- | --- | --- | --- | --- | --- |
|  |  |  | **BW** | **GA** | **BW & GA** | **Sex** | **< 22 weeks** | **>45 weeks** | **< 250g** | **> 6500g** | **Implausible BW** |  |  | **BW** | **GA** | **BW & GA** | **Sex** | **< 22 weeks** | **>45 weeks** | **< 250g** | **> 6500g** | **Implausible BW** |
| Argentina | 2017 | 628,373 | 0.67 | 1.25 | 0.28 | 0.69 | 0.02 | 0.03 | 0.00 | 0.00 | 0.02 | 5,832 | 4,709 | 6.00 | 3.43 | 2.37 | 5.61 | 12.98 | 0.00 | 4.92 | 0.00 | 0.33 |
| Argentina | 2018 | 610,080 | 0.56 | 0.89 | 0.22 | 0.92 | 0.02 | 0.02 | 0.00 | 0.00 | 0.03 | 5,869 | 4,588 | 7.55 | 3.75 | 2.56 | 7.29 | 13.67 | 0.00 | 5.06 | 0.00 | 0.03 |
| Australia | 2000 | 255,036 | 0.04 | 0.02 | 0.00 | 0.01 | 0.04 | 0.00 | 0.00 | 0.00 | 0.02 | 1,807 | 1,373 | 1.49 | 0.00 | 0.00 | 1.49 | 20.37 | 0.00 | 0.00 | 0.00 | 0.33 |
| Australia | 2001 | 252,063 | 0.02 | 0.01 | 0.00 | 0.03 | 0.06 | 0.00 | 0.00 | 0.00 | 0.01 | 1,754 | 1,259 | 2.05 | 0.00 | 0.00 | 1.65 | 20.98 | 0.00 | 0.00 | 0.00 | 0.46 |
| Australia | 2002 | 252,833 | 0.02 | 0.01 | 0.00 | 0.04 | 0.04 | 0.00 | 0.00 | 0.00 | 0.03 | 1,707 | 1,223 | 1.64 | 0.00 | 0.00 | 1.35 | 21.27 | 0.00 | 0.00 | 0.00 | 0.00 |
| Australia | 2003 | 254,781 | 0.02 | 0.01 | 0.00 | 0.03 | 0.05 | 0.00 | 0.00 | 0.00 | 0.02 | 1,826 | 1,292 | 2.79 | 0.00 | 0.00 | 2.25 | 21.58 | 0.00 | 0.00 | 0.00 | 0.00 |
| Australia | 2004 | 254,834 | 0.01 | 0.01 | 0.00 | 0.01 | 0.06 | 0.00 | 0.00 | 0.00 | 0.01 | 1,919 | 1,371 | 3.86 | 0.00 | 0.00 | 2.08 | 23.40 | 0.00 | 0.00 | 0.00 | 0.00 |
| Australia | 2005 | 270,049 | 0.01 | 0.00 | 0.00 | 0.04 | 0.06 | 0.00 | 0.00 | 0.00 | 0.02 | 1,981 | 1,350 | 3.13 | 0.76 | 0.00 | 1.72 | 24.68 | 0.00 | 0.00 | 0.00 | 0.25 |
| Australia | 2006 | 279,600 | 0.03 | 0.01 | 0.00 | 0.04 | 0.05 | 0.00 | 0.00 | 0.00 | 0.02 | 2,094 | 1,463 | 3.15 | 0.00 | 0.00 | 1.34 | 24.59 | 0.00 | 0.00 | 0.00 | 0.00 |
| Australia | 2007 | 291,554 | 0.03 | 0.02 | 0.00 | 0.02 | 0.06 | 0.00 | 0.00 | 0.00 | 0.03 | 2,180 | 1,476 | 3.35 | 1.06 | 0.00 | 2.06 | 26.65 | 0.00 | 0.00 | 0.00 | 0.00 |
| Australia | 2008 | 294,157 | 0.02 | 0.01 | 0.00 | 0.01 | 0.05 | 0.00 | 0.00 | 0.00 | 0.02 | 2,189 | 1,508 | 2.19 | 0.27 | 0.00 | 2.70 | 25.45 | 0.00 | 0.00 | 0.00 | 0.00 |
| Australia | 2009 | 295,640 | 0.04 | 0.12 | 0.00 | 0.08 | 0.06 | 0.00 | 0.00 | 0.00 | 0.04 | 2,348 | 1,608 | 3.92 | 0.77 | 0.00 | 3.11 | 25.85 | 0.00 | 0.00 | 0.00 | 0.30 |
| Australia | 2010 | 297,318 | 0.07 | 0.02 | 0.00 | 0.05 | 0.06 | 0.00 | 0.00 | 0.00 | 0.03 | 2,201 | 1,507 | 3.13 | 0.00 | 0.00 | 2.77 | 25.67 | 0.00 | 0.00 | 0.00 | 0.00 |
| Australia | 2011 | 299,116 | 0.07 | 0.02 | 0.00 | 0.03 | 0.05 | 0.00 | 0.00 | 0.00 | 0.03 | 2,230 | 1,484 | 2.74 | 0.00 | 0.00 | 3.50 | 27.17 | 0.00 | 0.00 | 0.00 | 0.00 |
| Australia | 2012 | 309,445 | 0.04 | 0.02 | 0.00 | 0.01 | 0.05 | 0.00 | 0.00 | 0.00 | 0.03 | 2,255 | 1,496 | 3.33 | 0.49 | 0.31 | 4.48 | 27.01 | 0.00 | 0.00 | 0.00 | 0.00 |
| Australia | 2013 | 306,638 | 0.04 | 0.02 | 0.00 | 0.01 | 0.06 | 0.00 | 0.00 | 0.00 | 0.02 | 2,191 | 1,481 | 3.24 | 0.37 | 0.00 | 5.52 | 27.25 | 0.00 | 0.00 | 0.00 | 0.23 |
| Australia | 2014 | 309,696 | 0.04 | 0.06 | 0.01 | 0.02 | 0.06 | 0.00 | 0.00 | 0.00 | 0.01 | 2,200 | 1,457 | 3.00 | 1.86 | 1.86 | 4.86 | 26.77 | 0.00 | 0.00 | 0.00 | 0.00 |
| Australia | 2015 | 306,228 | 0.03 | 0.02 | 0.00 | 0.01 | 0.05 | 0.00 | 0.00 | 0.00 | 0.02 | 2,160 | 1,450 | 2.87 | 0.00 | 0.00 | 2.96 | 27.64 | 0.00 | 0.00 | 0.00 | 0.00 |
| Australia | 2016 | 312,093 | 0.04 | 0.08 | 0.00 | 0.01 | 0.05 | 0.00 | 0.00 | 0.00 | 0.02 | 2,107 | 1,404 | 2.61 | 0.19 | 0.00 | 3.46 | 28.43 | 0.00 | 7.55 | 0.00 | 0.00 |
| Australia | 2017 | 303,001 | 0.03 | 0.03 | 0.00 | 0.01 | 0.06 | 0.00 | 0.00 | 0.00 | 0.02 | 2,174 | 1,476 | 1.66 | 0.14 | 0.00 | 3.63 | 27.69 | 0.00 | 8.19 | 0.00 | 0.00 |
| Australia | 2018 | 300,477 | 0.05 | 0.03 | 0.00 | 0.01 | 0.05 | 0.00 | 0.00 | 0.00 | 0.02 | 2,118 | 1,401 | 2.79 | 0.09 | 0.00 | 4.63 | 27.53 | 0.00 | 5.85 | 0.00 | 0.00 |
| Australia | 2019 | 300,358 | 0.06 | 0.04 | 0.00 | 0.01 | 0.05 | 0.00 | 0.00 | 0.00 | 0.01 | 2,183 | 1,449 | 3.30 | 0.00 | 0.00 | 5.68 | 27.71 | 0.00 | 7.74 | 0.00 | 0.00 |
| Denmark | 2000-2013 | 867,290 | 1.87 | 1.73 | 1.46 | 0.01 | 0.03 | 0.00 | 0.01 | 0.00 | 0.03 | 3,723 | 2,969 | 16.57 | 2.63 | 2.39 | 13.73 | 0.00 | 0.00 | 2.36 | 0.00 | 0.00 |
| England & Wales | 2015 | 666,881 | 0.00 | 0.00 | 0.00 | 0.00 | 0.00 | 0.00 | 0.00 | 0.00 | 0.00 | 3,027 | 3,026 | 0.00 | 0.00 | 0.00 | 0.00 | 0.00 | 0.00 | 0.00 | 0.00 | 0.00 |
| England & Wales | 2016 | 666,539 | 0.00 | 0.00 | 0.00 | 0.00 | 0.00 | 0.00 | 0.00 | 0.00 | 0.00 | 3,009 | 3,008 | 0.00 | 0.00 | 0.00 | 0.00 | 0.00 | 0.00 | 0.00 | 0.00 | 0.00 |
| England & Wales | 2017 | 649,066 | 0.00 | 0.00 | 0.00 | 0.00 | 0.00 | 0.00 | 0.00 | 0.00 | 0.00 | 2,775 | 2,773 | 0.00 | 0.00 | 0.00 | 0.00 | 0.00 | 0.00 | 0.00 | 0.00 | 0.00 |
| England & Wales | 2018 | 621,468 | 0.00 | 0.00 | 0.00 | 0.00 | 0.00 | 0.00 | 0.00 | 0.00 | 0.00 | 2,580 | 2,578 | 0.00 | 0.00 | 0.00 | 0.00 | 0.00 | 0.00 | 0.00 | 0.00 | 0.00 |
| England & Wales | 2019 | 608,538 | 0.00 | 0.00 | 0.00 | 0.00 | 0.00 | 0.00 | 0.00 | 0.00 | 0.00 | 2,441 | 2,441 | 0.00 | 0.00 | 0.00 | 0.00 | 0.00 | 0.00 | 0.00 | 0.00 | 0.00 |
| Estonia | 2015 | 13,911 | 0.00 | 0.00 | 0.00 | 0.00 | 0.00 | 0.00 | 0.00 | 0.00 | 0.00 | 54 | 52 | 0.00 | 0.00 | 0.00 | 0.00 | 0.00 | 0.00 | 0.00 | 0.00 | 0.00 |
| Estonia | 2016 | 13,872 | 0.00 | 0.00 | 0.00 | 0.00 | 0.00 | 0.00 | 0.00 | 0.00 | 0.00 | 49 | 49 | 0.00 | 0.00 | 0.00 | 0.00 | 0.00 | 0.00 | 0.00 | 0.00 | 0.00 |
| Estonia | 2017 | 13,519 | 0.00 | 0.00 | 0.00 | 0.00 | 0.00 | 0.00 | 0.00 | 0.00 | 0.00 | 45 | 42 | 0.00 | 0.00 | 0.00 | 0.00 | 0.00 | 0.00 | 0.00 | 0.00 | 0.00 |
| Estonia | 2018 | 14,186 | 0.00 | 0.00 | 0.00 | 0.00 | 0.00 | 0.00 | 0.00 | 0.00 | 0.00 | 46 | 46 | 0.00 | 0.00 | 0.00 | 0.00 | 0.00 | 0.00 | 0.00 | 0.00 | 0.00 |
| Estonia | 2019 | 13,909 | 0.00 | 0.00 | 0.00 | 0.00 | 0.00 | 0.00 | 0.00 | 0.00 | 0.00 | 27 | 25 | 0.00 | 0.00 | 0.00 | 0.00 | 0.00 | 0.00 | 0.00 | 0.00 | 0.00 |
| Estonia | 2020 | 13,030 | 0.00 | 0.00 | 0.00 | 0.00 | 0.00 | 0.00 | 0.00 | 0.00 | 0.00 | 30 | 30 | 0.00 | 0.00 | 0.00 | 0.00 | 0.00 | 0.00 | 0.00 | 0.00 | 0.00 |
| Iran | 2017 | 1,170,242 | 0.00 | 0.05 | 0.00 | 0.00 | 0.00 | 0.00 | 0.01 | 0.00 | 0.11 | 8,882 | 8,734 | 0.00 | 0.00 | 0.00 | 0.00 | 0.00 | 0.00 | 1.15 | 0.07 | 0.51 |
| Iran | 2018 | 1,430,979 | 0.00 | 0.01 | 0.00 | 0.00 | 0.00 | 0.00 | 0.02 | 0.00 | 0.12 | 10,831 | 10,678 | 0.00 | 0.00 | 0.00 | 0.00 | 0.00 | 0.00 | 1.02 | 0.03 | 0.39 |
| Iran | 2019 | 1,274,706 | 0.02 | 0.02 | 0.01 | 0.04 | 0.00 | 0.00 | 0.02 | 0.00 | 0.10 | 9,604 | 9,451 | 0.17 | 0.08 | 0.05 | 0.05 | 0.00 | 0.00 | 0.99 | 0.02 | 0.37 |
| Iran | 2020 | 936,701 | 0.07 | 0.08 | 0.06 | 0.09 | 0.00 | 0.00 | 0.02 | 0.00 | 0.08 | 9,771 | 9,599 | 0.40 | 0.20 | 0.15 | 0.19 | 0.00 | 0.01 | 1.01 | 0.04 | 0.21 |
| Lebanon | 2003 | 6,787 | 1.77 | 2.18 | 0.68 | 2.25 | 0.00 | 0.00 | 0.00 | 0.00 | 0.06 | 31 | 21 | 0.00 | 0.00 | 0.00 | 6.45 | 29.03 | 0.00 | 0.00 | 0.00 | 0.00 |
| Lebanon | 2004 | 9,018 | 1.85 | 4.25 | 1.16 | 1.62 | 0.00 | 0.04 | 0.00 | 0.00 | 0.01 | 33 | 24 | 3.03 | 3.03 | 3.03 | 0.00 | 15.15 | 0.00 | 9.09 | 0.00 | 0.00 |
| Lebanon | 2005 | 12,606 | 1.73 | 2.68 | 0.71 | 3.93 | 0.00 | 0.00 | 0.00 | 0.00 | 0.02 | 34 | 24 | 11.76 | 5.88 | 5.88 | 8.82 | 14.71 | 0.00 | 5.88 | 0.00 | 0.00 |
| Lebanon | 2006 | 14,070 | 1.61 | 2.32 | 0.37 | 2.27 | 0.00 | 0.00 | 0.00 | 0.00 | 0.03 | 27 | 22 | 0.00 | 0.00 | 0.00 | 0.00 | 14.81 | 0.00 | 7.41 | 0.00 | 0.00 |
| Lebanon | 2007 | 14,872 | 2.68 | 2.32 | 0.69 | 4.16 | 0.00 | 0.00 | 0.00 | 0.00 | 0.01 | 3 | 3 | 0.00 | 0.00 | 0.00 | 0.00 | 0.00 | 0.00 | 0.00 | 0.00 | 0.00 |
| Lebanon | 2008 | 16,834 | 2.47 | 3.12 | 0.92 | 3.15 | 0.00 | 0.01 | 0.00 | 0.00 | 0.03 | 25 | 19 | 8.00 | 0.00 | 0.00 | 12.00 | 0.00 | 0.00 | 0.00 | 0.00 | 0.00 |
| Lebanon | 2009 | 18,600 | 3.79 | 7.60 | 2.91 | 4.07 | 0.00 | 0.00 | 0.00 | 0.00 | 0.01 | 78 | 54 | 12.82 | 1.28 | 0.00 | 0.00 | 14.10 | 0.00 | 7.69 | 0.00 | 0.00 |
| Lebanon | 2010 | 20,135 | 3.43 | 4.98 | 2.44 | 4.28 | 0.00 | 0.00 | 0.00 | 0.00 | 0.03 | 84 | 55 | 15.48 | 0.00 | 0.00 | 4.76 | 15.48 | 0.00 | 8.33 | 0.00 | 1.19 |
| Lebanon | 2011 | 20,770 | 4.87 | 6.34 | 3.38 | 5.08 | 0.00 | 0.01 | 0.00 | 0.00 | 0.02 | 71 | 50 | 9.86 | 1.41 | 0.00 | 1.41 | 16.90 | 0.00 | 4.23 | 0.00 | 0.00 |
| Lebanon | 2012 | 23,852 | 6.05 | 7.15 | 4.21 | 5.35 | 0.00 | 0.00 | 0.00 | 0.00 | 0.00 | 65 | 41 | 12.31 | 16.92 | 3.08 | 12.31 | 7.69 | 0.00 | 0.00 | 0.00 | 0.00 |
| Lebanon | 2013 | 25,022 | 6.43 | 7.46 | 4.95 | 5.93 | 0.00 | 0.01 | 0.00 | 0.00 | 0.04 | 103 | 64 | 4.85 | 4.85 | 0.97 | 9.71 | 22.33 | 0.00 | 3.88 | 0.00 | 0.97 |
| Lebanon | 2014 | 20,531 | 7.36 | 8.20 | 6.45 | 7.83 | 0.00 | 0.00 | 0.00 | 0.00 | 0.01 | 52 | 36 | 11.54 | 1.92 | 1.92 | 11.54 | 13.46 | 0.00 | 0.00 | 0.00 | 0.00 |
| Lebanon | 2015 | 17,226 | 9.86 | 10.10 | 8.43 | 10.64 | 0.00 | 0.00 | 0.00 | 0.00 | 0.01 | 24 | 16 | 25.00 | 0.00 | 0.00 | 4.17 | 8.33 | 0.00 | 0.00 | 0.00 | 0.00 |
| Lebanon | 2016 | 16,916 | 7.11 | 7.31 | 5.79 | 8.31 | 0.00 | 0.01 | 0.00 | 0.00 | 0.02 | 29 | 20 | 10.34 | 0.00 | 0.00 | 0.00 | 31.03 | 0.00 | #### | 0.00 | 0.00 |
| Lebanon | 2017 | 15,810 | 2.84 | 2.89 | 1.79 | 3.33 | 0.00 | 0.00 | 0.00 | 0.00 | 0.03 | 38 | 18 | 10.53 | 0.00 | 0.00 | 2.63 | 42.11 | 0.00 | 5.26 | 0.00 | 2.63 |
| Lebanon | 2018 | 16,167 | 2.94 | 2.44 | 1.78 | 3.04 | 0.00 | 0.01 | 0.00 | 0.00 | 0.04 | 28 | 16 | 17.86 | 7.14 | 3.57 | 10.71 | 17.86 | 0.00 | #### | 0.00 | 3.57 |
| Lebanon | 2019 | 13,458 | 2.01 | 1.85 | 0.97 | 2.36 | 0.01 | 0.03 | 0.00 | 0.01 | 0.07 | 29 | 15 | 27.59 | 0.00 | 0.00 | 24.14 | 13.79 | 0.00 | 0.00 | 0.00 | 0.00 |
| Malaysia | 2011 | 94,814 | 1.00 | 37.70 | 0.51 | 1.43 | 0.01 | 0.01 | 0.05 | 0.01 | 0.23 | 974 | 731 | 2.05 | 17.25 | 0.51 | 1.95 | 1.03 | 0.10 | 1.64 | 0.31 | 0.21 |
| Malaysia | 2012 | 111,327 | 0.49 | 8.69 | 0.05 | 0.94 | 0.00 | 0.00 | 0.04 | 0.01 | 0.22 | 904 | 793 | 0.44 | 5.53 | 0.00 | 1.55 | 0.55 | 0.44 | 1.55 | 0.00 | 0.00 |
| Malaysia | 2013 | 111,444 | 0.16 | 4.66 | 0.01 | 0.03 | 0.00 | 0.00 | 0.07 | 0.01 | 0.32 | 886 | 752 | 0.45 | 7.90 | 0.00 | 1.02 | 0.79 | 0.11 | 2.82 | 0.23 | 0.23 |
| Malaysia | 2014 | 127,594 | 0.97 | 6.42 | 0.11 | 0.09 | 0.00 | 0.00 | 0.05 | 0.01 | 0.28 | 1,002 | 825 | 1.60 | 10.08 | 0.40 | 1.70 | 1.60 | 0.10 | 1.70 | 0.00 | 0.20 |
| Malaysia | 2015 | 132,064 | 1.80 | 8.83 | 0.13 | 0.13 | 0.01 | 0.00 | 0.05 | 0.01 | 0.31 | 1,080 | 875 | 2.87 | 9.35 | 0.28 | 1.57 | 1.85 | 0.00 | 1.85 | 0.37 | 0.46 |
| Malaysia | 2016 | 125,268 | 2.09 | 9.08 | 0.17 | 0.11 | 0.01 | 0.00 | 0.05 | 0.00 | 0.23 | 1,068 | 889 | 3.00 | 8.80 | 0.47 | 1.69 | 1.59 | 0.00 | 1.50 | 0.00 | 0.09 |
| Malaysia | 2017 | 112,193 | 0.39 | 33.01 | 0.13 | 0.02 | 0.00 | 0.00 | 0.04 | 0.00 | 0.19 | 1,209 | 1,100 | 1.41 | 0.91 | 0.00 | 1.16 | 1.32 | 0.00 | 2.23 | 0.00 | 0.08 |
| Mexico | 2008 | 1,835,062 | 6.65 | 1.11 | 0.08 | 0.18 | 0.02 | 0.00 | 0.00 | 0.00 | 0.01 | 17,397 | 17,155 | 0.00 | 0.00 | 0.00 | 1.39 | 0.00 | 0.01 | 0.00 | 0.00 | 0.00 |
| Mexico | 2009 | 1,913,409 | 6.72 | 0.85 | 0.07 | 0.13 | 0.02 | 0.00 | 0.00 | 0.00 | 0.01 | 16,992 | 16,742 | 0.00 | 0.00 | 0.00 | 1.47 | 0.00 | 0.00 | 0.00 | 0.00 | 0.00 |
| Mexico | 2010 | 1,931,096 | 6.69 | 0.62 | 0.06 | 0.14 | 0.03 | 0.00 | 0.00 | 0.00 | 0.01 | 16,732 | 16,492 | 0.00 | 0.00 | 0.00 | 1.43 | 0.00 | 0.00 | 0.00 | 0.00 | 0.00 |
| Mexico | 2011 | 2,024,743 | 6.51 | 0.48 | 0.07 | 0.14 | 0.02 | 0.00 | 0.00 | 0.00 | 0.01 | 16,575 | 16,337 | 0.00 | 0.00 | 0.00 | 1.44 | 0.00 | 0.00 | 0.00 | 0.00 | 0.00 |
| Mexico | 2012 | 2,067,890 | 6.27 | 0.41 | 0.05 | 0.12 | 0.02 | 0.00 | 0.00 | 0.00 | 0.01 | 15,922 | 15,710 | 0.00 | 0.00 | 0.00 | 1.33 | 0.00 | 0.00 | 0.00 | 0.00 | 0.00 |
| Mexico | 2013 | 2,056,273 | 6.39 | 0.32 | 0.05 | 0.11 | 0.02 | 0.00 | 0.00 | 0.00 | 0.01 | 15,449 | 15,234 | 0.00 | 0.00 | 0.00 | 1.39 | 0.00 | 0.00 | 0.00 | 0.00 | 0.00 |
| Mexico | 2014 | 2,043,653 | 6.26 | 0.23 | 0.03 | 0.09 | 0.01 | 0.00 | 0.00 | 0.00 | 0.01 | 15,100 | 14,860 | 0.01 | 0.00 | 0.00 | 1.58 | 0.00 | 0.00 | 0.00 | 0.00 | 0.00 |
| Mexico | 2015 | 2,019,716 | 5.96 | 0.20 | 0.02 | 0.08 | 0.01 | 0.00 | 0.00 | 0.00 | 0.00 | 15,198 | 14,783 | 0.82 | 0.00 | 0.00 | 1.97 | 0.00 | 0.00 | 0.00 | 0.00 | 0.00 |
| Mexico | 2016 | 1,962,380 | 5.80 | 0.14 | 0.02 | 0.09 | 0.01 | 0.00 | 0.00 | 0.00 | 0.01 | 14,439 | 14,078 | 0.84 | 0.00 | 0.00 | 1.71 | 0.00 | 0.00 | 0.00 | 0.00 | 0.00 |
| Mexico | 2017 | 1,947,994 | 5.82 | 0.11 | 0.01 | 0.08 | 0.01 | 0.00 | 0.00 | 0.00 | 0.00 | 14,575 | 14,182 | 0.99 | 0.00 | 0.00 | 1.76 | 0.00 | 0.00 | 0.00 | 0.00 | 0.00 |
| Mexico | 2018 | 1,848,682 | 4.75 | 0.16 | 0.01 | 0.08 | 0.01 | 0.00 | 0.01 | 0.00 | 0.02 | 15,412 | 14,965 | 1.12 | 0.00 | 0.00 | 1.82 | 0.00 | 0.00 | 0.02 | 0.00 | 0.03 |
| Mexico | 2019 | 1,763,891 | 5.70 | 0.16 | 0.01 | 0.08 | 0.01 | 0.00 | 0.00 | 0.00 | 0.01 | 15,151 | 14,842 | 0.24 | 0.00 | 0.00 | 1.85 | 0.00 | 0.00 | 0.00 | 0.00 | 0.00 |
| The Netherlands | 2010 | 176,272 | 0.07 | 0.59 | 0.00 | 0.00 | 0.00 | 0.00 | 0.00 | 0.00 | 0.01 | 1,012 | 938 | 1.88 | 0.30 | 0.00 | 1.78 | 0.00 | 0.00 | 4.45 | 0.00 | 0.20 |
| The Netherlands | 2011 | 174,276 | 0.09 | 0.61 | 0.00 | 0.00 | 0.00 | 0.00 | 0.00 | 0.00 | 0.01 | 971 | 896 | 2.27 | 0.51 | 0.00 | 1.65 | 0.00 | 0.00 | 4.33 | 0.00 | 0.21 |
| The Netherlands | 2012 | 171,470 | 0.09 | 1.08 | 0.00 | 0.00 | 0.00 | 0.00 | 0.00 | 0.00 | 0.01 | 957 | 888 | 1.99 | 0.21 | 0.00 | 1.46 | 0.00 | 0.00 | 4.81 | 0.00 | 0.00 |
| The Netherlands | 2013 | 166,799 | 0.10 | 0.71 | 0.00 | 0.00 | 0.00 | 0.00 | 0.01 | 0.00 | 0.02 | 863 | 759 | 3.94 | 0.70 | 0.00 | 2.20 | 0.00 | 0.00 | 7.30 | 0.00 | 0.00 |
| The Netherlands | 2014 | 170,467 | 0.11 | 0.86 | 0.00 | 0.00 | 0.00 | 0.00 | 0.00 | 0.01 | 0.02 | 829 | 752 | 2.77 | 0.84 | 0.00 | 1.93 | 0.00 | 0.00 | 4.83 | 0.00 | 0.00 |
| The Netherlands | 2015 | 165,454 | 0.12 | 0.70 | 0.00 | 0.00 | 0.00 | 0.00 | 0.00 | 0.00 | 0.02 | 862 | 774 | 3.60 | 0.93 | 0.00 | 2.09 | 0.00 | 0.00 | 4.99 | 0.00 | 0.12 |
| The Netherlands | 2016 | 168,397 | 0.12 | 0.55 | 0.00 | 0.00 | 0.00 | 0.00 | 0.00 | 0.00 | 0.01 | 791 | 698 | 3.79 | 1.64 | 0.00 | 2.40 | 0.00 | 0.00 | 5.56 | 0.00 | 0.13 |
| The Netherlands | 2017 | 164,952 | 0.16 | 0.68 | 0.00 | 0.00 | 0.00 | 0.00 | 0.00 | 0.00 | 0.01 | 819 | 744 | 3.17 | 0.49 | 0.00 | 3.05 | 0.00 | 0.00 | 4.27 | 0.00 | 0.00 |
| The Netherlands | 2018 | 160,107 | 0.29 | 0.65 | 0.00 | 0.00 | 0.00 | 0.00 | 0.00 | 0.00 | 0.01 | 927 | 825 | 5.18 | 1.62 | 0.00 | 1.94 | 0.00 | 0.00 | 3.34 | 0.00 | 0.11 |
| The Netherlands | 2019 | 163,035 | 0.13 | 0.65 | 0.00 | 0.01 | 0.00 | 0.00 | 0.00 | 0.00 | 0.01 | 921 | 851 | 2.17 | 1.09 | 0.00 | 2.06 | 0.00 | 0.00 | 3.69 | 0.00 | 0.00 |
| The Netherlands | 2020 | 163,536 | 0.10 | 0.37 | 0.00 | 0.01 | 0.00 | 0.00 | 0.00 | 0.00 | 0.01 | 942 | 872 | 2.76 | 0.21 | 0.00 | 1.59 | 0.00 | 0.00 | 3.29 | 0.00 | 0.21 |
| Qatar | 2016 | 22,035 | 0.36 | 2.03 | 0.02 | 0.00 | 0.01 | 0.00 | 0.00 | 0.00 | 0.03 | 63 | 55 | 3.17 | 0.00 | 0.00 | 4.76 | 4.76 | 0.00 | 1.59 | 0.00 | 0.00 |
| Qatar | 2017 | 23,936 | 0.38 | 1.29 | 0.03 | 0.00 | 0.00 | 0.00 | 0.00 | 0.00 | 0.03 | 202 | 171 | 2.97 | 0.00 | 0.00 | 2.48 | 6.93 | 0.00 | 5.45 | 0.00 | 0.00 |
| Qatar | 2018 | 23,549 | 0.36 | 1.11 | 0.02 | 0.01 | 0.03 | 0.00 | 0.00 | 0.00 | 0.01 | 170 | 149 | 4.12 | 0.59 | 0.00 | 4.12 | 3.53 | 0.00 | 3.53 | 0.00 | 1.76 |
| Qatar | 2019 | 24,817 | 0.38 | 0.74 | 0.00 | 0.00 | 0.01 | 0.00 | 0.00 | 0.00 | 0.02 | 152 | 134 | 5.26 | 1.97 | 0.00 | 5.26 | 5.26 | 0.00 | 1.32 | 0.00 | 0.00 |
| Scotland | 2000 | 52,462 | 0.04 | 0.03 | 0.00 | 0.01 | 0.00 | 0.00 | 0.00 | 0.00 | 0.02 | 321 | 270 | 13.71 | 0.31 | 0.00 | 15.26 | 0.00 | 0.00 | 0.31 | 0.00 | 0.00 |
| Scotland | 2001 | 51,247 | 0.02 | 0.03 | 0.00 | 0.01 | 0.01 | 0.00 | 0.00 | 0.00 | 0.02 | 338 | 284 | 12.72 | 0.00 | 0.00 | 13.91 | 0.00 | 0.00 | 2.07 | 0.00 | 0.00 |
| Scotland | 2002 | 50,399 | 0.03 | 0.04 | 0.00 | 0.00 | 0.00 | 0.00 | 0.00 | 0.00 | 0.04 | 308 | 257 | 13.96 | 0.00 | 0.00 | 14.61 | 0.00 | 0.00 | 1.62 | 0.00 | 0.00 |
| Scotland | 2003 | 51,515 | 0.05 | 0.04 | 0.00 | 0.01 | 0.00 | 0.00 | 0.00 | 0.00 | 0.03 | 330 | 267 | 16.06 | 0.00 | 0.00 | 16.67 | 0.00 | 0.00 | 1.52 | 0.00 | 0.00 |
| Scotland | 2004 | 53,044 | 0.03 | 0.04 | 0.00 | 0.01 | 0.01 | 0.00 | 0.02 | 0.00 | 0.04 | 350 | 300 | 10.86 | 0.00 | 0.00 | 12.57 | 0.00 | 0.00 | 0.86 | 0.00 | 0.00 |
| Scotland | 2005 | 52,756 | 0.09 | 0.04 | 0.00 | 0.01 | 0.00 | 0.00 | 0.00 | 0.00 | 0.02 | 310 | 257 | 11.94 | 0.00 | 0.00 | 15.16 | 0.00 | 0.00 | 1.94 | 0.00 | 0.00 |
| Scotland | 2006 | 53,417 | 0.07 | 0.05 | 0.00 | 0.01 | 0.02 | 0.00 | 0.00 | 0.00 | 0.03 | 314 | 266 | 10.19 | 0.00 | 0.00 | 11.15 | 0.00 | 0.00 | 1.91 | 0.00 | 0.64 |
| Scotland | 2007 | 56,395 | 0.13 | 0.06 | 0.00 | 0.02 | 0.03 | 0.00 | 0.01 | 0.00 | 0.04 | 363 | 308 | 10.74 | 0.00 | 0.00 | 13.77 | 0.00 | 0.00 | 2.20 | 0.00 | 0.00 |
| Scotland | 2008 | 58,543 | 0.11 | 0.08 | 0.00 | 0.01 | 0.02 | 0.00 | 0.01 | 0.00 | 0.01 | 344 | 298 | 9.59 | 0.29 | 0.29 | 12.50 | 0.00 | 0.00 | 0.87 | 0.00 | 0.00 |
| Scotland | 2009 | 57,739 | 0.11 | 0.08 | 0.00 | 0.01 | 0.02 | 0.00 | 0.00 | 0.00 | 0.03 | 361 | 300 | 14.13 | 0.00 | 0.00 | 14.13 | 0.00 | 0.00 | 1.94 | 0.00 | 0.55 |
| Scotland | 2010 | 57,795 | 0.03 | 0.05 | 0.00 | 0.00 | 0.01 | 0.00 | 0.00 | 0.00 | 0.02 | 310 | 262 | 13.55 | 0.00 | 0.00 | 14.19 | 0.00 | 0.00 | 1.29 | 0.00 | 0.00 |
| Scotland | 2011 | 57,447 | 0.01 | 0.06 | 0.00 | 0.00 | 0.00 | 0.00 | 0.01 | 0.00 | 0.01 | 324 | 280 | 11.73 | 0.00 | 0.00 | 12.04 | 0.00 | 0.00 | 1.85 | 0.00 | 0.00 |
| Scotland | 2012 | 56,766 | 0.06 | 0.09 | 0.01 | 0.00 | 0.02 | 0.00 | 0.01 | 0.00 | 0.01 | 297 | 248 | 15.49 | 0.00 | 0.00 | 15.15 | 0.00 | 0.00 | 0.67 | 0.00 | 0.00 |
| Scotland | 2013 | 54,870 | 0.17 | 0.13 | 0.00 | 0.01 | 0.01 | 0.00 | 0.00 | 0.00 | 0.03 | 259 | 216 | 15.44 | 0.00 | 0.00 | 14.29 | 0.00 | 0.00 | 0.77 | 0.39 | 0.39 |
| Scotland | 2014 | 55,562 | 0.15 | 0.44 | 0.02 | 0.02 | 0.01 | 0.00 | 0.00 | 0.00 | 0.03 | 251 | 218 | 11.55 | 0.40 | 0.40 | 12.35 | 0.00 | 0.00 | 0.80 | 0.00 | 0.00 |
| Scotland | 2015 | 54,095 | 0.11 | 0.44 | 0.01 | 0.00 | 0.01 | 0.00 | 0.01 | 0.00 | 0.03 | 225 | 193 | 12.00 | 0.00 | 0.00 | 12.44 | 0.00 | 0.00 | 2.22 | 0.00 | 0.00 |
| Scotland | 2016 | 53,242 | 0.21 | 0.79 | 0.00 | 0.02 | 0.02 | 0.00 | 0.00 | 0.00 | 0.03 | 249 | 212 | 13.65 | 0.00 | 0.00 | 13.25 | 0.00 | 0.00 | 1.20 | 0.00 | 0.00 |
| Scotland | 2017 | 51,643 | 0.56 | 0.52 | 0.02 | 0.03 | 0.01 | 0.00 | 0.00 | 0.00 | 0.03 | 229 | 202 | 10.92 | 0.00 | 0.00 | 10.48 | 0.00 | 0.00 | 0.00 | 0.00 | 0.00 |
| Scotland | 2018 | 50,317 | 0.37 | 0.05 | 0.00 | 0.14 | 0.01 | 0.00 | 0.00 | 0.00 | 0.03 | 204 | 167 | 17.16 | 0.00 | 0.00 | 16.67 | 0.00 | 0.00 | 0.98 | 0.00 | 0.00 |
| Scotland | 2019 | 48,609 | 0.11 | 0.02 | 0.00 | 0.01 | 0.01 | 0.00 | 0.01 | 0.00 | 0.02 | 184 | 154 | 12.50 | 0.00 | 0.00 | 13.59 | 0.00 | 0.00 | 2.17 | 0.00 | 0.00 |
| Scotland | 2020 | 46,642 | 0.08 | 0.04 | 0.00 | 0.01 | 0.01 | 0.00 | 0.00 | 0.00 | 0.03 | 190 | 168 | 5.26 | 0.00 | 0.00 | 5.26 | 0.00 | 0.00 | 1.05 | 0.00 | 0.00 |
| Sweden | 2008 | 105,724 | 3.84 | 0.00 | 0.92 | 0.10 | 0.00 | 7.12 | 0.12 | 0.00 | 5.83 | 382 | 217 | 2.88 | 0.00 | 0.00 | 0.00 | 5.24 | 2.62 | 1.31 | 0.00 | 10.21 |
| Sweden | 2009 | 107,171 | 0.00 | 0.00 | 0.00 | 0.00 | 0.00 | 0.00 | 0.00 | 0.00 | 0.00 | 438 | 254 | 2.74 | 0.00 | 0.00 | 0.00 | 11.87 | 2.05 | 3.42 | 0.00 | 11.19 |
| Sweden | 2010 | 112,571 | 0.00 | 0.00 | 0.00 | 0.00 | 0.00 | 0.00 | 0.00 | 0.00 | 0.00 | 420 | 235 | 4.05 | 0.00 | 0.00 | 0.00 | 12.38 | 0.00 | 2.14 | 0.00 | 9.05 |
| Sweden | 2011 | 109,002 | 0.00 | 0.00 | 0.00 | 0.00 | 0.00 | 0.00 | 0.00 | 0.00 | 0.00 | 432 | 263 | 3.70 | 0.00 | 0.00 | 0.00 | 11.81 | 0.00 | 3.24 | 0.00 | 10.42 |
| Sweden | 2012 | 109,908 | 0.00 | 0.00 | 0.00 | 0.00 | 0.00 | 0.00 | 0.00 | 0.00 | 0.00 | 428 | 272 | 3.04 | 0.00 | 0.00 | 0.00 | 10.51 | 1.87 | 2.10 | 0.00 | 8.88 |
| Sweden | 2013 | 110,383 | 0.00 | 0.00 | 0.00 | 0.00 | 0.00 | 0.00 | 0.00 | 0.00 | 0.00 | 416 | 239 | 3.85 | 0.00 | 0.00 | 0.00 | 10.10 | 1.92 | 2.16 | 0.00 | 10.82 |
| Sweden | 2014 | 112,856 | 0.00 | 0.00 | 0.00 | 0.00 | 0.00 | 0.00 | 0.00 | 0.00 | 0.00 | 450 | 293 | 2.67 | 0.00 | 0.00 | 0.00 | 10.67 | 1.56 | 2.22 | 0.00 | 9.11 |
| Sweden | 2015 | 113,267 | 0.00 | 0.00 | 0.00 | 0.00 | 0.00 | 0.00 | 0.00 | 0.00 | 0.00 | 418 | 246 | 5.74 | 0.00 | 0.00 | 0.00 | 10.05 | 3.11 | 2.87 | 0.00 | 13.88 |
| Sweden | 2016 | 115,944 | 0.00 | 0.00 | 0.00 | 0.00 | 0.00 | 0.00 | 0.00 | 0.00 | 0.00 | 417 | 248 | 2.88 | 0.00 | 0.00 | 1.92 | 11.03 | 1.92 | 4.56 | 0.00 | 11.27 |
| Sweden | 2017 | 113,963 | 0.00 | 0.00 | 0.00 | 0.00 | 0.00 | 0.00 | 0.00 | 0.00 | 0.00 | 414 | 253 | 1.69 | 0.00 | 0.00 | 1.21 | 8.45 | 3.14 | 4.35 | 0.00 | 10.63 |
| Sweden | 2018 | 114,630 | 0.00 | 0.00 | 0.00 | 0.00 | 0.00 | 0.00 | 0.00 | 0.00 | 0.00 | 435 | 248 | 3.22 | 0.00 | 0.00 | 1.38 | 9.20 | 2.53 | 2.99 | 0.00 | 10.57 |
| Sweden | 2019 | 113,343 | 0.00 | 0.00 | 0.00 | 0.00 | 0.00 | 0.00 | 0.00 | 0.00 | 0.00 | 363 | 210 | 4.13 | 0.00 | 0.00 | 0.00 | 7.71 | 0.00 | 3.58 | 0.00 | 11.29 |
| Uruguay | 2012 | 44,727 | 0.12 | 1.17 | 0.09 | 0.00 | 0.02 | 0.00 | 0.02 | 0.00 | 0.07 | 317 | 245 | 5.68 | 16.72 | 4.10 | 3.47 | 0.95 | 0.00 | 0.00 | 0.00 | 0.32 |
| Uruguay | 2013 | 45,900 | 0.21 | 0.96 | 0.16 | 0.00 | 0.05 | 0.01 | 0.02 | 0.00 | 0.05 | 352 | 259 | 3.41 | 21.88 | 3.13 | 3.13 | 1.70 | 0.00 | 0.28 | 0.00 | 1.14 |
| Uruguay | 2014 | 46,438 | 0.04 | 0.71 | 0.04 | 0.00 | 0.02 | 0.00 | 0.01 | 0.00 | 0.04 | 359 | 294 | 7.24 | 7.24 | 3.34 | 2.23 | 3.34 | 0.00 | 0.56 | 0.00 | 0.00 |
| Uruguay | 2015 | 46,475 | 0.19 | 0.85 | 0.15 | 0.00 | 0.00 | 0.00 | 0.00 | 0.00 | 0.00 | 336 | 284 | 3.87 | 10.42 | 1.49 | 1.79 | 2.08 | 0.00 | 0.00 | 0.00 | 0.00 |
| Uruguay | 2016 | 45,076 | 0.13 | 0.78 | 0.11 | 0.00 | 0.02 | 0.00 | 0.02 | 0.00 | 0.06 | 317 | 266 | 8.20 | 11.67 | 6.31 | 1.89 | 0.63 | 0.00 | 0.00 | 0.32 | 0.32 |
| Uruguay | 2017 | 41,609 | 0.07 | 0.58 | 0.05 | 0.00 | 0.02 | 0.00 | 0.02 | 0.00 | 0.02 | 336 | 268 | 9.23 | 15.18 | 6.55 | 1.19 | 1.49 | 0.00 | 0.00 | 0.00 | 0.00 |
| Uruguay | 2018 | 38,459 | 0.11 | 0.80 | 0.09 | 0.00 | 0.00 | 0.00 | 0.01 | 0.00 | 0.02 | 284 | 196 | 19.01 | 21.13 | 11.62 | 0.70 | 2.11 | 0.00 | 0.00 | 0.00 | 0.00 |
| Uruguay | 2019 | 35,341 | 0.11 | 0.89 | 0.08 | 0.00 | 0.00 | 0.00 | 0.00 | 0.00 | 0.01 | 252 | 165 | 23.02 | 19.44 | 10.71 | 0.40 | 1.98 | 0.00 | 0.00 | 0.00 | 0.40 |
| Uruguay | 2020 | 34,543 | 0.15 | 1.00 | 0.14 | 0.00 | 0.03 | 0.00 | 0.01 | 0.00 | 0.02 | 233 | 152 | 16.31 | 27.47 | 11.59 | 2.15 | 1.29 | 0.00 | 0.00 | 0.00 | 0.00 |
| USA | 2000 | 4,007,957 | 0.12 | 1.06 | 0.05 | 0.00 | 0.11 | 0.13 | 0.01 | 0.00 | 0.04 | 27,046 | 17,665 | 11.12 | 3.42 | 1.18 | 4.64 | 22.03 | 0.06 | 6.32 | 0.03 | 0.38 |
| USA | 2001 | 3,979,543 | 0.08 | 0.98 | 0.02 | 0.00 | 0.11 | 0.13 | 0.01 | 0.00 | 0.04 | 26,408 | 17,477 | 10.50 | 3.27 | 1.26 | 4.18 | 21.62 | 0.05 | 6.71 | 0.02 | 0.29 |
| USA | 2002 | 3,974,227 | 0.08 | 1.02 | 0.02 | 0.00 | 0.11 | 0.13 | 0.01 | 0.00 | 0.03 | 25,980 | 17,072 | 10.49 | 2.99 | 1.10 | 4.17 | 22.01 | 0.07 | 6.85 | 0.02 | 0.30 |
| USA | 2003 | 4,039,360 | 0.09 | 1.08 | 0.01 | 0.00 | 0.11 | 0.12 | 0.01 | 0.00 | 0.04 | 25,683 | 17,018 | 9.68 | 1.37 | 0.00 | 3.99 | 22.79 | 0.07 | 6.62 | 0.01 | 0.26 |
| USA | 2004 | 4,062,714 | 0.09 | 1.06 | 0.01 | 0.00 | 0.11 | 0.12 | 0.01 | 0.00 | 0.03 | 25,706 | 17,158 | 10.18 | 1.35 | 0.00 | 4.04 | 22.52 | 0.07 | 5.94 | 0.03 | 0.32 |
| USA | 2005 | 4,102,847 | 0.10 | 0.72 | 0.01 | 0.00 | 0.12 | 0.11 | 0.01 | 0.00 | 0.03 | 25,931 | 16,817 | 11.88 | 2.52 | 1.04 | 4.25 | 22.77 | 0.05 | 6.32 | 0.02 | 0.22 |
| USA | 2006 | 4,234,499 | 0.11 | 0.61 | 0.02 | 0.00 | 0.11 | 0.09 | 0.01 | 0.00 | 0.03 | 26,002 | 17,268 | 11.97 | 1.95 | 0.50 | 4.55 | 22.24 | 0.06 | 4.56 | 0.00 | 0.33 |
| USA | 2007 | 4,307,092 | 0.11 | 0.16 | 0.01 | 0.00 | 0.12 | 0.01 | 0.01 | 0.00 | 0.03 | 26,632 | 17,533 | 12.44 | 1.03 | 0.40 | 4.81 | 23.79 | 0.02 | 4.77 | 0.02 | 0.27 |
| USA | 2008 | 4,240,158 | 0.10 | 0.13 | 0.02 | 0.00 | 0.12 | 0.01 | 0.01 | 0.00 | 0.03 | 26,367 | 17,600 | 13.05 | 0.71 | 0.28 | 4.55 | 23.25 | 0.03 | 4.19 | 0.02 | 0.30 |
| USA | 2009 | 4,124,198 | 0.10 | 0.12 | 0.02 | 0.00 | 0.11 | 0.01 | 0.01 | 0.00 | 0.03 | 24,902 | 16,588 | 9.38 | 0.58 | 0.16 | 4.67 | 23.44 | 0.01 | 8.00 | 0.02 | 0.33 |
| USA | 2010 | 3,993,492 | 0.10 | 0.13 | 0.01 | 0.00 | 0.11 | 0.01 | 0.01 | 0.00 | 0.03 | 24,276 | 16,151 | 9.42 | 0.70 | 0.22 | 4.72 | 23.33 | 0.01 | 7.97 | 0.02 | 0.26 |
| USA | 2011 | 3,947,847 | 0.12 | 0.12 | 0.04 | 0.00 | 0.11 | 0.01 | 0.01 | 0.00 | 0.05 | 24,319 | 16,193 | 9.54 | 0.58 | 0.20 | 4.91 | 23.76 | 0.02 | 7.71 | 0.00 | 0.27 |
| USA | 2012 | 3,947,771 | 0.10 | 0.10 | 0.02 | 0.00 | 0.11 | 0.01 | 0.01 | 0.00 | 0.05 | 24,108 | 15,924 | 9.32 | 0.68 | 0.27 | 4.87 | 23.42 | 0.02 | 8.80 | 0.02 | 0.23 |
| USA | 2013 | 3,927,847 | 0.11 | 0.09 | 0.02 | 0.00 | 0.11 | 0.01 | 0.01 | 0.00 | 0.04 | 23,621 | 15,854 | 9.11 | 0.52 | 0.15 | 4.74 | 22.98 | 0.00 | 8.43 | 0.01 | 0.19 |
| USA | 2014 | 3,987,017 | 0.08 | 0.08 | 0.02 | 0.00 | 0.11 | 0.01 | 0.01 | 0.00 | 0.04 | 24,032 | 16,246 | 7.66 | 0.58 | 0.17 | 3.42 | 21.83 | 0.02 | 8.33 | 0.01 | 0.17 |
| USA | 2015 | 3,977,767 | 0.09 | 0.07 | 0.02 | 0.00 | 0.10 | 0.00 | 0.01 | 0.00 | 0.04 | 23,811 | 16,256 | 7.28 | 0.45 | 0.12 | 3.16 | 21.90 | 0.01 | 8.37 | 0.01 | 0.18 |
| USA | 2016 | 3,944,206 | 0.12 | 0.09 | 0.03 | 0.00 | 0.10 | 0.00 | 0.01 | 0.00 | 0.04 | 23,921 | 16,263 | 6.84 | 0.51 | 0.13 | 3.14 | 22.45 | 0.01 | 8.44 | 0.02 | 0.17 |
| USA | 2017 | 3,855,137 | 0.08 | 0.07 | 0.02 | 0.00 | 0.10 | 0.00 | 0.01 | 0.00 | 0.03 | 22,860 | 15,637 | 7.20 | 0.58 | 0.11 | 3.39 | 21.83 | 0.01 | 8.73 | 0.00 | 0.14 |
| USA | 2018 | 3,792,323 | 0.08 | 0.07 | 0.02 | 0.00 | 0.10 | 0.00 | 0.01 | 0.00 | 0.03 | 22,540 | 15,338 | 7.06 | 0.54 | 0.15 | 3.17 | 22.30 | 0.01 | 9.07 | 0.01 | 0.16 |
| USA | 2019 | 3,747,984 | 0.10 | 0.07 | 0.03 | 0.00 | 0.10 | 0.00 | 0.01 | 0.00 | 0.03 | 21,556 | 14,724 | 6.90 | 0.55 | 0.18 | 3.33 | 22.34 | 0.00 | 9.25 | 0.00 | 0.15 |

# Table S4a. Maternal baseline characteristics in 15 countries with stillbirth data

| **Baseline characteristics** | | **Argentina** | **Australia** | **Denmark** | **England & Wales** | **Estonia** | **Iran** | **Lebanon** | **Malaysia** | **Mexico** | **The Netherlands** | **Qatar** | **Scotland** | **Sweden** | **Uruguay** | **USA** |
| --- | --- | --- | --- | --- | --- | --- | --- | --- | --- | --- | --- | --- | --- | --- | --- | --- |
| **Livebirth (n)** |  | **1,238,453** | **5,744,917** | **867,290** | **3,212,492** | **82,472** | **4,812,628** | **251,990** | **888,165** | **23,414,789** | **1,844,765** | **94,337** | **1,124,504** | **2,102,374** | **262,784** | **80,193,986** |
| **Sex of baby** | **Male** | 51.1 | 51.4 | 51.3 | 51.2 | 51.6 | 51.8 | 51.7 | 51.9 | 51.6 | 51.3 | 51.0 | 51.3 | 51.4 | 51.0 | 51.2 |
| **(%)** | **Female** | 48.9 | 48.6 | 48.7 | 48.8 | 48.4 | 48.2 | 48.3 | 48.1 | 48.4 | 48.7 | 49.0 | 48.7 | 48.6 | 49.0 | 48.8 |
| **Mother’s highest level of Education (%)** | **Primary Secondary** | 44.0 | 0.0 | 17.8 | 0.0 | 29.5 | 43.0 | 33.5 | 0.0 | 56.7 | 0.0 | 0.0 | 0.0 | 19.1 | 87.7 | 13.4 |
|  | **Upper secondary** | 37.0 | 0.0 | 42.9 | 0.0 | 22.9 | 34.3 | 25.0 | 0.0 | 27.2 | 0.0 | 0.0 | 0.0 | 40.1 | 5.7 | 39.7 |
|  | **Bachelor or above** | 14.2 | 0.0 | 32.9 | 0.0 | 47.5 | 21.7 | 30.0 | 0.0 | 14.7 | 0.0 | 0.0 | 0.0 | 39.9 | 6.6 | 21.8 |
|  | **Missing education** | 4.9 | 100.0 | 6.3 | 0.0 | 0.1 | 1.0 | 11.5 | 100.0 | 1.5 | 100.0 | 100.0 | 100.0 | 1.0 | 0.0 | 25.1 |
| **Age of mother (%)** | **<15y** | 0.4 | 0.0 | 0.0 | 0.0 | 0.0 | 0.1 | 0.0 | 0.1 | 0.8 | 0.0 | 0.01 | 0.0 | 0.0 | 0.5 | 2.7 |
|  | **15_19y** | 11.8 | 3.7 | 1.3 | 3.1 | 2.1 | 5.4 | 4.2 | 3.8 | 19.0 | 1.0 | 1.9 | 6.0 | 1.4 | 15.6 | 9.3 |
|  | **20_24y** | 23.4 | 13.8 | 10.8 | 14.6 | 11.5 | 18.4 | 19.4 | 17.7 | 29.6 | 9.0 | 15.3 | 17.2 | 12.2 | 23.8 | 22.9 |
|  | **25_29y** | 24.0 | 27.9 | 32.6 | 28.0 | 31.2 | 26.7 | 28.8 | 35.4 | 24.6 | 30.2 | 32.6 | 26.6 | 30.5 | 23.2 | 26.9 |
|  | **30_34y** | 20.9 | 33.3 | 35.8 | 31.8 | 32.6 | 27.7 | 24.5 | 26.7 | 16.3 | 38.4 | 31.0 | 30.1 | 34.7 | 21.6 | 23.8 |
|  | **35_39y** | 13.9 | 17.6 | 16.4 | 18.2 | 17.6 | 16.7 | 12.4 | 12.8 | 7.6 | 18.0 | 15.1 | 16.6 | 17.5 | 12.2 | 11.7 |
|  | **>=40y** | 4.1 | 3.7 | 3.1 | 4.3 | 5.0 | 5.0 | 3.4 | 3.5 | 1.9 | 3.1 | 3.9 | 3.5 | 3.8 | 3.0 | 2.7 |
|  | **Missing age** | 1.5 | 0.0 | 0.0 | 0.0 | 0.0 | 0.0 | 7.4 | 0.0 | 0.3 | 0.3 | 0.1 | 0.0 | 0.0 | 0.1 | 0.0 |
| **Facility Delivery (%)** | **Delivery outside facility** | 0.3 | 0.9 | 0.0 | 0.0 | 0.7 | 0.9 | 100.0 | 0.0 | 1.2 | 26.9 | 0.0 | 0.7 | 0.0 | 0.0 | 1.2 |
|  | **Delivery health facility** | 98.4 | 99.1 | 0.0 | 0.0 | 99.3 | 98.8 | 0.0 | 100.0 | 82.5 | 72.7 | 0.0 | 99.3 | 99.9 | 0.0 | 98.8 |
|  | **Missing place delivery** | 1.3 | 0.0 | 100.0 | 0.0 | 0.0 | 0.2 | 0.0 | 0.0 | 16.3 | 0.4 | 100.0 | 0.0 | 0.1 | 0.0 | 0.0 |
| **Mode of delivery (%)** | **Vaginal delivery** | 0.0 | 67.9 | 78.9 | 0.0 | 79.8 | 0.0 | 55.2 | 71.9 | 54.1 | 80.3 | 65.6 | 71.6 | 82.5 | 53.4 | 69.3 |
|  | **Caesarean section** | 0.0 | 32.1 | 21.1 | 0.0 | 20.2 | 0.0 | 38.6 | 28.4 | 45.6 | 16.6 | 34.1 | 28.4 | 17.5 | 44.5 | 30.5 |
|  | **Missing type delivery** | 100.0 | 0.1 | 0.0 | 100.0 | 0.0 | 100.0 | 6.1 | 0.1 | 0.3 | 3.1 | 0.3 | 0.0 | 0.0 | 2.1 | 0.1 |
| **Parity (%)** | **1** | 33.0 | 41.6 | 44.8 | 0.0 | 38.4 | 28.4 | 34.8 | 0.0 | 36.4 | 44.5 | 0.0 | 44.4 | 43.3 | 0.0 | 35.2 |
|  | **2** | 27.3 | 33.7 | 36.1 | 0.0 | 36.8 | 16.8 | 29.7 | 34.6 | 30.0 | 35.8 | 0.0 | 34.3 | 36.7 | 0.0 | 29.9 |
|  | **3** | 16.9 | 14.7 | 13.2 | 0.0 | 17.8 | 7.0 | 17.8 | 45.6 | 19.0 | 13.3 | 0.0 | 13.5 | 17.7 | 0.0 | 17.8 |
|  | **4** | 8.4 | 5.3 | 3.2 | 0.0 | 4.8 | 3.0 | 8.6 | 0.0 | 8.5 | 3.9 | 0.0 | 4.6 | 2.3 | 0.0 | 8.7 |
|  | **>=5** | 7.9 | 3.4 | 1.5 | 0.0 | 2.3 | 2.9 | 6.7 | 19.4 | 6.0 | 2.1 | 0.0 | 2.5 | 0.0 | 0.0 | 8.4 |
|  | **Missing parity** | 6.5 | 1.4 | 1.1 | 100.0 | 0.0 | 41.9 | 2.4 | 0.4 | 0.1 | 0.4 | 100.0 | 0.8 | 0.0 | 100.0 | 0.0 |
| **Singleton or multiple birth (%)** | **Singleton** | 97.8 | 96.9 | 95.8 | 97.0 | 98.4 | 96.7 | 94.0 | 97.9 | 98.2 | 96.8 | 94.5 | 97.0 | 97.2 | 97.1 | 96.6 |
|  | **Twins** | 2.0 | 3.0 | 4.2 | 2.9 | 1.6 | 3.0 | 3.7 | 2.1 | 1.4 | 3.1 | 3.9 | 2.9 | 2.8 | 1.5 | 3.2 |
|  | **Triplets higher** | 0.0 | 0.1 | 0.1 | 0.1 | 0.0 | 0.2 | 0.4 | 0.1 | 0.0 | 0.1 | 0.4 | 0.1 | 0.1 | 1.3 | 0.1 |
|  | **Missing multiple** | 0.2 | 0.0 | 0.0 | 0.0 | 0.0 | 0.1 | 1.9 | 0.0 | 0.3 | 0.0 | 1.2 | 0.0 | 0.0 | 0.0 | 0.0 |
| **Gestational Age Assessment (%)** | **Ultrasound Before 14week** | 0.0 | 0.0 | 0.0 | 0.0 | 0.0 | 0.0 | 0.0 | 0.0 | 0.0 | 0.0 | 0.0 | 0.0 | 0.0 | 0.0 | 0.0 |
|  | **Ultrasound_14_24week** | 0.0 | 0.0 | 0.0 | 0.0 | 83.3 | 0.0 | 0.0 | 0.0 | 0.0 | 0.0 | 0.0 | 0.0 | 100.0 | 0.0 | 95.5 |
|  | **Ultrasound After 24week** | 0.0 | 0.0 | 0.0 | 0.0 | 0.0 | 0.0 | 0.0 | 0.0 | 0.0 | 0.0 | 0.0 | 0.0 | 0.0 | 0.0 | 4.3 |
|  | **Best Obstetric estimate** | 0.0 | 0.0 | 0.0 | 0.0 | 14.5 | 0.0 | 0.0 | 0.0 | 0.0 | 0.0 | 0.0 | 100.0 | 0.0 | 0.0 | 0.2 |
|  | **Last menstrual period** | 0.0 | 0.0 | 0.0 | 0.0 | 1.6 | 0.0 | 0.0 | 0.0 | 0.0 | 0.0 | 0.0 | 0.0 | 0.0 | 0.0 | 0.0 |
|  | **Symphysis Fundal Height** | 0.0 | 0.0 | 100.0 | 0.0 | 0.0 | 0.0 | 0.0 | 0.0 | 0.0 | 0.0 | 0.0 | 0.0 | 0.0 | 0.0 | 0.0 |
|  | **Method not stated** | 100.0 | 100.0 | 0.0 | 100.0 | 98.4 | 100.0 | 100.0 | 100.0 | 100.0 | 100.0 | 100.0 | 0.0 | 100.0 | 100.0 | 98.8 |
|  | **Ultrasound unknown** | 0.0 | 0.0 | 0.0 | 0.0 | 0.7 | 0.0 | 100.0 | 0.0 | 100.0 | 0.0 | 0.0 | 0.0 | 0.0 | 0.0 | 0.0 |

# Table S4b. Maternal baseline characteristics in 15 countries with stillbirth data

| **Baseline characteristics** | | **Argentina** | **Australia** | **Denmark** | **England & Wales** | **Estonia** | **Iran** | **Lebanon** | **Malaysia** | **Mexico** | **The Netherlands** | **Qatar** | **Scotland** | **Sweden** | **Uruguay** | **USA** |
| --- | --- | --- | --- | --- | --- | --- | --- | --- | --- | --- | --- | --- | --- | --- | --- | --- |
| **Stillbirth (n)** |  | **9,297** | **28,528** | **2,991** | **13,826** | **244** | **38,462** | **467** | **6,725** | **185,380** | **8,997** | **509** | **5,127** | **4,664** | **1,405** | **330,782** |
| **Sex of baby (%)** | **Male** | 54.3 | 52.1 | 50.1 | 51.8 | 52.0 | 52.9 | 55.2 | 53.8 | 53.7 | 51.9 | 56.8 | 51.1 | 50.2 | 54.2 | 52.0 |
|  | **Female** | 45.7 | 47.9 | 49.9 | 48.2 | 48.0 | 47.1 | 44.8 | 46.2 | 46.3 | 48.1 | 43.2 | 48.9 | 49.8 | 45.8 | 48.0 |
| **Mother’s highest level of Education (%)** | **Primary Secondary** | 48.6 | 0.0 | 26.4 | 0.0 | 40.2 | 52.3 | 41.5 | 0.0 | 69.5 | 0.0 | 0.0 | 0.0 | 27.1 | 1.5 | 8.6 |
|  | **Upper secondary** | 29.1 | 0.0 | 41.8 | 0.0 | 24.6 | 31.4 | 14.3 | 0.0 | 20.0 | 0.0 | 0.0 | 0.0 | 40.1 | 4.4 | 24.2 |
|  | **Bachelor or above** | 6.7 | 0.0 | 25.1 | 0.0 | 35.2 | 14.9 | 12.4 | 0.0 | 9.1 | 0.0 | 0.0 | 0.0 | 31.9 | 6.4 | 9.0 |
|  | **Missing education** | 15.5 | 100.0 | 6.7 | 0.0 | 0.0 | 1.3 | 31.8 | 0.0 | 1.4 | 100.0 | 100.0 | 100.0 | 0.9 | 87.8 | 58.1 |
| **Age of mother (%)** | **<15y** | 0.6 | 0.0 | 0.0 | 4.0 | 0.0 | 0.2 | 0.1 | 0.1 | 0.6 | 0.0 | 0.2 | 0.0 | 0.0 | 0.6 | 2.9 |
|  | **15_19y** | 13.6 | 6.8 | 2.0 | 16.2 | 2.5 | 6.1 | 5.5 | 4.4 | 16.0 | 1.5 | 2.0 | 7.6 | 1.2 | 16.0 | 10.6 |
|  | **20_24y** | 21.9 | 16.1 | 12.3 | 25.8 | 16.0 | 16.9 | 20.1 | 15.9 | 23.6 | 10.2 | 17.3 | 18.4 | 12.3 | 22.3 | 23.1 |
|  | **25_29y** | 20.4 | 24.8 | 27.8 | 27.7 | 27.5 | 24.3 | 23.8 | 29.8 | 20.7 | 28.0 | 27.9 | 23.9 | 27.0 | 22.3 | 24.6 |
|  | **30_34y** | 16.8 | 28.5 | 34.5 | 19.0 | 29.1 | 26.2 | 22.5 | 25.7 | 16.7 | 34.8 | 29.3 | 27.1 | 32.9 | 19.3 | 21.2 |
|  | **35_39y** | 13.1 | 17.9 | 18.1 | 6.0 | 13.1 | 18.8 | 17.8 | 16.6 | 10.9 | 19.5 | 16.5 | 17.8 | 20.6 | 14.0 | 13.0 |
|  | **>=40y** | 5.8 | 5.7 | 4.4 | 1.2 | 11.9 | 7.5 | 7.3 | 7.5 | 3.9 | 5.3 | 6.9 | 5.2 | 6.0 | 4.5 | 4.6 |
|  | **Missing age** | 7.9 | 0.2 | 0.9 | 0.0 | 0.0 | 0.0 | 2.9 | 0.0 | 7.6 | 0.7 | 0.0 | 0.0 | 0.0 | 1.0 | 0.0 |
| **Facility Delivery (%)** | **Delivery outside facility** | 0.0 | 0.0 | 0.0 | 0.0 | 0.8 | 1.0 | 100.0 | 0.0 | 4.1 | 1.3 | 0.0 | 1.7 | 0.0 | 0.0 | 1.5 |
|  | **Delivery health facility** | 0.0 | 1.3 | 100.0 | 0.0 | 99.2 | 98.7 | 0.0 | 100.0 | 94.4 | 98.5 | 0.0 | 98.3 | 99.9 | 0.0 | 98.4 |
|  | **Missing place delivery** | 100.0 | 98.7 | 0.0 | 0.0 | 0.0 | 0.2 | 0.0 | 0.0 | 1.5 | 0.3 | 100.0 | 0.0 | 0.1 | 100.0 | 0.1 |
| **Mode of delivery (%)** | **Vaginal delivery** | 0.0 | 68.8 | 86.5 | 0.0 | 83.2 | 76.8 | 73.4 | 85.0 | 54.8 | 87.4 | 80.0 | 82.6 | 86.0 | 50.0 | 79.2 |
|  | **Caesarean section** | 0.0 | 28.4 | 13.5 | 0.0 | 16.8 | 22.6 | 23.2 | 15.0 | 27.1 | 5.2 | 17.5 | 16.8 | 14.0 | 44.0 | 18.0 |
|  | **Missing type delivery** | 100.0 | 2.9 | 0.0 | 0.0 | 0.0 | 0.6 | 3.4 | 0.1 | 18.1 | 7.4 | 2.6 | 0.5 | 0.0 | 6.5 | 2.8 |
| **Parity (%)** | **1** | 28.3 | 22.5 | 46.5 | 0.0 | 37.3 | 28.4 | 37.8 | 36.9 | 25.1 | 49.4 | 0.0 | 47.8 | 46.8 | 0.0 | 33.6 |
|  | **2** | 14.8 | 17.9 | 24.8 | 0.0 | 35.2 | 16.8 | 22.5 | 23.5 | 26.7 | 29.4 | 0.0 | 27.3 | 30.0 | 0.0 | 22.9 |
|  | **3** | 10.6 | 8.4 | 12.8 | 0.0 | 15.2 | 7.0 | 16.6 | 16.2 | 16.0 | 12.8 | 0.0 | 14.7 | 19.3 | 0.0 | 16.4 |
|  | **4** | 6.1 | 3.8 | 4.4 | 0.0 | 6.1 | 3.0 | 8.7 | 10.2 | 6.8 | 5.0 | 0.0 | 5.5 | 3.8 | 0.0 | 9.8 |
|  | **>=5** | 7.8 | 3.5 | 2.7 | 0.0 | 6.1 | 3.0 | 13.2 | 12.8 | 25.4 | 3.4 | 0.0 | 3.8 | 0.0 | 0.0 | 16.6 |
|  | **Missing parity** | 32.4 | 43.9 | 8.7 | 0.0 | 0.0 | 41.8 | 1.2 | 0.4 | 0.0 | 0.0 | 100.0 | 0.8 | 0.0 | 100.0 | 0.7 |
| **Singleton or multiple birth (%)** | **Singleton** | 81.8 | 75.1 | 93.0 | 93.5 | 93.4 | 87.2 | 86.3 | 93.8 | 94.3 | 92.7 | 86.2 | 90.5 | 95.5 | 93.6 | 93.0 |
|  | **Twins** | 3.6 | 22.8 | 6.3 | 6.1 | 6.6 | 6.5 | 7.4 | 5.9 | 5.4 | 6.9 | 12.4 | 9.3 | 4.5 | 3.5 | 6.5 |
|  | **Triplets higher** | 0.0 | 2.0 | 0.7 | 0.4 | 0.0 | 0.8 | 2.6 | 0.3 | 0.3 | 0.4 | 1.4 | 0.2 | 0.0 | 2.9 | 0.5 |
|  | **Missing multiple** | 14.6 | 0.1 | 0.0 | 0.0 | 0.0 | 5.6 | 3.8 | 0.0 | 0.0 | 0.0 | 0.0 | 0.1 | 0.0 | 0.0 | 0.0 |
| **Gestational Age Assessment (%)** | **Ultrasound Before 14week** | 0.0 | 0.0 | 0.0 | 0.0 | 4.5 | 0.0 | 0.0 | 0.0 | 0.0 | 0.0 | 0.0 | 0.0 | 0.0 | 0.0 | 0.0 |
|  | **Ultrasound_14_24week** | 0.0 | 0.0 | 0.0 | 0.0 | 84.0 | 0.0 | 0.0 | 0.0 | 0.0 | 0.0 | 0.0 | 0.0 | 0.0 | 0.0 | 94.9 |
|  | **Ultrasound After 24week** | 0.0 | 0.0 | 0.0 | 0.0 | 0.0 | 0.0 | 0.0 | 0.0 | 0.0 | 0.0 | 0.0 | 0.0 | 100.0 | 0.0 | 4.9 |
|  | **Best Obstetric estimate** | 0.0 | 0.0 | 0.0 | 0.0 | 8.6 | 0.0 | 0.0 | 0.0 | 0.0 | 0.0 | 0.0 | 100.0 | 0.0 | 0.0 | 0.2 |
|  | **Last menstrual period** | 0.0 | 0.0 | 0.0 | 0.0 | 2.9 | 0.0 | 0.0 | 0.0 | 0.0 | 0.0 | 0.0 | 0.0 | 0.0 | 0.0 | 0.0 |
|  | **Symphysis Fundal Height** | 0.0 | 0.0 | 100.0 | 0.0 | 0.0 | 0.0 | 0.0 | 0.0 | 0.0 | 0.0 | 0.0 | 0.0 | 0.0 | 0.0 | 0.0 |
|  | **Method not stated** | 100.0 | 100.0 | 0.0 | 0.0 | 0.0 | 100.0 | 100.0 | 100.0 | 100.0 | 100.0 | 100.0 | 0.0 | 0.0 | 100.0 | 0.0 |
|  | **Ultrasound unknown** | 100.0 | 100.0 | 0.0 | 0.0 | 0.0 | 0.0 | 100.0 | 0.0 | 100.0 | 100.0 | 0.0 | 0.0 | 0.0 | 0.0 | 0.0 |

# Figure S1a. Stillbirth rate by gestational week for SGA, AGA and LGA pregnancies using birth based approach


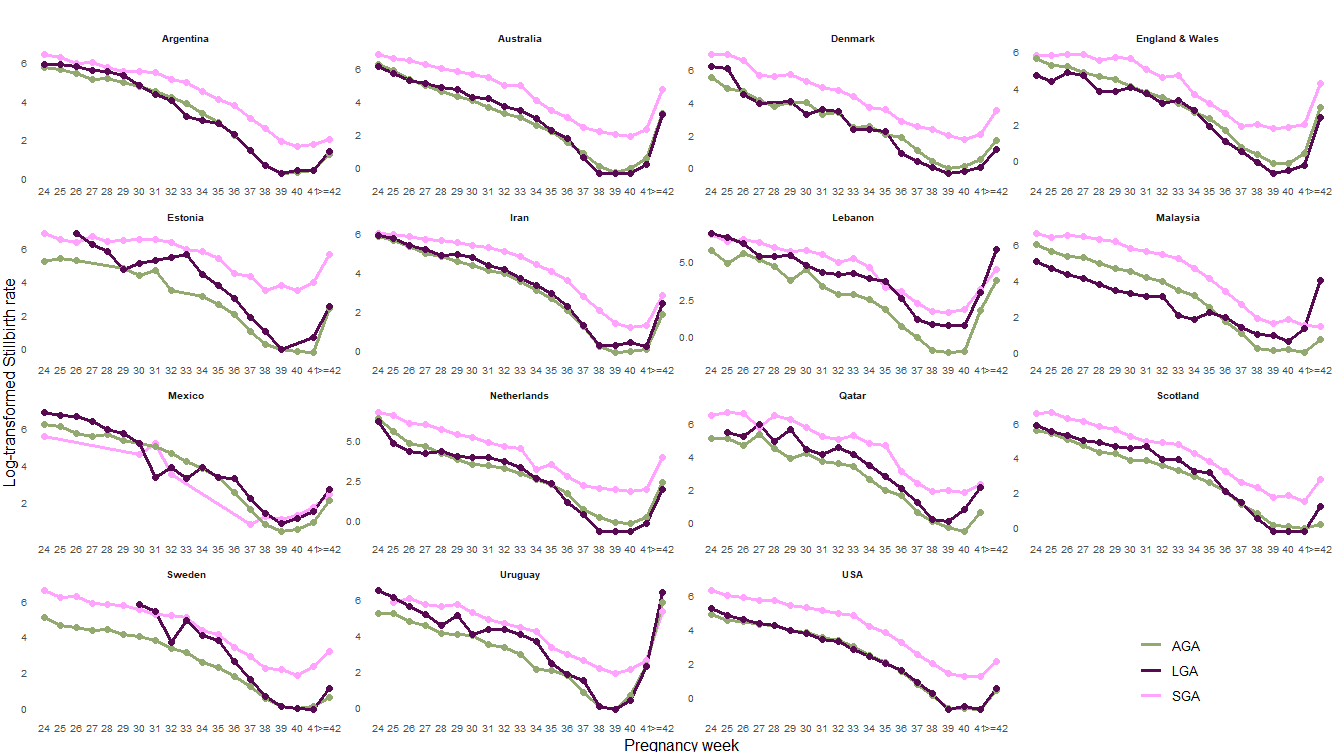


# Figure S1b. Stillbirth rate by gestational week for SGA, AGA and LGA pregnancies using fetuses-at-risk approach.


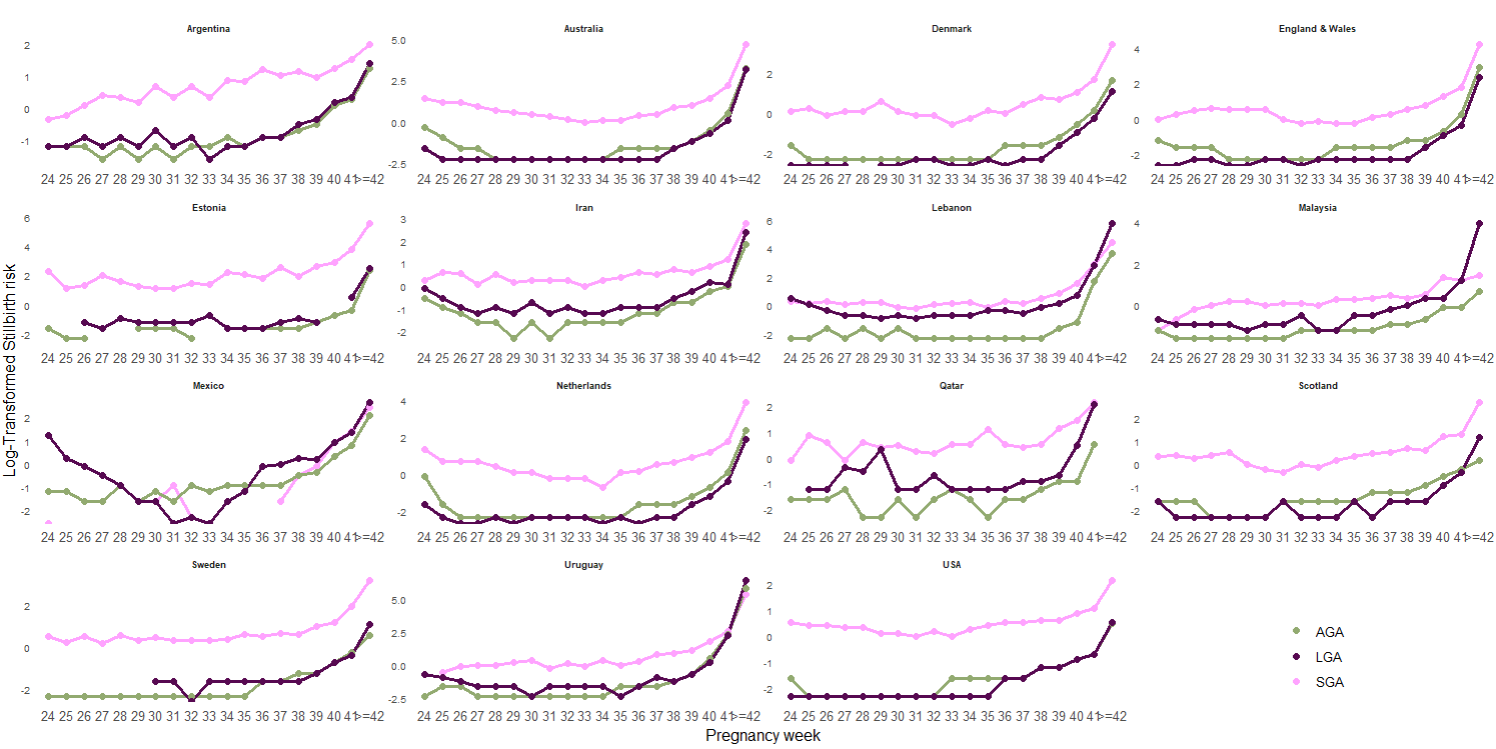


# Table S5. Stillbirth rate per 1000 total birth and stillbirth risk per 1,000 fetuses- still in utero

|  |  |  |  | **Stillbirth overall distribution of size for gestational age** | | | | | | **Birth-based approach** | | | **Fetuses-At-RIsk approach** | | |
| --- | --- | --- | --- | --- | --- | --- | --- | --- | --- | --- | --- | --- | --- | --- | --- |
| **Country** | **Week** | **Livebirth** | **Stillbirth** | **SGA (n)** | **AGA (n)** | **LGA (n)** | **SGA % (95%CI)** | **AGA % (95%CI)** | **LGA % (95%CI)** | **SGA Stillbirth rate per 1,000 total births ( 95%CI)** | **AGA Stillbirth rate per 1,000 total births ( 95%CI)** | **LGA Stillbirth rate per 1,000 total births ( 95%CI)** | **SGA Stillbirths risk per 1,000 ongoing pregnancies  ( 95%CI)** | **AGA Stillbirths risk per 1,000 ongoing pregnancies  ( 95%CI)** | **LGA Stillbirths risk per 1,000 ongoing pregnancies  ( 95%CI)** |
| Argentina | 24 | 755 | 399 | 50 | 278 | 71 | 12.3   ( 11.8 , 12.7 ) | 69.74   ( 69.6 , 69.8 ) | 17.8   ( 17.7 , 17.8 ) | 606.3   ( 606.3 , 606.4 ) | 316.76   ( 316.7 , 316.8 ) | 365.6   ( 365.5 , 365.6 ) | 0.7   ( 0.2 , 1.2 ) | 0.3   ( 0.3 , 0.3 ) | 0.3   ( 0.1 , 0.5 ) |
| Argentina | 25 | 809 | 369 | 55 | 242 | 72 | 14.8   ( 14.6 , 15 ) | 65.59   ( 65.5 , 65.7 ) | 19.4   ( 19.3 , 19.6 ) | 522.2   ( 522.1 , 522.4 ) | 275.22   ( 275.1 , 275.3 ) | 374.4   ( 374.3 , 374.5 ) | 0.8   ( 0.6 , 0.9 ) | 0.3   ( 0.2 , 0.3 ) | 0.3   ( 0.1 , 0.5 ) |
| Argentina | 26 | 1174 | 421 | 78 | 256 | 87 | 18.3   ( 18.1 , 18.6 ) | 60.9   ( 60.8 , 61 ) | 20.6   ( 20.6 , 20.7 ) | 382.6   ( 382.5 , 382.7 ) | 226.55   ( 226.4 , 226.7 ) | 331.1   ( 330.8 , 331.4 ) | 1.1   ( 0.7 , 1.4 ) | 0.3   ( 0.2 , 0.3 ) | 0.4   ( 0.2 , 0.5 ) |
| Argentina | 27 | 1305 | 373 | 104 | 192 | 77 | 27.8   ( 27.6 , 27.9 ) | 51.51   ( 51.4 , 51.6 ) | 20.6   ( 20.6 , 20.6 ) | 411.9   ( 411.8 , 411.9 ) | 169.42   ( 169.3 , 169.5 ) | 265.5   ( 265.4 , 265.7 ) | 1.5   ( 1.1 , 1.8 ) | 0.2   ( 0.1 , 0.3 ) | 0.3   ( 0.2 , 0.5 ) |
| Argentina | 28 | 1883 | 493 | 101 | 282 | 110 | 20.5   ( 20.4 , 20.6 ) | 57.06   ( 56.9 , 57.2 ) | 22   ( 21.5 , 22.5 ) | 310.4   ( 310.1 , 310.8 ) | 174.16   ( 174.1 , 174.3 ) | 250.1   ( 249.6 , 250.5 ) | 1.4   ( 1.2 , 1.6 ) | 0.3   ( 0.1 , 0.5 ) | 0.4   ( 0 , 0.9 ) |
| Argentina | 29 | 1815 | 378 | 88 | 212 | 78 | 22.5   ( 21.8 , 23.2 ) | 56.08   ( 56 , 56.1 ) | 20.2   ( 19.6 , 20.8 ) | 253.2   ( 252.8 , 253.7 ) | 142.75   ( 142.7 , 142.8 ) | 206.7   ( 206.3 , 207.1 ) | 1.2   ( 0.4 , 2 ) | 0.2   ( 0.2 , 0.2 ) | 0.3   ( -0.2 , 0.9 ) |
| Argentina | 30 | 3395 | 534 | 140 | 278 | 116 | 26.2   ( 26.2 , 26.3 ) | 52.04   ( 52 , 52.1 ) | 21.7   ( 21.6 , 21.8 ) | 255.7   ( 255.6 , 255.8 ) | 113.61   ( 113.5 , 113.7 ) | 123.9   ( 123.8 , 124 ) | 2   ( 1.9 , 2.1 ) | 0.3   ( 0.3 , 0.3 ) | 0.5   ( 0.3 , 0.7 ) |
| Argentina | 31 | 3377 | 387 | 99 | 217 | 71 | 25.6   ( 25.5 , 25.6 ) | 55.94   ( 55.7 , 56.1 ) | 17.8   ( 17.1 , 18.5 ) | 240.4   ( 240.3 , 240.5 ) | 88.36   ( 88.2 , 88.5 ) | 76.4   ( 75.7 , 77.1 ) | 1.4   ( 1.3 , 1.5 ) | 0.2   ( 0 , 0.5 ) | 0.3   ( -0.4 , 0.9 ) |
| Argentina | 32 | 6587 | 541 | 139 | 310 | 92 | 25.2   ( 24.6 , 25.8 ) | 57.12   ( 57 , 57.3 ) | 16.8   ( 16.3 , 17.2 ) | 166.1   ( 165.8 , 166.4 ) | 65.31   ( 65.1 , 65.5 ) | 57.7   ( 57 , 58.3 ) | 2   ( 1.5 , 2.4 ) | 0.3   ( 0.1 , 0.6 ) | 0.4   ( -0.1 , 0.9 ) |
| Argentina | 33 | 7814 | 430 | 99 | 290 | 41 | 22.8   ( 22.4 , 23.1 ) | 67.45   ( 67.4 , 67.5 ) | 9.4   ( 8.9 , 9.9 ) | 145.9   ( 145.7 , 146.1 ) | 48.63   ( 48.6 , 48.7 ) | 25.2   ( 24.5 , 25.8 ) | 1.4   ( 1.1 , 1.8 ) | 0.3   ( 0.3 , 0.4 ) | 0.2   ( -0.3 , 0.7 ) |
| Argentina | 34 | 15761 | 554 | 162 | 331 | 61 | 29.2   ( 29 , 29.3 ) | 59.74   ( 59.6 , 59.9 ) | 10.9   ( 10.5 , 11.2 ) | 91.1   ( 90.9 , 91.2 ) | 28.75   ( 28.7 , 28.8 ) | 20   ( 19.7 , 20.2 ) | 2.4   ( 2.3 , 2.5 ) | 0.4   ( 0.2 , 0.5 ) | 0.3   ( -0.1 , 0.6 ) |
| Argentina | 35 | 23124 | 528 | 155 | 306 | 67 | 29.2   ( 28.9 , 29.5 ) | 57.94   ( 57.9 , 58 ) | 12.6   ( 12.2 , 12.9 ) | 60.3   ( 60 , 60.6 ) | 17.75   ( 17.7 , 17.8 ) | 17.3   ( 17 , 17.5 ) | 2.3   ( 2 , 2.7 ) | 0.3   ( 0.3 , 0.4 ) | 0.3   ( -0.1 , 0.6 ) |
| Argentina | 36 | 50781 | 641 | 206 | 351 | 84 | 32.1   ( 32 , 32.3 ) | 54.68   ( 54.6 , 54.8 ) | 13.1   ( 13 , 13.2 ) | 44.1   ( 44 , 44.2 ) | 9.22   ( 9.1 , 9.4 ) | 9.6   ( 9.4 , 9.8 ) | 3.3   ( 3.1 , 3.4 ) | 0.4   ( 0.3 , 0.5 ) | 0.4   ( 0.3 , 0.5 ) |
| Argentina | 37 | 117583 | 632 | 166 | 368 | 98 | 26.3   ( 26.1 , 26.4 ) | 58.18   ( 58.1 , 58.2 ) | 15.5   ( 15.5 , 15.5 ) | 22.6   ( 22.5 , 22.7 ) | 4.16   ( 4 , 4.3 ) | 4.3   ( 4.2 , 4.5 ) | 2.8   ( 2.7 , 3 ) | 0.4   ( 0.3 , 0.5 ) | 0.4   ( 0.4 , 0.5 ) |
| Argentina | 38 | 286767 | 684 | 161 | 404 | 119 | 23.5   ( 23.3 , 23.7 ) | 59.06   ( 59.1 , 59.1 ) | 17.3   ( 17.1 , 17.6 ) | 13.5   ( 13.3 , 13.8 ) | 1.89   ( 1.9 , 1.9 ) | 1.9   ( 1.6 , 2.2 ) | 3.1   ( 2.8 , 3.5 ) | 0.5   ( 0.5 , 0.6 ) | 0.6   ( 0.4 , 0.8 ) |
| Argentina | 39 | 338195 | 516 | 102 | 325 | 89 | 19.7   ( 19.5 , 19.9 ) | 62.99   ( 63 , 63 ) | 17.2   ( 17.1 , 17.4 ) | 6.7   ( 6.5 , 6.9 ) | 1.27   ( 1.2 , 1.3 ) | 1.3   ( 1.2 , 1.4 ) | 2.6   ( 2.5 , 2.7 ) | 0.6   ( 0.5 , 0.7 ) | 0.7   ( 0.5 , 0.8 ) |
| Argentina | 40 | 292454 | 468 | 85 | 303 | 80 | 18.2   ( 18 , 18.3 ) | 64.57   ( 64.4 , 64.7 ) | 17   ( 16.6 , 17.4 ) | 5.1   ( 5.1 , 5.1 ) | 1.36   ( 1.1 , 1.6 ) | 1.5   ( 1.2 , 1.8 ) | 3.5   ( 3.5 , 3.6 ) | 1.1   ( 0.8 , 1.4 ) | 1.2   ( 0.9 , 1.4 ) |
| Argentina | 41 | 73952 | 140 | 33 | 85 | 22 | 23.6   ( 23.4 , 23.8 ) | 60.78   ( 60.5 , 61 ) | 13.2   ( 11.7 , 14.7 ) | 5.9   ( 5.6 , 6.2 ) | 1.52   ( 1.3 , 1.8 ) | 1.5   ( -0.1 , 3 ) | 4.6   ( 4.2 , 4.9 ) | 1.3   ( 1.1 , 1.6 ) | 1.4   ( -0.2 , 2.9 ) |
| Argentina | >=42 | 10270 | 44 | 13 | 27 | 4 | 28   ( 27.5 , 28.5 ) | 61.92   ( 61.8 , 62 ) | 9.3   ( 8.7 , 10 ) | 7.3   ( 6.6 , 7.9 ) | 3.51   ( 3.4 , 3.6 ) | 4.1   ( 3.7 , 4.4 ) | 7.3   ( 6.6 , 7.9 ) | 3.5   ( 3.4 , 3.6 ) | 4.1   ( 3.7 , 4.4 ) |

| Australia | 24 | 3316 | 3964 | 485 | 3252 | 227 | 26.1   ( 25.1 , 27 ) | 77.69   ( 77.2 , 78.2 ) | 5.7   ( 5 , 6.5 ) | 903.6   ( 903.4 , 903.7 ) | 489   ( 488.4 , 489.6 ) | 439.8   ( 439.3 , 440.2 ) | 4.2   ( 3.6 , 4.7 ) | 0.7   ( -0.2 , 1.6 ) | 0.2   ( -0.5 , 0.9 ) |
| --- | --- | --- | --- | --- | --- | --- | --- | --- | --- | --- | --- | --- | --- | --- | --- |
| Australia | 25 | 3836 | 2865 | 824 | 1872 | 169 | 27.8   ( 26.9 , 28.6 ) | 62.22   ( 61.8 , 62.7 ) | 6.4   ( 5.8 , 7.1 ) | 718.5   ( 718.2 , 718.7 ) | 345.17   ( 344.6 , 345.8 ) | 292.6   ( 292.1 , 293 ) | 3.3   ( 2.5 , 4.1 ) | 0.4   ( -0.4 , 1.2 ) | 0.1   ( -0.4 , 0.7 ) |
| Australia | 26 | 4894 | 1867 | 754 | 1019 | 94 | 39.4   ( 38.9 , 39.9 ) | 53.17   ( 52.8 , 53.6 ) | 7.8   ( 7.3 , 8.3 ) | 632.9   ( 632.7 , 633.1 ) | 200.68   ( 200.2 , 201.2 ) | 192.9   ( 192.5 , 193.2 ) | 3.2   ( 2.7 , 3.7 ) | 0.2   ( -0.3 , 0.8 ) | 0.1   ( -0.3 , 0.5 ) |
| Australia | 27 | 5557 | 1456 | 582 | 758 | 116 | 39.7   ( 39.2 , 40.1 ) | 50.98   ( 50.7 , 51.3 ) | 9.8   ( 9.2 , 10.5 ) | 512.7   ( 512.4 , 513 ) | 145.51   ( 145 , 146 ) | 163.1   ( 162.5 , 163.8 ) | 2.5   ( 2.1 , 2.9 ) | 0.2   ( -0.3 , 0.6 ) | 0.1   ( -0.5 , 0.8 ) |
| Australia | 28 | 7144 | 1200 | 464 | 631 | 105 | 38.6   ( 38.1 , 39.1 ) | 51.53   ( 51.2 , 51.8 ) | 11   ( 10.2 , 11.7 ) | 395.4   ( 395.1 , 395.7 ) | 99.87   99.4 , 100.4 ) | 127.4   ( 126.7 , 128.1 ) | 2   ( 1.6 , 2.4 ) | 0.1   ( -0.3 , 0.6 ) | 0.1   ( -0.6 , 0.8 ) |
| Australia | 29 | 8232 | 1020 | 410 | 531 | 79 | 40   ( 39.5 , 40.6 ) | 50.6   ( 50.3 , 50.9 ) | 11.9   ( 11.5 , 12.3 ) | 334.9   ( 334.6 , 335.3 ) | 73.54   ( 73 , 74.1 ) | 111.6   ( 111 , 112.2 ) | 1.8   ( 1.4 , 2.2 ) | 0.1   ( -0.5 , 0.7 ) | 0.1   ( -0.4 , 0.6 ) |
| Australia | 30 | 11188 | 966 | 374 | 518 | 74 | 37.9   ( 37.6 , 38.3 ) | 53.36   ( 53 , 53.7 ) | 12   ( 11.6 , 12.4 ) | 276.4   ( 276 , 276.8 ) | 55.6   ( 55.3 , 55.9 ) | 70.5   ( 70.1 , 71 ) | 1.6   ( 1.2 , 2.1 ) | 0.1   ( -0.2 , 0.4 ) | 0.1   ( -0.3 , 0.5 ) |
| Australia | 31 | 14908 | 856 | 323 | 489 | 44 | 38   ( 37.7 , 38.3 ) | 56.34   ( 56 , 56.7 ) | 13   ( 12.7 , 13.3 ) | 230   ( 229.6 , 230.4 ) | 37.99   ( 37.5 , 38.5 ) | 66.2   ( 65.9 , 66.6 ) | 1.4   ( 1 , 1.9 ) | 0.1   ( -0.4 , 0.6 ) | 0.1   ( -0.1 , 0.3 ) |
| Australia | 32 | 22876 | 819 | 269 | 526 | 24 | 32.7   ( 32.3 , 33.1 ) | 63.55   ( 63.3 , 63.8 ) | 12.7   ( 12.1 , 13.4 ) | 144.6   ( 144.1 , 145.1 ) | 26.76   ( 26.3 , 27.2 ) | 40   ( 39.5 , 40.4 ) | 1.2   ( 0.9 , 1.5 ) | 0.1   ( -0.3 , 0.6 ) | 0.1   ( -0.4 , 0.5 ) |
| Australia | 33 | 32498 | 862 | 241 | 578 | 43 | 26.7   ( 26 , 27.3 ) | 66.41   ( 66.1 , 66.7 ) | 12.8   ( 12.5 , 13.2 ) | 137.2   ( 136.5 , 137.8 ) | 20.28   ( 19.9 , 20.7 ) | 30.8   ( 30.4 , 31.3 ) | 1   ( 0.3 , 1.8 ) | 0.1   ( -0.3 , 0.6 ) | 0.1   ( -0.3 , 0.5 ) |
| Australia | 34 | 56919 | 897 | 251 | 596 | 50 | 26.9   ( 26.3 , 27.5 ) | 66.22   ( 65.9 , 66.5 ) | 12.4   ( 11.9 , 12.9 ) | 55.9   ( 55.1 , 56.7 ) | 12.44   ( 12 , 12.9 ) | 19.3   ( 18.8 , 19.8 ) | 1.1   ( 0.5 , 1.8 ) | 0.1   ( -0.3 , 0.6 ) | 0.1   ( -0.3 , 0.5 ) |
| Australia | 35 | 89429 | 915 | 236 | 631 | 48 | 25.1   ( 24.5 , 25.6 ) | 68.53   ( 68.3 , 68.8 ) | 10.7   ( 10.3 , 11.1 ) | 31.7   ( 31.1 , 32.3 ) | 8.65   ( 8.3 , 9.1 ) | 9.3   ( 8.9 , 9.6 ) | 1.1   ( 0.5 , 1.6 ) | 0.2   ( -0.2 , 0.5 ) | 0.1   ( -0.2 , 0.3 ) |
| Australia | 36 | 178091 | 1037 | 315 | 655 | 67 | 29.5   ( 28.9 , 30 ) | 63.07   ( 62.8 , 63.4 ) | 12.3   ( 12 , 12.7 ) | 20.1   ( 19.2 , 20.9 ) | 4.63   ( 4.1 , 5.2 ) | 5.9   ( 5.3 , 6.4 ) | 1.5   ( 0.8 , 2.1 ) | 0.2   ( -0.3 , 0.6 ) | 0.1   ( -0.4 , 0.6 ) |
| Australia | 37 | 418632 | 1171 | 310 | 738 | 123 | 26.3   ( 25.9 , 26.7 ) | 62.96   ( 62.7 , 63.2 ) | 11.7   ( 11.1 , 12.3 ) | 11   ( 10.3 , 11.7 ) | 2.36   ( 1.8 , 2.9 ) | 1.8   ( 0.9 , 2.7 ) | 1.6   ( 1.1 , 2.1 ) | 0.2   ( -0.3 , 0.6 ) | 0.1   ( -0.6 , 0.8 ) |
| Australia | 38 | 1128709 | 1467 | 391 | 884 | 192 | 26.1   ( 25.7 , 26.5 ) | 60.15   ( 60 , 60.3 ) | 12.7   ( 12.2 , 13.2 ) | 9.1   ( 8.6 , 9.6 ) | 1.08   ( 0.6 , 1.6 ) | 0.7   ( -0.1 , 1.4 ) | 2.4   ( 1.8 , 2.9 ) | 0.2   ( -0.1 , 0.6 ) | 0.2   ( -0.5 , 0.8 ) |
| Australia | 39 | 1478750 | 1425 | 337 | 842 | 246 | 23.7   ( 22.9 , 24.5 ) | 56.8   ( 56.2 , 57.4 ) | 17.2   ( 16.5 , 17.8 ) | 7.2   ( 6.6 , 7.7 ) | 0.75   ( -0.2 , 1.7 ) | 0.7   ( 0.1 , 1.4 ) | 2.7   ( 2.1 , 3.4 ) | 0.3   ( -0.7 , 1.3 ) | 0.3   ( -0.3 , 0.8 ) |
| Australia | 40 | 1508494 | 1610 | 320 | 1049 | 241 | 19.2   ( 18.6 , 19.8 ) | 65.1   ( 64.9 , 65.3 ) | 15   ( 14.5 , 15.5 ) | 6.4   ( 5.7 , 7.2 ) | 0.93   ( 0.7 , 1.2 ) | 0.7   ( 0.1 , 1.3 ) | 4.2   ( 3.5 , 5 ) | 0.6   ( 0.4 , 0.9 ) | 0.5   ( -0.1 , 1 ) |
| Australia | 41 | 716993 | 1388 | 237 | 950 | 201 | 16.5   ( 15.8 , 17.3 ) | 68.18   ( 68 , 68.4 ) | 15.5   ( 14.9 , 16.2 ) | 9.9   ( 9 , 10.7 ) | 1.79   ( 1.4 , 2.2 ) | 1.2   ( 0.5 , 1.9 ) | 9   ( 8.2 , 9.8 ) | 1.7   ( 1.2 , 2.1 ) | 1.1   ( 0.4 , 1.8 ) |
| Australia | >=42 | 50119 | 1250 | 159 | 897 | 194 | 16.3   ( 14.5 , 18 ) | 76.15   ( 75.8 , 76.5 ) | 16.1   ( 15.3 , 17 ) | 112.2   ( 110 , 114.4 ) | 25.95   ( 24.4 , 27.5 ) | 24.5   ( 23.5 , 25.5 ) | 112.2   ( 110 , 114.4 ) | 26   ( 24.4 , 27.5 ) | 24.5   ( 23.5 , 25.5 ) |

| Denmark | 24 | 326 | 148 | 33 | 109 | 6 | 22.3   ( 22.3 , 22.3 ) | 73.65   ( 73.65 , 73.65 ) | 4.1   ( 4.1 , 4.1 ) | 1000   ( 1000 , 1000 ) | 254.08   ( 254.08 , 254.08 ) | 500   ( 500 , 500 ) | 1.1   ( 1.1 , 1.1 ) | 0.2   ( 0.2 , 0.2 ) | 0   ( 0 , 0 ) |
| --- | --- | --- | --- | --- | --- | --- | --- | --- | --- | --- | --- | --- | --- | --- | --- |
| Denmark | 25 | 439 | 109 | 41 | 62 | 6 | 37.6   ( 37.6 , 37.6 ) | 56.88   ( 56.88 , 56.88 ) | 5.5   ( 5.5 , 5.5 ) | 1000   ( 1000 , 1000 ) | 125.76   ( 125.76 , 125.76 ) | 428.6   ( 428.6 , 428.6 ) | 1.3   ( 1.3 , 1.3 ) | 0.1   ( 0.1 , 0.1 ) | 0   ( 0 , 0 ) |
| Denmark | 26 | 566 | 91 | 26 | 58 | 7 | 28.6   ( 28.6 , 28.6 ) | 63.74   ( 63.74 , 63.74 ) | 7.7   ( 7.7 , 7.7 ) | 684.2   ( 684.2 , 684.2 ) | 107.61   ( 107.61 , 107.61 ) | 87.5   ( 87.5 , 87.5 ) | 0.9   ( 0.9 , 0.9 ) | 0.1   ( 0.1 , 0.1 ) | 0   ( 0 , 0 ) |
| Denmark | 27 | 818 | 78 | 32 | 40 | 6 | 41   ( 41 , 41 ) | 51.28   ( 51.28 , 51.28 ) | 7.7   ( 7.7 , 7.7 ) | 275.9   ( 275.9 , 275.9 ) | 60.15   ( 60.15 , 60.15 ) | 52.2   ( 52.2 , 52.2 ) | 1.1   ( 1.1 , 1.1 ) | 0.1   ( 0.1 , 0.1 ) | 0   ( 0 , 0 ) |
| Denmark | 28 | 1042 | 70 | 34 | 36 | 0 | 48.6   ( 48.6 , 48.6 ) | 51.43   ( 51.43 , 51.43 ) | -   ( - , - ) | 269.8   ( 269.8 , 269.8 ) | 43.53   ( 43.53 , 43.53 ) | -   ( - , - ) | 1.1   ( 1.1 , 1.1 ) | 0.1   ( 0.1 , 0.1 ) | -   ( - , - ) |
| Denmark | 29 | 1258 | 122 | 56 | 55 | 11 | 45.9   ( 45.9 , 45.9 ) | 45.08   ( 45.08 , 45.08 ) | 9   ( 9 , 9 ) | 304.3   ( 304.3 , 304.3 ) | 54.84   ( 54.84 , 54.84 ) | 57   ( 57 , 57 ) | 1.9   ( 1.9 , 1.9 ) | 0.1   ( 0.1 , 0.1 ) | 0   ( 0 , 0 ) |
| Denmark | 30 | 1775 | 117 | 33 | 74 | 10 | 28.2   ( 28.2 , 28.2 ) | 63.25   ( 63.25 , 63.25 ) | 8.5   ( 8.5 , 8.5 ) | 193   ( 193 , 193 ) | 55.06   ( 55.06 , 55.06 ) | 26.5   ( 26.5 , 26.5 ) | 1.1   ( 1.1 , 1.1 ) | 0.1   ( 0.1 , 0.1 ) | 0   ( 0 , 0 ) |
| Denmark | 31 | 2262 | 84 | 26 | 46 | 12 | 31   ( 31 , 31 ) | 54.76   ( 54.76 , 54.76 ) | 14.3   ( 14.3 , 14.3 ) | 132.7   ( 132.7 , 132.7 ) | 25.44   ( 25.44 , 25.44 ) | 35.1   ( 35.1 , 35.1 ) | 0.9   ( 0.9 , 0.9 ) | 0.1   ( 0.1 , 0.1 ) | 0.1   ( 0.1 , 0.1 ) |
| Denmark | 32 | 3362 | 123 | 28 | 80 | 15 | 22.8   ( 22.8 , 22.8 ) | 65.04   ( 65.04 , 65.04 ) | 12.2   ( 12.2 , 12.2 ) | 112.4   ( 112.4 , 112.4 ) | 29.02   ( 29.02 , 29.02 ) | 31.3   ( 31.3 , 31.3 ) | 0.9   ( 0.9 , 0.9 ) | 0.1   ( 0.1 , 0.1 ) | 0.1   ( 0.1 , 0.1 ) |
| Denmark | 33 | 5033 | 73 | 18 | 48 | 7 | 24.7   ( 24.7 , 24.7 ) | 65.75   ( 65.75 , 65.75 ) | 9.6   ( 9.6 , 9.6 ) | 75.9   ( 75.9 , 75.9 ) | 11.42    ( 11.42 , 11.42 ) | 10.5   ( 10.5 , 10.5 ) | 0.6   ( 0.6 , 0.6 ) | 0.1   ( 0.1 , 0.1 ) | 0   ( 0 , 0 ) |
| Denmark | 34 | 7966 | 116 | 23 | 82 | 11 | 19.8   ( 19.8 , 19.8 ) | 70.69   ( 70.69 , 70.69 ) | 9.5   ( 9.5 , 9.5 ) | 39.2   ( 39.2 , 39.2 ) | 12.79   ( 12.79 , 12.79 ) | 10.1   ( 10.1 , 10.1 ) | 0.8   ( 0.8 , 0.8 ) | 0.1   ( 0.1 , 0.1 ) | 0   ( 0 , 0 ) |
| Denmark | 35 | 12051 | 120 | 33 | 71 | 16 | 27.5   ( 27.5 , 27.5 ) | 59.17   ( 59.17 , 59.17 ) | 13.3   ( 13.3 , 13.3 ) | 36.2   ( 36.2 , 36.2 ) | 7.49   ( 7.49 , 7.49 ) | 9   ( 9 , 9 ) | 1.2   ( 1.2 , 1.2 ) | 0.1   ( 0.1 , 0.1 ) | 0.1   ( 0.1 , 0.1 ) |
| Denmark | 36 | 21403 | 138 | 28 | 101 | 9 | 20.3   ( 20.3 , 20.3 ) | 73.19   ( 73.19 , 73.19 ) | 6.5   ( 6.5 , 6.5 ) | 16.5   ( 16.5 , 16.5 ) | 6.29   ( 6.29 , 6.29 ) | 2.4   ( 2.4 , 2.4 ) | 1   ( 1 , 1 ) | 0.2   ( 0.2 , 0.2 ) | 0   ( 0 , 0 ) |
| Denmark | 37 | 51046 | 163 | 42 | 105 | 16 | 25.8   ( 25.8 , 25.8 ) | 64.42   ( 64.42 , 64.42 ) | 9.8   ( 9.8 , 9.8 ) | 12.5   ( 12.5 , 12.5 ) | 2.85   ( 2.85 , 2.85 ) | 1.5   ( 1.5 , 1.5 ) | 1.6   ( 1.6 , 1.6 ) | 0.2   ( 0.2 , 0.2 ) | 0.1   ( 0.1 , 0.1 ) |
| Denmark | 38 | 122362 | 210 | 52 | 127 | 31 | 24.8   ( 24.8 , 24.8 ) | 60.48   ( 60.48 , 60.48 ) | 14.8   ( 14.8 , 14.8 ) | 10.2   ( 10.2 , 10.2 ) | 1.49   ( 1.49 , 1.49 ) | 1   ( 1 , 1 ) | 2.3   ( 2.3 , 2.3 ) | 0.2   ( 0.2 , 0.2 ) | 0.1   ( 0.1 , 0.1 ) |
| Denmark | 39 | 188353 | 202 | 37 | 126 | 39 | 18.3   ( 18.3 , 18.3 ) | 62.38   ( 62.38 , 62.38 ) | 19.3   ( 19.3 , 19.3 ) | 7.1   ( 7.1 , 7.1 ) | 0.96   ( 0.96 , 0.96 ) | 0.7   ( 0.7 , 0.7 ) | 2.1   ( 2.1 , 2.1 ) | 0.3   ( 0.3 , 0.3 ) | 0.2   ( 0.2 , 0.2 ) |
| Denmark | 40 | 235664 | 259 | 35 | 172 | 52 | 13.5   ( 13.5 , 13.5 ) | 66.41   ( 66.41 , 66.41 ) | 20.1   ( 20.1 , 20.1 ) | 5.7   ( 5.7 , 5.7 ) | 1.06   ( 1.06 , 1.06 ) | 0.8   ( 0.8 , 0.8 ) | 2.9   ( 2.9 , 2.9 ) | 0.6   ( 0.6 , 0.6 ) | 0.4   ( 0.4 , 0.4 ) |
| Denmark | 41 | 162793 | 257 | 33 | 175 | 49 | 12.8   ( 12.8 , 12.8 ) | 68.09   ( 68.09 , 68.09 ) | 19.1   ( 19.1 , 19.1 ) | 7.6   ( 7.6 , 7.6 ) | 1.61   ( 1.61 , 1.61 ) | 1   ( 1 , 1 ) | 5.5   ( 5.5 , 5.5 ) | 1.2   ( 1.2 , 1.2 ) | 0.8   ( 0.8 , 0.8 ) |
| Denmark | >=42 | 48407 | 273 | 53 | 176 | 44 | 19.4   ( 19.4 , 19.4 ) | 64.47   ( 64.47 , 64.47 ) | 16.1   ( 16.1 , 16.1 ) | 32.2   ( 32.2 , 32.2 ) | 5.43   ( 5.43 , 5.43 ) | 3   ( 3 , 3 ) | 32.2   ( 32.2 , 32.2 ) | 5.4   ( 5.4 , 5.4 ) | 3   ( 3 , 3 ) |

| England & Wales | 24 | 2664 | 1047 | 168 | 855 | 24 | 15.7   ( 15.2 , 16.1 ) | 81.58   ( 81.5 , 81.7 ) | 2.2   ( 1.7 , 2.8 ) | 331.1   ( 330.7 , 331.4 ) | 284.75   ( 284.6 , 284.9 ) | 111.9   ( 111.7 , 112.1 ) | 1   ( 0.5 , 1.5 ) | 0.3   ( 0.2 , 0.5 ) | 0   ( -0.4 , 0.5 ) |
| --- | --- | --- | --- | --- | --- | --- | --- | --- | --- | --- | --- | --- | --- | --- | --- |
| England & Wales | 25 | 2780 | 729 | 222 | 480 | 27 | 30.3   ( 30 , 30.5 ) | 65.85   ( 65.8 , 65.9 ) | 2.9   ( 1.1 , 4.7 ) | 332.5   ( 332.4 , 332.6 ) | 186.66   ( 186.5 , 186.8 ) | 78.1   ( 76.7 , 79.6 ) | 1.3   ( 1 , 1.6 ) | 0.2   ( 0.1 , 0.3 ) | 0   ( -1.7 , 1.7 ) |
| England & Wales | 26 | 2997 | 794 | 257 | 498 | 39 | 32.4   ( 32.1 , 32.6 ) | 62.41   ( 62.3 , 62.5 ) | 4.8   ( 4.5 , 5.2 ) | 342.1   ( 341.9 , 342.3 ) | 181.1   ( 180.9 , 181.3 ) | 127.1   ( 126.8 , 127.5 ) | 1.6   ( 1.3 , 1.8 ) | 0.2   ( 0 , 0.4 ) | 0.1   ( -0.4 , 0.5 ) |
| England & Wales | 27 | 3539 | 748 | 304 | 396 | 48 | 40.4   ( 40.2 , 40.6 ) | 52.88   ( 52.8 , 53 ) | 6.1   ( 5.4 , 6.9 ) | 341.2   ( 341 , 341.4 ) | 131.63   ( 131.3 , 131.9 ) | 109.8   ( 109.2 , 110.4 ) | 1.8   ( 1.6 , 2.1 ) | 0.2   ( -0.1 , 0.4 ) | 0.1   ( -0.6 , 0.8 ) |
| England & Wales | 28 | 4432 | 655 | 271 | 354 | 30 | 41   ( 40.9 , 41.2 ) | 54.2   ( 54.1 , 54.3 ) | 4   ( 2.8 , 5.2 ) | 254.2   ( 253.9 , 254.5 ) | 101.92   ( 101.7 , 102.1 ) | 46.1   ( 44.9 , 47.4 ) | 1.7   ( 1.4 , 1.9 ) | 0.1   ( -0.1 , 0.3 ) | 0   ( -1.2 , 1.3 ) |
| England & Wales | 29 | 4833 | 640 | 271 | 339 | 30 | 42.2   ( 42 , 42.3 ) | 52.97   ( 52.8 , 53.1 ) | 4.6   ( 4.3 , 4.9 ) | 300.1   ( 299.9 , 300.4 ) | 85.74   ( 85.5 , 86 ) | 45.9   ( 45.5 , 46.4 ) | 1.7   ( 1.4 , 1.9 ) | 0.1   ( -0.1 , 0.4 ) | 0   ( -0.3 , 0.4 ) |
| England & Wales | 30 | 6251 | 625 | 280 | 298 | 47 | 44.8   ( 44.7 , 45 ) | 47.65   ( 47.5 , 47.8 ) | 7.1   ( 6.5 , 7.7 ) | 281.5   ( 281.3 , 281.6 ) | 58.19   ( 58 , 58.4 ) | 56.4   ( 55.7 , 57 ) | 1.7   ( 1.5 , 2 ) | 0.1   ( -0.1 , 0.3 ) | 0.1   ( -0.7 , 0.9 ) |
| England & Wales | 31 | 8002 | 476 | 152 | 286 | 38 | 31.9   ( 31.8 , 32.1 ) | 59.96   ( 59.8 , 60.1 ) | 7.9   ( 7.6 , 8.2 ) | 151.8   ( 151.7 , 152 ) | 43.49   ( 43.3 , 43.6 ) | 41.7   ( 41.4 , 42 ) | 1   ( 0.7 , 1.2 ) | 0.1   ( 0 , 0.3 ) | 0.1   ( -0.3 , 0.4 ) |
| England & Wales | 32 | 11323 | 466 | 122 | 315 | 29 | 26.2   ( 26 , 26.5 ) | 67.04   ( 66.9 , 67.2 ) | 5.6   ( 4.6 , 6.7 ) | 95.3   ( 95.1 , 95.6 ) | 32.89   ( 32.5 , 33.2 ) | 23.1   ( 21.9 , 24.2 ) | 0.8   ( 0.5 , 1 ) | 0.1   ( -0.3 , 0.5 ) | 0   ( -1 , 1.1 ) |
| England & Wales | 33 | 15964 | 506 | 135 | 325 | 46 | 26.4   ( 26.1 , 26.7 ) | 64.13   ( 64 , 64.3 ) | 8.7   ( 8.1 , 9.4 ) | 111.5   ( 111.2 , 111.7 ) | 23.67   ( 23.4 , 23.9 ) | 27.9   ( 27.4 , 28.5 ) | 0.9   ( 0.6 , 1.1 ) | 0.1   ( -0.1 , 0.4 ) | 0.1   ( -0.5 , 0.7 ) |
| England & Wales | 34 | 29999 | 537 | 127 | 362 | 48 | 23.1   ( 22.6 , 23.6 ) | 67.34   ( 67.2 , 67.5 ) | 8.3   ( 7.5 , 9.1 ) | 39.3   ( 38.7 , 40 ) | 14.63   ( 14.4 , 14.9 ) | 16.1   ( 15.2 , 16.9 ) | 0.8   ( 0.2 , 1.4 ) | 0.2   ( -0.1 , 0.4 ) | 0.1   ( -0.8 , 0.9 ) |
| England & Wales | 35 | 46747 | 541 | 116 | 393 | 32 | 21.4   ( 21.2 , 21.6 ) | 72.61   ( 72.6 , 72.7 ) | 5.7   ( 5.1 , 6.2 ) | 23   ( 22.7 , 23.2 ) | 10.44   ( 10.3 , 10.6 ) | 6.5   ( 5.9 , 7.1 ) | 0.8   ( 0.6 , 0.9 ) | 0.2   ( 0 , 0.3 ) | 0.1   ( -0.5 , 0.6 ) |
| England & Wales | 36 | 99958 | 619 | 168 | 419 | 32 | 26.8   ( 26.6 , 27 ) | 67.82   ( 67.7 , 67.9 ) | 5.1   ( 4.7 , 5.5 ) | 13.6   ( 13.2 , 13.9 ) | 5.36   ( 5.1 , 5.6 ) | 2.9   ( 2.4 , 3.3 ) | 1.1   ( 0.7 , 1.5 ) | 0.2   ( 0 , 0.4 ) | 0.1   ( -0.4 , 0.5 ) |
| England & Wales | 37 | 262478 | 659 | 182 | 413 | 64 | 27.2   ( 27 , 27.4 ) | 62.8   ( 62.7 , 62.9 ) | 9.4   ( 8.8 , 10 ) | 6.8   ( 6.3 , 7.4 ) | 2.05   ( 1.8 , 2.3 ) | 1.7   ( 1.1 , 2.4 ) | 1.3   ( 1 , 1.6 ) | 0.2   ( 0 , 0.3 ) | 0.1   ( -0.5 , 0.7 ) |
| England & Wales | 38 | 464502 | 792 | 192 | 517 | 83 | 23.7   ( 23.3 , 24 ) | 65.32   ( 65.1 , 65.5 ) | 10.1   ( 9.5 , 10.7 ) | 7.4   ( 7 , 7.8 ) | 1.45   ( 1.3 , 1.6 ) | 0.9   ( 0.2 , 1.6 ) | 1.7   ( 1.4 , 2 ) | 0.3   ( 0.1 , 0.4 ) | 0.1   ( -0.5 , 0.8 ) |
| England & Wales | 39 | 837873 | 840 | 192 | 555 | 93 | 22.3   ( 21.9 , 22.7 ) | 66   ( 65.9 , 66.1 ) | 11.1   ( 10.7 , 11.5 ) | 6   ( 5.5 , 6.5 ) | 0.86   ( 0.6 , 1.1 ) | 0.5   ( 0.3 , 0.8 ) | 2.2  ( 1.8 , 2.7 ) | 0.3   ( 0.1 , 0.5 ) | 0.2   ( -0.1 , 0.5 ) |
| England & Wales | 40 | 823554 | 879 | 200 | 566 | 113 | 22.1   ( 21.8 , 22.5 ) | 64.69   ( 64.6 , 64.8 ) | 11.9   ( 11 , 12.7 ) | 6.3   ( 5.9 , 6.7 ) | 0.89   ( 0.7 , 1 ) | 0.6   ( -0.3 , 1.6 ) | 3.7   ( 3.3 , 4.1 ) | 0.5   ( 0.4 , 0.6 ) | 0.4   ( -0.6 , 1.3 ) |
| England & Wales | 41 | 506299 | 788 | 135 | 557 | 96 | 16.6   ( 16 , 17.2 ) | 70.6   ( 70.5 , 70.7 ) | 11.9   ( 11.4 , 12.3 ) | 7.3   ( 6.8 , 7.8 ) | 1.48   ( 1.3 , 1.7 ) | 0.8   ( 0.3 , 1.4 ) | 6   ( 5.5 , 6.5 ) | 1.3   ( 1.1 , 1.5 ) | 0.7   ( 0.1 , 1.3 ) |
| England & Wales | >=42 | 74347 | 1485 | 273 | 1034 | 178 | 18.3   ( 18 , 18.5 ) | 69.61   ( 69.6 , 69.7 ) | 11.9   ( 11.6 , 12.1 ) | 68.9   ( 68.8 , 69.1 ) | 18.41   ( 18.3 , 18.6 ) | 10.8   ( 10.5 , 11.2 ) | 68.9   ( 68.8 , 69.1 ) | 18.4   ( 18.3 , 18.6 ) | 10.8   ( 10.5 , 11.2 ) |

| Estonia | 24 | 55 | 13 | 7 | 6 | 0 | 100   ( 100 , 100 ) | 100   ( 100 , 100 ) | -   ( - , - ) | 1000   ( 1000 , 1000 ) | 182.32   ( 181 , 183.6 ) | -   ( - , - ) | 10.1   ( 9.7 , 10.5 ) | 0.2   ( -1.3 , 1.7 ) | -   ( - , - ) |
| --- | --- | --- | --- | --- | --- | --- | --- | --- | --- | --- | --- | --- | --- | --- | --- |
| Estonia | 25 | 54 | 5 | 3 | 2 | 0 | 100   ( 100 , 100 ) | 100   ( 100 , 100 ) | -   ( - , - ) | 693.4   ( 692.1 , 694.6 ) | 223.61   ( 223.3 , 223.9 ) | -   ( - , - ) | 3.1   ( 2.9 , 3.2 ) | 0.1   ( 0 , 0.2 ) | -   ( - , - ) |
| Estonia | 26 | 60 | 9 | 5 | 3 | 1 | 70.7   ( 69.4 , 72.1 ) | 70.71   ( 69.8 , 71.7 ) | 25   ( - , - ) | 577.4   ( 576.4 , 578.3 ) | 192.45   ( 192.1 , 192.8 ) | 1000   ( - , - ) | 3.7   ( 3 , 4.3 ) | 0.1   ( -0.8 , 1.1 ) | 0.3   ( - , - ) |
| Estonia | 27 | 87 | 6 | 5 | 0 | 1 | 100   ( 100 , 100 ) | -   ( - , - ) | 100   ( - , - ) | 816.5   ( 815.9 , 817.1 ) | -   ( - , - ) | 500   ( - , - ) | 7.4   ( 6.8 , 7.9 ) | -   ( - , - ) | 0.2   ( - , - ) |
| Estonia | 28 | 96 | 9 | 5 | 0 | 4 | 73.7   ( 72.6 , 74.7 ) | -   ( - , - ) | 77.5   ( 76.8 , 78.2 ) | 605.7   ( 605.4 , 606 ) | -   ( - , - ) | 346.4   ( 344.9 , 347.9 ) | 4.9   ( 4.1 , 5.6 ) | -   ( - , - ) | 0.4   ( -1 , 1.9 ) |
| Estonia | 29 | 94 | 9 | 6 | 2 | 1 | 69.9   ( 68.9 , 70.9 ) | 66.67   ( - , - ) | 50   ( - , - ) | 644.4   ( 643.5 , 645.3 ) | 125   ( - , - ) | 111.1   ( - , - ) | 3.6   ( 3 , 4.2 ) | 0.2   ( - , - ) | 0.3   ( - , - ) |
| Estonia | 30 | 133 | 9 | 2 | 6 | 1 | 70.7   ( 69.8 , 71.7 ) | 100   ( 100 , 100 ) | 50   ( - , - ) | 707.1   ( 706.1 , 708.1 ) | 78.5   ( 77.7 , 79.2 ) | 166.7   ( - , - ) | 3.1   ( 3.1 , 3.1 ) | 0.2   ( -0.7 , 1 ) | 0.3   ( - , - ) |
| Estonia | 31 | 142 | 7 | 4 | 2 | 1 | 76   ( 74.9 , 77.1 ) | 66.67   ( - , - ) | 100   ( - , - ) | 707.1   ( 706.3 , 707.9 ) | 105.3   ( - , - ) | 200   ( - , - ) | 3.2   ( 3.1 , 3.3 ) | 0.2   ( - , - ) | 0.3   ( - , - ) |
| Estonia | 32 | 240 | 10 | 3 | 3 | 4 | 36.5   ( 36.3 , 36.8 ) | 40.55   ( 38.9 , 42.2 ) | 51.1   ( 49.9 , 52.2 ) | 577.4   ( 575.8 , 578.9 ) | 32.3   ( 31.9 , 32.7 ) | 240.4   ( 239.1 , 241.6 ) | 4.3   ( 3.4 , 5.3 ) | 0.1   ( 0.1 , 0.1 ) | 0.3   ( -0.5 , 1.1 ) |
| Estonia | 33 | 327 | 9 | 7 | 0 | 2 | 80.3   ( 79.3 , 81.2 ) | -   ( - , - ) | 66.7   ( - , - ) | 389.2   ( 388.6 , 389.8 ) | -   ( - , - ) | 285.7   ( - , - ) | 4.1   ( 3.2 , 5 ) | -   ( - , - ) | 0.5   ( - , - ) |
| Estonia | 34 | 555 | 9 | 3 | 3 | 3 | 100   ( - , - ) | 70.71   ( 69.8 , 71.7 ) | 79.4   ( 78.6 , 80.2 ) | 333.3   ( - , - ) | 22.5   ( 21.3 , 23.7 ) | 82.7   ( 82 , 83.4 ) | 9.5   ( - , - ) | 0.2   ( -0.9 , 1.2 ) | 0.2   ( 0.2 , 0.3 ) |
| Estonia | 35 | 1020 | 13 | 9 | 2 | 2 | 92.8   ( 92.6 , 93.1 ) | 100   ( - , - ) | 44.7   ( 42.5 , 47 ) | 222.5   ( 221 , 224 ) | 13.8  ( - , - ) | 42.6   ( 42.5 , 42.8 ) | 8.4   ( 6.9 , 9.8 ) | 0.2   ( - , - ) | 0.2   ( 0.2 , 0.3 ) |
| Estonia | 36 | 1861 | 10 | 6 | 2 | 2 | 63   ( 61.9 , 64.1 ) | 66.67   ( - , - ) | 50   ( 48.1 , 51.9 ) | 89.4   ( 89 , 89.8 ) | 7.7   ( - , - ) | 19.7   ( 19.5 , 19.9 ) | 6.1   ( 5.1 , 7.2 ) | 0.2   ( - , - ) | 0.2   ( 0.2 , 0.3 ) |
| Estonia | 37 | 4698 | 19 | 11 | 6 | 2 | 85.5   ( 85.2 , 85.8 ) | 43.68   ( 41.9 , 45.4 ) | 35.4   ( 34.4 , 36.3 ) | 73.2   ( 72.5 , 74 ) | 2.7  ( 1.2 , 4.2 ) | 6.5   ( 6.4 , 6.5 ) | 13.2   ( 12.6 , 13.7 ) | 0.2   ( -1.3 , 1.7 ) | 0.3   ( 0.2 , 0.3 ) |
| Estonia | 38 | 10191 | 13 | 7 | 3 | 3 | 63.9   ( 62.8 , 65 ) | 57.74   ( 57.3 , 58.1 ) | 50   ( 50 , 50 ) | 32.2   ( 31.2 , 33.1 ) | 1.2  ( 0.2 , 2.3 ) | 2.8   ( 1.7 , 3.9 ) | 7   ( 6 , 8 ) | 0.2   ( -0.8 , 1.1 ) | 0.4   ( -0.7 , 1.4 ) |
| Estonia | 39 | 21264 | 25 | 10 | 13 | 2 | 60.8   ( 60.4 , 61.2 ) | 54.93   ( 53.1 , 56.7 ) | 25   ( 23.1 , 26.9 ) | 43   ( 41.2 , 44.8 ) | 0.9   ( -0.6 , 2.4 ) | 0.9   ( 0.8 , 1 ) | 14.4   ( 12.6 , 16.3 ) | 0.3   ( -1.2 , 1.8 ) | 0.3   ( 0.2 , 0.4 ) |
| Estonia | 40 | 24866 | 22 | 11 | 11 | 0 | 60.8   ( 59.7 , 62 ) | 59.46   ( 58.5 , 60.4 ) | -   ( - , - ) | 31.5   ( 30.5 , 32.4 ) | 0.8  ( -0.6 , 2.2 ) | -   ( - , - ) | 18   ( 17.1 , 19 ) | 0.5   ( -0.9 , 1.9 ) | -   ( - , - ) |
| Estonia | 41 | 15072 | 22 | 12 | 7 | 3 | 58.2   ( 57.1 , 59.2 ) | 35.4   ( 34.4 , 36.4 ) | 40.8   ( 40.3 , 41.4 ) | 52.7   ( 51.5 , 53.8 ) | 0.8   ( 0 , 1.6 ) | 1.9   ( 0.9 , 2.8 ) | 44.9   ( 43.7 , 46.1 ) | 0.7   ( -0.1 , 1.5 ) | 1.7   ( 0.7 , 2.7 ) |
| Estonia | >=42 | 1569 | 25 | 12 | 12 | 1 | 54.5   ( 53.2 , 55.8 ) | 44.78   ( 43.5 , 46.1 ) | 20   ( - , - ) | 275   ( 273.6 , 276.5 ) | 10.8   ( 9.4 , 12.2 ) | 12   ( - , - ) | 275   ( 273.6 , 276.5 ) | 10.8   ( 9.4 , 12.2 ) | 12   ( - , - ) |

| Iran | 24 | 5781 | 3335 | 419 | 2331 | 585 | 12.4   ( 12.1 , 12.7 ) | 69.95   ( 69.9 , 70 ) | 17.3   ( 16.9 , 17.7 ) | 410.6   ( 410.4 , 410.8 ) | 358.4  ( 358.2 , 358.5 ) | 372.6   ( 372.5 , 372.7 ) | 1.3   ( 0.8 , 1.9 ) | 0.6   ( 0.3 , 0.9 ) | 0.9   ( 0.7 , 1.1 ) |
| --- | --- | --- | --- | --- | --- | --- | --- | --- | --- | --- | --- | --- | --- | --- | --- |
| Iran | 25 | 5310 | 2321 | 579 | 1389 | 353 | 24.9   ( 24.7 , 25 ) | 59.83   ( 59.8 , 59.9 ) | 15.2   ( 15 , 15.4 ) | 385.5   ( 385.4 , 385.6 ) | 280.2   ( 279.9 , 280.4 ) | 310   ( 309.7 , 310.2 ) | 1.9   ( 1.5 , 2.3 ) | 0.4   ( 0 , 0.7 ) | 0.6   ( 0.4 , 0.7 ) |
| Iran | 26 | 5680 | 1788 | 530 | 1025 | 233 | 29.7   ( 29.6 , 29.9 ) | 57.34   ( 57.3 , 57.4 ) | 12.8   ( 12.5 , 13.1 ) | 350   ( 349.9 , 350.1 ) | 209.4   ( 209.2 , 209.6 ) | 221.1   ( 220.8 , 221.4 ) | 1.8   ( 1.5 , 2 ) | 0.3   ( 0 , 0.6 ) | 0.4   ( -0.2 , 1 ) |
| Iran | 27 | 5842 | 1245 | 345 | 692 | 208 | 27.8   ( 27.6 , 27.9 ) | 55.51   ( 55.5 , 55.6 ) | 16.6   ( 16.4 , 16.8 ) | 297.5   ( 297.3 , 297.7 ) | 144.8   ( 144.6 , 145 ) | 183.9   ( 183.6 , 184.2 ) | 1.1   ( 0.7 , 1.6 ) | 0.2   ( -0.2 , 0.5 ) | 0.3   ( -0.1 , 0.7 ) |
| Iran | 28 | 8154 | 1514 | 500 | 765 | 249 | 33.1   ( 33 , 33.1 ) | 50.32   ( 50.2 , 50.5 ) | 16.3   ( 15.9 , 16.7 ) | 292.6   ( 292.5 , 292.7 ) | 125.7   ( 125.6 , 125.8 ) | 133.6   ( 133.3 , 133.9 ) | 1.7   ( 1.5 , 1.8 ) | 0.2   ( 0 , 0.4 ) | 0.4   ( 0 , 0.8 ) |
| Iran | 29 | 7621 | 1145 | 363 | 563 | 219 | 31.6   ( 31.3 , 31.8 ) | 49.01   ( 48.9 , 49.1 ) | 19.1   ( 18.9 , 19.3 ) | 264.2   ( 263.9 , 264.5 ) | 98.7   ( 98.5 , 99 ) | 135.6   ( 135.4 , 135.9 ) | 1.2   ( 0.7 , 1.7 ) | 0.1   ( -0.1 , 0.4 ) | 0.3   ( 0.2 , 0.5 ) |
| Iran | 30 | 11102 | 1334 | 388 | 620 | 326 | 28.6   ( 28.3 , 28.9 ) | 46.45   ( 46.4 , 46.5 ) | 24.4   ( 24.1 , 24.7 ) | 216.1   ( 215.8 , 216.4 ) | 78.7   ( 78.4 , 78.9 ) | 121   ( 120.5 , 121.4 ) | 1.3   ( 0.7 , 1.8 ) | 0.2   ( -0.2 , 0.5 ) | 0.5   ( 0.1 , 0.9 ) |
| Iran | 31 | 12942 | 1162 | 371 | 577 | 214 | 31.8   ( 31.7 , 31.9 ) | 49.2   ( 49 , 49.4 ) | 18.4   ( 17.9 , 18.8 ) | 201.4   ( 201.1 , 201.6 ) | 60.0   ( 59.7 , 60.4 ) | 80.4   ( 80.2 , 80.7 ) | 1.3   ( 0.9 , 1.6 ) | 0.1   ( -0.4 , 0.7 ) | 0.3   ( 0.1 , 0.6 ) |
| Iran | 32 | 20704 | 1442 | 375 | 816 | 251 | 26   ( 26 , 26.1 ) | 56.61   ( 56.6 , 56.7 ) | 17.2   ( 17 , 17.5 ) | 159.9   ( 159.6 , 160.1 ) | 51.5   ( 51.2 , 51.8 ) | 65.1   ( 64.8 , 65.4 ) | 1.3   ( 0.9 , 1.6 ) | 0.2   ( -0.2 , 0.6 ) | 0.4   ( 0 , 0.8 ) |
| Iran | 33 | 27920 | 1228 | 281 | 777 | 170 | 22.8   ( 22.7 , 22.9 ) | 63.32   ( 63.3 , 63.4 ) | 13.8   ( 13.7 , 13.9 ) | 127.8   ( 127.5 , 128.2 ) | 34.7   ( 34.4 , 35 ) | 39.9   ( 39.7 , 40.1 ) | 1   ( 0.6 , 1.3 ) | 0.2   ( -0.1 , 0.5 ) | 0.3   ( 0 , 0.5 ) |
| Iran | 34 | 51646 | 1503 | 386 | 903 | 214 | 25.7   ( 25.5 , 25.8 ) | 59.86   ( 59.8 , 60 ) | 14.1   ( 13.7 , 14.5 ) | 83.7   ( 83.4 84.1 ) | 22.2   ( 21.8 , 22.6 ) | 27.8   ( 27.7 , 28 ) | 1.3   ( 0.8 , 1.9 ) | 0.2   ( -0.3 , 0.7 ) | 0.3   ( 0.2 , 0.5 ) |
| Iran | 35 | 81681 | 1584 | 433 | 913 | 238 | 27.3   ( 27.3 , 27.4 ) | 57.47   ( 57.4 , 57.5 ) | 15   ( 14.8 , 15.3 ) | 59.2   ( 59 , 59.5 ) | 14.4  ( 14.1 , 14.8 ) | 19   ( 18.9 , 19.2 ) | 1.5   ( 1.1 , 1.9 ) | 0.2   ( -0.2 , 0.7 ) | 0.4   ( 0.3 , 0.5 ) |
| Iran | 36 | 162608 | 1700 | 514 | 954 | 232 | 30.3   ( 30.1 , 30.5 ) | 55.96   ( 55.9 , 56 ) | 13.6   ( 13.5 , 13.7 ) | 36.3   ( 36 , 36.6 ) | 7.6   ( 7.1 , 8.1 ) | 9.6   ( 9.1 , 10.1 ) | 1.9   ( 1.5 , 2.2 ) | 0.3   ( -0.3 , 0.8 ) | 0.4   ( -0.2 , 1 ) |
| Iran | 37 | 420894 | 1807 | 452 | 1109 | 246 | 24.8   ( 24.7 , 25 ) | 61.48   ( 61.4 , 61.5 ) | 13.5   ( 13.3 , 13.7 ) | 16   ( 15.5 , 16.4 ) | 3.4  ( 3.1 , 3.8 ) | 3.5   ( 2.9 , 4.1 ) | 1.7   ( 1.1 , 2.3 ) | 0.3   ( -0.1 , 0.8 ) | 0.4   ( -0.2 , 1.1 ) |
| Iran | 38 | 1493934 | 2242 | 489 | 1455 | 298 | 21.8   ( 21.8 , 21.9 ) | 64.97   ( 64.9 , 65 ) | 13.1   ( 12.9 , 13.3 ) | 7.8   ( 7.5 , 8.1 ) | 1.2  ( 1 , 1.5 ) | 1.3   ( 1 , 1.6 ) | 2.1   ( 1.7 , 2.5 ) | 0.5   ( 0.2 , 0.7 ) | 0.6   ( 0.3 , 0.9 ) |
| Iran | 39 | 1414876 | 1578 | 326 | 1021 | 231 | 20.6   ( 20.5 , 20.7 ) | 64.71   ( 64.7 , 64.7 ) | 14.6   ( 14.5 , 14.7 ) | 3.9   ( 3.6 , 4.2 ) | 0.9   ( 0.5 , 1.3 ) | 1.3   ( 0.9 , 1.8 ) | 1.9   ( 1.6 , 2.2 ) | 0.5   ( 0.1 , 0.9 ) | 0.8   ( 0.4 , 1.3 ) |
| Iran | 40 | 874705 | 1000 | 211 | 666 | 123 | 21   ( 20.7 , 21.4 ) | 66.36   ( 66.2 , 66.5 ) | 12.3   ( 12.2 , 12.4 ) | 3.2   ( 2.9 , 3.4 ) | 0.9  ( 0.5 , 1.3 ) | 1.5   ( 1.3 , 1.6 ) | 2.4   ( 2.1 , 2.7 ) | 0.8   ( 0.3 , 1.2 ) | 1.2   ( 1 , 1.4 ) |
| Iran | 41 | 178584 | 236 | 70 | 147 | 19 | 29.4   ( 29.2 , 29.6 ) | 62.34   ( 62.2 , 62.5 ) | 8.1   ( 7.9 , 8.2 ) | 3.6   ( 3.5 , 3.8 ) | 1.0  ( 0.7 , 1.4 ) | 1.2   ( 0.9 , 1.5 ) | 3.3   ( 3.2 , 3.5 ) | 1   ( 0.7 , 1.3 ) | 1.1   ( 0.9 , 1.4 ) |
| Iran | >=42 | 9733 | 86 | 30 | 45 | 11 | 35.1   ( 35 , 35.3 ) | 52.55   ( 52.5 , 52.6 ) | 11.3   ( 10.3 , 12.2 ) | 16.5   ( 16 , 17.1 ) | 6.4   ( 5.8 , 6.9 ) | 11.1   ( 9.9 , 12.4 ) | 16.5   ( 16 , 17.1 ) | 6.4   ( 5.8 , 6.9 ) | 11.1   ( 9.9 , 12.4 ) |

| Lebanon | 24 | 84 | 51 | 17 | 20 | 14 | 57.1   ( 55.7 , 58.4 ) | 39.87   ( 38.7 , 41 ) | 68.7   ( 67.6 , 69.7 ) | 906.6   ( 906.1 , 907 ) | 306.2   ( 304.8 , 307.6 ) | 945.7   ( 945.5 , 946 ) | 1.4   ( 0.7 , 2 ) | 0.1   ( -1.6 , 1.9 ) | 1.7   ( 0.7 , 2.8 ) |
| --- | --- | --- | --- | --- | --- | --- | --- | --- | --- | --- | --- | --- | --- | --- | --- |
| Lebanon | 25 | 133 | 31 | 12 | 4 | 15 | 61.9   ( 60.8 , 63 ) | 28.77   ( 27.2 , 30.3 ) | 61.4   ( 60.4 , 62.4 ) | 565.6   ( 564.7 , 566.5 ) | 132.3  ( 131.9 , 132.7 ) | 707.1   ( 706.4 , 707.9 ) | 1.2   ( -0.1 , 2.5 ) | 0.1   ( -0.5 , 0.8 ) | 1.1   ( -1 , 3.2 ) |
| Lebanon | 26 | 163 | 47 | 14 | 20 | 13 | 38.8   ( 37.8 , 39.8 ) | 58.88   ( 58.1 , 59.6 ) | 41.5   ( 39.9 , 43.1 ) | 635.1   ( 634.1 , 636.1 ) | 244.3   ( 243 , 245.6 ) | 472.1   ( 471.4 , 472.9 ) | 1.4   ( 0 , 2.7 ) | 0.2   ( -1.4 , 1.7 ) | 0.7   ( 0.1 , 1.3 ) |
| Lebanon | 27 | 226 | 23 | 10 | 9 | 4 | 49.7   ( 48.6 , 50.8 ) | 57.91   ( 57.5 , 58.3 ) | 46.4   ( 45 , 47.8 ) | 521   ( 520 , 522 ) | 164.9   ( 164.4 , 165.5 ) | 202.2   ( 200.5 , 203.9 ) | 1.1   ( 0.2 , 1.9 ) | 0.1   ( -0.8 , 1 ) | 0.5   ( -0.5 , 1.6 ) |
| Lebanon | 28 | 359 | 22 | 13 | 4 | 5 | 64.6   ( 63.6 , 65.5 ) | 61.24   ( 60.7 , 61.8 ) | 34.9   ( 34.3 , 35.6 ) | 382.2   ( 381 , 383.4 ) | 106.2   ( 105.1 , 107.3 ) | 203.3   ( 202.3 , 204.3 ) | 1.3   ( 0.1 , 2.4 ) | 0.2   ( -0.1 , 0.5 ) | 0.5   ( -0.6 , 1.5 ) |
| Lebanon | 29 | 425 | 20 | 12 | 4 | 4 | 67.9   ( 67.3 , 68.5 ) | 45.18   ( 44 , 46.4 ) | 39.8   ( 38.3 , 41.2 ) | 287.5   ( 286.3 , 288.7 ) | 41.4   ( 40.8 , 42 ) | 217.1   ( 214.7 , 219.5 ) | 1.3   ( 0.5 , 2.1 ) | 0.1   ( -0.1 , 0.3 ) | 0.4   ( -0.5 , 1.3 ) |
| Lebanon | 30 | 545 | 12 | 7 | 3 | 2 | 89.1   ( 88.5 , 89.6 ) | 81.65   ( 81.1 , 82.2 ) | 40.8   ( 40.3 , 41.4 ) | 306.8   ( 305.9 , 07.6 ) | 89.1   ( 88.5 , 89.7 ) | 114.7   ( 112.5 , 116.9 ) | 0.9   ( 0.5 , 1.4 ) | 0.2   ( 0.1 , 0.3 ) | 0.5   ( 0 , 1 ) |
| Lebanon | 31 | 641 | 10 | 5 | 2 | 3 | 75.8   ( 75 , 76.5 ) | 50   ( 50 , 50 ) | 63   ( 62.2 , 63.8 ) | 228.7   ( 227.7 , 229.6 ) | 28.6  ( 28.5 , 28.8 ) | 71.9   ( 70.8 , 73 ) | 0.8   ( 0.4 , 1.2 ) | 0.1   ( -0.4 , 0.5 ) | 0.4   ( 0.1 , 0.6 ) |
| Lebanon | 32 | 1202 | 14 | 6 | 4 | 4 | 76   ( 75.3 , 76.6 ) | 40.82   ( 39.6 , 42 ) | 55   ( 53.6 , 56.4 ) | 138.7   ( 138.3 , 139.2 ) | 16.9   ( 16.1 , 17.8 ) | 63.3   ( 62.1 , 64.5 ) | 1.1   ( 0.4 , 1.8 ) | 0.1   ( -1.1 , 1.2 ) | 0.5   ( -0.5 , 1.4 ) |
| Lebanon | 33 | 1482 | 18 | 6 | 5 | 7 | 59.5   ( 58.2 , 60.8 ) | 70.71   ( 69.9 , 71.5 ) | 63   ( 61.9 , 64.1 ) | 180.8   ( 179.2 , 182.4 ) | 16.6   ( 15.8 , 17.4 ) | 67.9   ( 66.3 , 69.4 ) | 1.2   ( -0.1 , 2.5 ) | 0.1   ( -0.6 , 0.8 ) | 0.5   ( -0.2 , 1.2 ) |
| Lebanon | 34 | 2623 | 18 | 7 | 5 | 6 | 84.1   ( 83.4 , 84.8 ) | 72.11   ( 71.4 , 72.8 ) | 63   ( 61.9 , 64.1 ) | 96.8   ( 95.8 , 97.7 ) | 11.6   ( 10.6 , 12.6 ) | 47.2   ( 45.2 , 49.1 ) | 1.3   ( 0.2 , 2.4 ) | 0.1   ( -1.2 , 1.4 ) | 0.5   ( -0.2 , 1.2 ) |
| Lebanon | 35 | 4583 | 14 | 4 | 5 | 5 | 76   ( 74.9 , 77.1 ) | 84.09   ( 83.4 , 84.8 ) | 76   ( 75.3 , 76.6 ) | 26.7   ( 26.4 , 27 ) | 6.2  ( 4.9 , 7.5 ) | 40.3   ( 38.8 , 41.7 ) | 0.9   ( 0.6 , 1.2 ) | 0.1   ( -1 , 1.2 ) | 0.7   ( -0.4 , 1.8 ) |
| Lebanon | 36 | 11700 | 30 | 11 | 7 | 12 | 50.5   ( 49.7 , 51.3 ) | 60.43   ( 59.5 , 61.4 ) | 49   ( 47.8 , 50.2 ) | 20.1   ( 18.9 , 21.3 ) | 1.9  ( 0.8 , 3.1 ) | 12.6   ( 11 , 14.2 ) | 1.4   ( 0.4 , 2.3 ) | 0.1   ( -1 , 1.1 ) | 0.7   ( -0.7 , 2.1 ) |
| Lebanon | 37 | 30887 | 20 | 8 | 6 | 6 | 63.5   ( 62.3 , 64.7 ) | 55.03   ( 54.2 , 55.9 ) | 6   ( 75.3 , 76.6 ) | 9.1   ( 8.3 , 10 ) | 0.9  ( -0.2 , 2 ) | 3.2   ( 2.1 , 4.2 ) | 1.2   ( 0.4 , 1.9 ) | 0.1   ( -1 , 1.3 ) | 0.6   ( -0.5 , 1.7 ) |
| Lebanon | 38 | 70381 | 29 | 13 | 9 | 7 | 55   ( 53.7 , 56.3 ) | 53.65   ( 53 , 54.3 ) | 54.2   ( 53.2 , 55.2 ) | 5.5   ( 4.6 , 6.4 ) | 0.4  ( -1 , 1.8 ) | 2.3   ( 1 , 3.5 ) | 1.7   ( 0.8 , 2.5 ) | 0.1   ( -0.9 , 1.2 ) | 0.9   ( -0.1 , 1.8 ) |
| Lebanon | 39 | 67003 | 27 | 10 | 8 | 9 | 59   ( 57.9 , 60.2 ) | 41.63   ( 40.6 , 42.6 ) | 56.1   ( 54.8 , 57.5 ) | 5   ( 3.7 , 6.4 ) | 0.4  ( -0.5 , 1.2 ) | 2.1   ( 1.1 , 3.1 ) | 2.4   ( 1.1 , 3.6 ) | 0.2   ( -0.7 , 1 ) | 1.2   ( 0.2 , 2.2 ) |
| Lebanon | 40 | 51668 | 28 | 19 | 7 | 2 | 77.3   ( 76.6 , 78.1 ) | 45.03   ( 44.1 , 46 ) | 25.8   ( 25.1 , 26.5 ) | 6.2   ( 5.2 , 7.1 ) | 0.4   ( -0.4 , 1.2 ) | 2.2   ( 2.2 , 2.3 ) | 4.8   ( 3.9 , 5.7 ) | 0.3   ( -0.5 , 1.2 ) | 2   ( 1.9 , 2.1 ) |
| Lebanon | 41 | 7089 | 25 | 11 | 10 | 4 | 43.9   ( 43.1 , 44.8 ) | 53.65   ( 52.7 , 54.5 ) | 53.7   ( 52.8 , 54.6 ) | 22.5   ( 21.4 , 23.6 ) | 5.9   ( 5.2 , 6.7 ) | 18.6   ( 17.7 , 19.5 ) | 18.6   ( 17.5 , 19.7 ) | 5.5   ( 4.8 , 6.3 ) | 16.9   ( 15.9 , 17.9 ) |
| Lebanon | >=42 | 762 | 28 | 15 | 9 | 4 | 59.1   ( 58.2 , 60 ) | 51.07   ( 50.1 , 52.1 ) | 38.2   ( 37.2 , 39.1 ) | 86.3   ( 85.1 , 87.5 ) | 41.0   ( 39.9 , 42.1 ) | 333.3   ( 333.3 , 333.3 ) | 86.3   ( 85.1 , 87.5 ) | 41   ( 39.9 , 42.1 ) | 333.3   ( 333.3 , 333.3 ) |

| Malaysia | 24 | 462 | 280 | 52 | 193 | 35 | 15.8   ( 14.6 , 17.1 ) | 67.27   ( 66.9 , 67.6 ) | 11.8   ( 10.9 , 12.7 ) | 759   ( 758.4 , 759.6 ) | 403.3  ( 402.8 , 403.8 ) | 156.9   ( 156 , 157.8 ) | 0.3   ( -0.8 , 1.4 ) | 0.3   ( -0.4 , 1 ) | 0.5   ( -0.5 , 1.4 ) |
| --- | --- | --- | --- | --- | --- | --- | --- | --- | --- | --- | --- | --- | --- | --- | --- |
| Malaysia | 25 | 697 | 263 | 75 | 153 | 35 | 27.7   ( 27.1 , 28.3 ) | 58.24   ( 58 , 58.5 ) | 11.9   ( 10.5 , 13.3 ) | 608.3   ( 608.1 , 608.6 ) | 271.4  ( 271 , 271.9 ) | 110.3   ( 108.7 , 111.8 ) | 0.5   ( -0.2 , 1.2 ) | 0.2   ( -0.3 , 0.8 ) | 0.4   ( -1.3 , 2.2 ) |
| Malaysia | 26 | 959 | 304 | 122 | 150 | 32 | 38   ( 37.5 , 38.6 ) | 49.26   ( 48.9 , 49.6 ) | 9.7   ( 8.6 , 10.7 ) | 686.9   ( 686.5 , 687.2 ) | 213.4   ( 213.1 , 213.7 ) | 76.5   ( 75.4 , 77.5 ) | 0.8   ( 0.2 , 1.5 ) | 0.2   ( -0.1 , 0.6 ) | 0.4   ( -0.7 , 1.5 ) |
| Malaysia | 27 | 1152 | 341 | 153 | 158 | 30 | 43.6   ( 43.2 , 44 ) | 45.92   ( 45.6 , 46.2 ) | 8.6   ( 7.7 , 9.4 ) | 631.6   ( 631.4 , 631.7 ) | 198.3  ( 198 , 198.5 ) | 61.6   ( 60.7 , 62.5 ) | 1   ( 0.3 , 1.8 ) | 0.2   ( -0.3 , 0.7 ) | 0.4   ( -0.4 , 1.2 ) |
| Malaysia | 28 | 1568 | 346 | 168 | 151 | 27 | 48   ( 47.7 , 48.4 ) | 43.38   ( 43.1 , 43.7 ) | 6.8   ( 5.3 , 8.4 ) | 529.1   ( 528.8 , 529.3 ) | 141.1   ( 140.5 , 141.6 ) | 44.1   ( 42.6 , 45.5 ) | 1.2   ( 0.5 , 1.8 ) | 0.2   ( -0.5 , 0.9 ) | 0.4   ( -1.2 , 1.9 ) |
| Malaysia | 29 | 1963 | 340 | 179 | 130 | 31 | 53.1   ( 52.9 , 53.2 ) | 37.99   ( 37.7 , 38.3 ) | 7   ( 5.7 , 8.4 ) | 495.8   ( 495.4 , 496.1 ) | 105.5  ( 105 , 106.1 ) | 31.6   ( 30 , 33.2 ) | 1.2   ( 0.9 , 1.6 ) | 0.2   ( -0.4 , 0.8 ) | 0.3   ( -1.3 , 1.9 ) |
| Malaysia | 30 | 2809 | 346 | 151 | 163 | 32 | 42.9   ( 42.4 , 43.4 ) | 45.79   ( 45.4 , 46.2 ) | 8   ( 6.9 , 9.2 ) | 324.7   ( 324.1 , 325.4 ) | 92.6   ( 92 , 93.3 ) | 26   ( 24.9 , 27.2 ) | 1   ( 0.1 , 1.9 ) | 0.2   ( -0.5 , 1 ) | 0.4   ( -0.9 , 1.7 ) |
| Malaysia | 31 | 3674 | 332 | 159 | 139 | 34 | 47.5   ( 47.3 , 47.8 ) | 41.12   ( 40.8 , 41.4 ) | 8.8   ( 7.5 , 10.2 ) | 277.8   ( 277.4 , 278.1 ) | 63.7  ( 63.3 , 64.1 ) | 22.2   ( 20.9 , 23.5 ) | 1.1   ( 0.6 , 1.6 ) | 0.2   ( -0.2 , 0.6 ) | 0.4   ( -1.1 , 1.9 ) |
| Malaysia | 32 | 5325 | 364 | 149 | 171 | 44 | 40.9   ( 40.6 , 41.2 ) | 46.47   ( 46.3 , 46.7 ) | 10.5   ( 9.3 , 11.7 ) | 233.9   ( 233.5 , 234.2 ) | 51.6  ( 50.9 , 52.2 ) | 22.2   ( 20.8 , 23.6 ) | 1.1   ( 0.7 , 1.5 ) | 0.3   ( -0.4 , 0.9 ) | 0.6   ( -0.9 , 2 ) |
| Malaysia | 33 | 7999 | 342 | 147 | 171 | 24 | 41.8   ( 41.4 , 42.3 ) | 50.24   ( 49.9 , 50.6 ) | 5.5   ( 4.3 , 6.8 ) | 188.9   ( 188.1 , 189.6 ) | 32.3   ( 31.9 , 32.6 ) | 8   ( 6.3 , 9.7 ) | 1   ( 0.2 , 1.9 ) | 0.3   ( -0.1 , 0.6 ) | 0.3   ( -1.1 , 1.7 ) |
| Malaysia | 34 | 13275 | 414 | 182 | 209 | 23 | 42.7   ( 42.3 , 43.1 ) | 49.98   ( 49.6 , 50.4 ) | 4.9   ( 3.7 , 6.1 ) | 107.3   ( 106.5 , 108.2 ) | 23.4   ( 23 , 23.8 ) | 6.4   ( 5.1 , 7.6 ) | 1.3   ( 0.3 , 2.2 ) | 0.3   ( -0.1 , 0.7 ) | 0.3   ( -1 , 1.7 ) |
| Malaysia | 35 | 21157 | 396 | 178 | 181 | 37 | 44.5   ( 44.2 , 44.8 ) | 45.57   ( 45.4 , 45.8 ) | 8.5   ( 7.6 , 9.4 ) | 61.7   ( 61.4 , 62 ) | 12.2   ( 11.7 , 12.6 ) | 9.2   ( 8.2 , 10.1 ) | 1.3   ( 1 , 1.6 ) | 0.3   ( -0.2 , 0.8 ) | 0.6   ( -0.6 , 1.7 ) |
| Malaysia | 36 | 43068 | 413 | 189 | 184 | 40 | 45.9   ( 45.6 , 46.2 ) | 43.4   ( 43 , 43.7 ) | 8.6   ( 7.6 , 9.6 ) | 29.3   ( 28.9 , 29.8 ) | 5.6   ( 5 , 6.3 ) | 7.1   ( 6.1 , 8 ) | 1.4   ( 0.9 , 1.9 ) | 0.3   ( -0.3 , 0.9 ) | 0.6   ( -0.3, 1.6 ) |
| Malaysia | 37 | 108594 | 498 | 210 | 244 | 44 | 41.4   ( 41.1 , 41.8 ) | 48.58   ( 48.3 , 48.9 ) | 8.5   ( 7.9 , 9.1 ) | 14.5   ( 13.8 , 15.2 ) | 2.9   ( 2.2 , 3.6 ) | 4   ( 3.3 , 4.6 ) | 1.6   ( 1 , 2.3 ) | 0.4   ( -0.3 , 1.1 ) | 0.8   ( 0.1 , 1.6 ) |
| Malaysia | 38 | 197312 | 405 | 159 | 202 | 44 | 39.4   ( 39.1 , 39.7 ) | 49.27   ( 49 , 49.5 ) | 10.1   ( 9.6 , 10.7 ) | 6.7   ( 6.3 , 7 ) | 1.3  ( 0.7 , 1.8 ) | 2.7   ( 1.9 , 3.4 ) | 1.4   ( 1.1 , 1.8 ) | 0.4   ( -0.2 , 0.9 ) | 1   ( 0.3 , 1.8 ) |
| Malaysia | 39 | 211965 | 378 | 156 | 187 | 35 | 40.8   ( 40.5 , 41.1 ) | 49.46   ( 49.2 , 49.7 ) | 8.9   ( 8.4 , 9.4 ) | 5   ( 4.4 , 5.6 ) | 1.1  ( 0.7 , 1.6 ) | 2.6   ( 1.8 , 3.4 ) | 1.7   ( 1.1 , 2.4 ) | 0.5   ( 0.1 , 1 ) | 1.4   ( 0.6 , 2.2 ) |
| Malaysia | 40 | 186796 | 423 | 236 | 169 | 18 | 53.4   ( 53.2 , 53.7 ) | 41.19   ( 40.8 , 41.5 ) | 3.8   ( 2.6 , 5 ) | 6.3   ( 5.7 , 6.9 ) | 1.2   ( 0.7 , 1.6 ) | 1.8   ( 0.7 , 3 ) | 3.8   ( 3.2 , 4.5 ) | 0.9   ( 0.4 , 1.3 ) | 1.4   ( 0.3 , 2.6 ) |
| Malaysia | 41 | 66652 | 129 | 78 | 48 | 3 | 58.8   ( 58.5 , 59 ) | 37.93   ( 37.6 , 38.3 ) | 6.1   ( 5.3 , 6.9 ) | 4.4   ( 3.6 , 5.3 ) | 1.0  ( 0.3 , 1.7 ) | 3.7   ( 3.5 , 3.8 ) | 3.3   ( 2.4 , 4.1 ) | 0.9   ( 0.2 , 1.5 ) | 3.4   ( 3.2 , 3.5 ) |
| Malaysia | >=42 | 12258 | 41 | 30 | 8 | 3 | 69.7   ( 69 , 70.5 ) | 28.85   ( 27.5 , 30.2 ) | 20   ( 20 , 20 ) | 4.3   ( 3.2 , 5.3 ) | 2.0   ( 0.9 , 3.1 ) | 53.8   ( 53.2 , 54.4 ) | 4.3   ( 3.2 , 5.3 ) | 2   ( 0.9 , 3.1 ) | 53.8   ( 53.2 , 54.4 ) |

| Mexico | 24 | 5640 | 13339 | 1 | 5497 | 7841 | 0.1   ( - , - ) | 37.73   ( 36.8 , 38.6 ) | 56.8   ( 56.3 , 57.3 ) | 250   ( - , - ) | 483.1   ( 482.7 , 483.5 ) | 949.6   ( 949.6 , 949.6 ) | 0   ( - , - ) | 0.3   ( -0.6 , 1.2 ) | 3.5   ( 3 , 4 ) |
| --- | --- | --- | --- | --- | --- | --- | --- | --- | --- | --- | --- | --- | --- | --- | --- |
| Mexico | 25 | 7251 | 9556 | 0 | 5820 | 3736 | -   ( - , - ) | 53.94   ( 52.8 , 55.1 ) | 28.4   ( 26.8 , 30 ) | -   ( - , - ) | 430.2   ( 429.7 , 430.7 ) | 801.4   ( 801.1 , 801.7 ) | -   ( - , - ) | 0.3   ( -0.8 , 1.3 ) | 1.3   ( -0.3 , 2.8 ) |
| Mexico | 26 | 11900 | 7780 | 0 | 5165 | 2615 | -    ( - , - ) | 62.88   ( 62.1 , 63.7 ) | 23.9   ( 22.3 , 25.5 ) | -    ( - , - ) | 305.9  ( 305.5 , 306.4 ) | 734.5   ( 734.1 , 734.9 ) | -   ( - , - ) | 0.2   ( -0.5 , 1 ) | 0.9   ( -0.8 , 2.5 ) |
| Mexico | 27 | 14927 | 6230 | 0 | 4709 | 1521 | -   ( - , - ) | 75.4   ( 75 , 75.8 ) | 20.1   ( 18.9 , 21.2 ) | -    ( - , - ) | 260.2   ( 260.1 , 260.3 ) | 580.5   ( 580 , 581.1 ) | -   ( - , - ) | 0.2   ( 0 , 0.5 ) | 0.6   ( -0.6 , 1.8 ) |
| Mexico | 28 | 21843 | 8512 | 0 | 7729 | 783 | -   ( - , - ) | 90.79   ( 90.8 , 90.8 ) | 9.1   ( 8.9 , 9.4 ) | -    ( - , - ) | 287.9   ( 287.7 , 288.1 ) | 372.5   ( 372.2 , 372.9 ) | -   ( - , - ) | 0.4   ( 0.2 , 0.6 ) | 0.4   ( 0.1 , 0.6 ) |
| Mexico | 29 | 20284 | 5032 | 0 | 4598 | 434 | -   ( - , - ) | 91.34   ( 91.3 , 91.4 ) | 8.6   ( 8.3 , 8.9 ) | -    ( - , - ) | 208.0  ( 207.8 , 208.3 ) | 305.2   ( 305 , 305.5 ) | -   ( - , - ) | 0.2   ( 0 , 0.5 ) | 0.2   ( -0.1 , 0.5 ) |
| Mexico | 30 | 34648 | 7166 | 336 | 6361 | 469 | 4.6   ( 4.2 , 5 ) | 88.78   ( 88.7 , 88.8 ) | 6.4   ( 6 , 6.8 ) | 94.6    ( 94.1 , 95.1 ) | 177.3  ( 177.2 , 177.5 ) | 180.5   ( 180.2 , 180.9 ) | 0.2   ( -0.3 , 0.7 ) | 0.3   ( 0.1 , 0.5 ) | 0.2   ( -0.3 , 0.7 ) |
| Mexico | 31 | 31218 | 5321 | 680 | 4591 | 50 | 12.8   ( 12.6 , 12.9 ) | 86.25   ( 86.2 , 86.3 ) | 0.8   ( -0.3 , 2 ) | 180.6   ( 180.4 , 180.9 ) | 149.4   ( 149.1 , 149.9 ) | 28.7   ( 27 , 30.5 ) | 0.4   ( 0.2 , 0.6 ) | 0.2   ( 0 , 0.4 ) | 0   ( -1.1 , 1.2 ) |
| Mexico | 32 | 68982 | 7258 | 207 | 6928 | 123 | 2.8   ( 2.5 , 3.2 ) | 95.43   ( 95.4 , 95.4 ) | 1.6   ( 1 , 2.3 ) | 33.7   ( 33.2 , 34.1 ) | 101.9   ( 101.6 , 102.2 ) | 47   ( 46.4 , 47.7 ) | 0.1   ( -0.3 , 0.5 ) | 0.4   ( 0.1 , 0.6 ) | 0.1   ( -0.7 , 0.8 ) |
| Mexico | 33 | 91517 | 6128 | 0 | 6025 | 103 | -   ( - , - ) | 98.32   ( 98.3 , 98.3 ) | 1.6   ( 0.8 , 2.3 ) | -   ( - , - ) | 65.5   ( 65.2 , 65.7 ) | 27.3   ( 26.5 , 28 ) | -   ( - , - ) | 0.3   ( 0.1 , 0.5 ) | 0   ( -0.7 , 0.8 ) |
| Mexico | 34 | 166017 | 7505 | 0 | 7040 | 465 | -   ( - , - ) | 93.8   ( 93.8 , 93.8 ) | 6.1   ( 5.9 , 6.4 ) | -   ( - , - ) | 46.6   ( 46.4 , 46.9 ) | 49   ( 48.7 , 49.3 ) | -   ( - , - ) | 0.4   ( 0.2 , 0.5 ) | 0.2   ( -0.1 , 0.5 ) |
| Mexico | 35 | 296521 | 7763 | 0 | 7220 | 543 | -   ( - , - ) | 93   ( 93 , 93 ) | 6.9   ( 6.5 , 7.2 ) | -   ( - , - ) | 28.5  ( 28.3 , 28.8 ) | 27.8   ( 27.5 , 28.2 ) | -   ( - , - ) | 0.4   ( 0.2 , 0.6 ) | 0.3   ( -0.1 , 0.6 ) |
| Mexico | 36 | 724458 | 10283 | 0 | 7693 | 2590 | -   ( - , - ) | 73.26   ( 72.7 , 73.8 ) | 18.1   ( 16.6 , 19.6 ) | -    ( - , - ) | 12.8   ( 12.4 , 13.3 ) | 26.7   ( 25.1 , 28.2 ) | -   ( - , - ) | 0.4   ( -0.1 , 0.9 ) | 0.9   ( -0.7 , 2.4 ) |
| Mexico | 37 | 1868384 | 10687 | 321 | 7831 | 2535 | 3   ( 2.6 , 3.3 ) | 72.12   ( 71.7 , 72.6 ) | 18.4   ( 16.9 , 19.8 ) | 2.2   ( 2 , 2.5 ) | 5.1   ( 4.7 , 5.5 ) | 9   ( 7.6 , 10.5 ) | 0.2   ( -0.1 , 0.5 ) | 0.4   ( 0 , 0.9 ) | 1.0  ( -0.5 , 2.4 ) |
| Mexico | 38 | 5013466 | 13437 | 880 | 9650 | 2907 | 6.5   ( 6.3 , 6.7 ) | 71.37   ( 71 , 71.7 ) | 17.1   ( 15.6 , 18.5 ) | 3.5   ( 3.1 , 3.9 ) | 2.3   ( 1.9 , 2.6 ) | 4.1   ( 2.5 , 5.6 ) | 0.6   ( 0.3 , 1 ) | 0.6   ( 0.2 , 0.9 ) | 1.3   ( -0.2 , 2.7 ) |
| Mexico | 39 | 6644020 | 11822 | 1088 | 8892 | 1842 | 9.1   ( 8.9 , 9.3 ) | 75.06   ( 74.9 , 75.2 ) | 12.5   ( 10.6 , 14.4 ) | 2.9   ( 2.5 , 3.3 ) | 1.6   ( 1.2 , 1.9 ) | 2.3   ( 0.5 , 4.1 ) | 0.9   ( 0.5 , 1.4 ) | 0.7   ( 0.4 , 1 ) | 1.2   ( -0.7 , 3 ) |
| Mexico | 40 | 6737206 | 13172 | 1958 | 9721 | 1493 | 14.9   ( 14.7 , 15 ) | 73.36   ( 73.3 , 73.5 ) | 12.6   ( 12.4 , 12.8 ) | 3.6   ( 3.2 , 4.1 ) | 1.7   ( 1.3 , 2 ) | 3.1   ( 2.8 , 3.4 ) | 2.5   ( 2.1 , 2.9 ) | 1.4   ( 1 , 1.7 ) | 2.6   ( 2.3 , 2.9 ) |
| Mexico | 41 | 1383786 | 4254 | 966 | 2900 | 388 | 22.5   ( 22.3 , 22.8 ) | 67.95   ( 67.9 , 68 ) | 10.1   ( 9.8 , 10.3 ) | 5.7   ( 5.2 , 6.1 ) | 2.5  ( 2.1 , 2.9 ) | 4.5   ( 4.2 , 4.9 ) | 4.1   ( 3.7 , 4.5 ) | 2.2   ( 1.8 , 2.5 ) | 3.9   ( 3.6 , 4.2 ) |
| Mexico | >=42 | 268586 | 2616 | 761 | 1653 | 202 | 29.3   ( 29.2 , 29.4 ) | 62.1   ( 62 , 62.2 ) | 8.8   ( 8.3 , 9.4 ) | 11.4   ( 11 , 11.8 ) | 8.3  ( 7.9 , 8.7 ) | 14.7   ( 14 , 15.3 ) | 11.4   ( 11 , 11.8 ) | 8.3   ( 7.9 , 8.7 ) | 14.7   ( 14 , 15.3 ) |

| The Netherlands | 24 | 966 | 1664 | 276 | 1278 | 110 | 16.1   ( 15.7 , 16.5 ) | 76.86   ( 76.8 , 76.9 ) | 6.2   ( 5.5 , 7 ) | 867.2   ( 867.1 , 867.4 ) | 601.7   ( 601.5 , 601.9 ) | 494.6   ( 493.9 , 495.4 ) | 3.9   ( 3.2 , 4.6 ) | 0.9   ( 0.6 , 1.3 ) | 0.2   ( -0.6 , 1 ) |
| --- | --- | --- | --- | --- | --- | --- | --- | --- | --- | --- | --- | --- | --- | --- | --- |
| The Netherlands | 25 | 1133 | 503 | 150 | 327 | 26 | 29.3   ( 28.8 , 29.8 ) | 64.23   ( 64 , 64.5 ) | 4.5   ( 3.5 , 5.5 ) | 709.1   ( 708.8 , 709.4 ) | 258.5   ( 258.1 , 258.8 ) | 120   ( 118.9 , 121.1 ) | 2.1   ( 1.7 , 2.6 ) | 0.2   ( -0.3 , 0.8 ) | 0.1   ( -1.1 , 1.2 ) |
| The Netherlands | 26 | 1649 | 346 | 154 | 181 | 11 | 40.4   ( 39.5 , 41.2 ) | 52.97   ( 52.6 , 53.3 ) | 4.1   ( 3.4 , 4.9 ) | 416.5   ( 415.5 , 417.4 ) | 123.6   ( 123.3 , 123.9 ) | 73   ( 72.5 , 73.6 ) | 2   ( 0.8 , 3.2 ) | 0.1   ( -0.2 , 0.5 ) | 0   ( -0.6 , 0.7 ) |
| The Netherlands | 27 | 1782 | 310 | 143 | 149 | 18 | 45.4   ( 45 , 45.7 ) | 47.76   ( 47.5 , 48 ) | 6.6   ( 5.7 , 7.5 ) | 390   ( 389.5 , 390.5 ) | 102.2   ( 101.7 , 102.6 ) | 63.7   ( 62.6 , 64.8 ) | 2   ( 1.4 , 2.6 ) | 0.1   ( -0.4 , 0.6 ) | 0   ( -1.1 , 1.1 ) |
| The Netherlands | 28 | 2266 | 266 | 114 | 126 | 26 | 41.8   ( 41.3 , 42.2 ) | 46.71   ( 46.4 , 47 ) | 9.1   ( 7.7 , 10.6 ) | 297.1    ( 296.6 , 297.6 ) | 66.5   ( 65.8 , 67.1 ) | 72.3   ( 71.1 , 73.6 ) | 1.6   ( 1 , 2.2 ) | 0.1   ( -0.5 , 0.7 ) | 0.1   ( -1.3 , 1.4 ) |
| The Netherlands | 29 | 2640 | 199 | 80 | 101 | 18 | 37.2   ( 36.4 , 38 ) | 49   ( 48.4 , 49.6 ) | 10.5   ( 9.9 , 11.2 ) | 215.1    ( 214.4 , 215.9 ) | 45.2   ( 44.4 , 46.1 ) | 53.5   ( 52.4 , 54.6 ) | 1.1   ( 0.1 , 2.1 ) | 0.1   ( -0.7 , 0.9 ) | 0   ( -0.7 , 0.8 ) |
| The Netherlands | 30 | 3485 | 211 | 80 | 106 | 25 | 36.9   ( 36.4 , 37.4 ) | 45.93   ( 45.2 , 46.6 ) | 11.3   ( 9.9 , 12.6 ) | 174.6   ( 173.8 , 175.5 ) | 32.2  ( 31 , 33.5 ) | 50   ( 49.1 , 50.9 ) | 1.1   ( 0.2 , 1.9 ) | 0.1   ( -1.2 , 1.3 ) | 0.1   ( -1 , 1.1 ) |
| The Netherlands | 31 | 4519 | 214 | 62 | 121 | 31 | 27.8   ( 27 , 28.7 ) | 54.63   ( 54.2 , 55 ) | 14.1   ( 13.3 , 14.8 ) | 130.9   ( 130.2 , 131.7 ) | 30.0  ( 29.1 , 30.9 ) | 50.9   ( 50 , 51.7 ) | 0.8   ( 0 , 1.7 ) | 0.1   ( -0.8 , 1 ) | 0.1   ( -0.8 , 0.9 ) |
| The Netherlands | 32 | 6645 | 236 | 64 | 141 | 31 | 23.9   ( 23 , 24.7 ) | 59.83   ( 59.5 , 60.2 ) | 12.1   ( 10.8 , 13.5 ) | 100   ( 99 , 101 ) | 24.9   ( 24.7 , 25.3 ) | 39.9   ( 38.7 , 41.1 ) | 0.8   ( -0.3 , 2 ) | 0.1   ( -0.2 , 0.4 ) | 0.1   ( -1.2 , 1.4 ) |
| The Netherlands | 33 | 9771 | 236 | 56 | 159 | 21 | 22   ( 21.3 , 22.7 ) | 66.87   ( 66.6 , 67.1 ) | 12.3   ( 11.5 , 13 ) | 91.7   ( 90.9 , 92.4 ) | 18.4   ( 17.9 , 18.8 ) | 27.3   ( 26.6 , 28 ) | 0.8   ( -0.1 , 1.7 ) | 0.1   ( -0.3 , 0.6 ) | 0.1   ( -0.5 , 0.6 ) |
| The Netherlands | 34 | 16221 | 239 | 35 | 183 | 21 | 12.7   ( 11.6 , 13.9 ) | 76.08   ( 75.9 , 76.3 ) | 8.7   ( 7.6 , 9.9 ) | 23.3   ( 22 , 24.5 ) | 13.1   ( 12.5 , 13.7 ) | 13.7   ( 12.9 , 14.4 ) | 0.5   ( -0.8 , 1.7 ) | 0.1   ( -0.4 , 0.7 ) | 0   ( -0.9 , 1 ) |
| The Netherlands | 35 | 25112 | 286 | 68 | 190 | 28 | 24.3   ( 23.8 , 24.9 ) | 66.5   ( 66.2 , 66.8 ) | 9.6   ( 8.5 , 10.8 ) | 33.4   ( 32.7 , 34.1 ) | 9.1  ( 8.7 , 9.6 ) | 9.8   ( 8.8 , 10.8 ) | 1.1   ( 0.5 , 1.8 ) | 0.1   ( -0.3 , 0.6 ) | 0.1   ( -0.9 , 1 ) |
| The Netherlands | 36 | 50657 | 317 | 75 | 223 | 19 | 23.1   ( 22.5 , 23.7 ) | 68.81   ( 68.5 , 69.1 ) | 6.4   ( 5.5 , 7.3 ) | 15.7   ( 15.1 , 16.2 ) | 5.4   ( 4.8 , 6 ) | 3   ( 2.1 , 3.8 ) | 1.2   ( 0.6 , 1.7 ) | 0.2   ( -0.4 , 0.8 ) | 0   ( -0.8 , 0.9 ) |
| The Netherlands | 37 | 138122 | 351 | 107 | 206 | 38 | 28.1   ( 27.4 , 28.9 ) | 58.46   ( 58.2 , 58.7 ) | 9.7   ( 8.4 , 11 ) | 8.8   ( 7.9 , 9.8 ) | 1.9   ( 1.5 , 2.4 ) | 1.4   ( 0.2 , 2.6 ) | 1.7   ( 0.8 , 2.6 ) | 0.2   ( -0.2 , 0.5 ) | 0.1   ( -1.1 , 1.3 ) |
| The Netherlands | 38 | 287684 | 376 | 91 | 242 | 43 | 23.6   ( 23.1 , 24.1 ) | 63.64   ( 63.3 , 63.9 ) | 10.3   ( 9.3 , 11.2 ) | 7.4   ( 6.7 , 8 ) | 1.1   ( 0.6 , 1.7 ) | 0.5   ( -0.6 , 1.7 ) | 1.9   ( 1.3 , 2.5 ) | 0.2   ( -0.3 , 0.7 ) | 0.1   ( -1 , 1.2 ) |
| The Netherlands | 39 | 456134 | 448 | 90 | 292 | 66 | 19   ( 18.2 , 19.8 ) | 64.33   ( 64 , 64.6 ) | 13.3   ( 12.4 , 14.2 ) | 6.8   ( 6 , 7.7 ) | 0.8   ( 0.4 , 1.3 ) | 0.5   ( -0.5 , 1.5 ) | 2.5   ( 1.7 , 3.3 ) | 0.3   ( -0.1 , 0.8 ) | 0.2   ( -0.8 , 1.2 ) |
| The Netherlands | 40 | 504212 | 447 | 77 | 303 | 67 | 16.6   ( 16 , 17.2 ) | 67.71   ( 67.5 , 68 ) | 15   ( 14.2 , 15.8 ) | 5.9   ( 5.1 , 6.6 ) | 0.8   ( 0.4 , 1.2 ) | 0.5   ( -0.4 , 1.5 ) | 3.4   ( 2.6 , 4.1 ) | 0.5   ( 0.1 , 0.9 ) | 0.3   ( -0.6 , 1.2 ) |
| The Netherlands | 41 | 304486 | 382 | 61 | 254 | 67 | 14.7   ( 13.9 , 15.5 ) | 66.03   ( 65.8 , 66.3 ) | 16.8   ( 16.1 , 17.5 ) | 6.7   ( 5.9 , 7.5 ) | 1.2   ( 0.7 , 1.6 ) | 0.8   ( 0.1 , 1.5 ) | 6   ( 5.2 , 6.8 ) | 1.1   ( 0.7 , 1.5 ) | 0.7   ( 0 , 1.4 ) |
| The Netherlands | >=42 | 25806 | 301 | 54 | 201 | 46 | 15.9   ( 14.8 , 16.9 ) | 65.83   ( 65.5 , 66.1 ) | 14.8   ( 14 , 15.5 ) | 51.1   ( 50 , 52.3 ) | 10.7   ( 10.2 , 11.1 ) | 6.7   ( 5.9 , 7.6 ) | 51.1   ( 50 , 52.3 ) | 10.7   ( 10.2 , 11.1 ) | 6.7   ( 5.9 , 7.6 ) |

| Qatar | 24 | 86 | 18 | 5 | 13 | 0 | 27.5   ( 26.4 , 28.6 ) | 74.77   ( 74.2 , 75.3 ) | -   ( - , - ) | 630   ( 629.2 , 630.7 ) | 159.7   ( 158.7 , 160.6 ) | -   ( - , - ) | 0.9   ( -0.3 , 2.1 ) | 0.2   ( -0.5 , 0.8 ) | -   ( - , - ) |
| --- | --- | --- | --- | --- | --- | --- | --- | --- | --- | --- | --- | --- | --- | --- | --- |
| Qatar | 25 | 85 | 28 | 12 | 14 | 2 | 46.4   ( 46.2 , 46.7 ) | 53.73   ( 52.8 , 54.6 ) | 12.9   ( 12.2 , 13.6 ) | 780.9    ( 780.3 , 781.5 ) | 158.8   ( 157.7 , 159.9 ) | 235.7   ( 234.7 , 236.7 ) | 2.4   ( 2 , 2.8 ) | 0.2   ( -0.8 , 1.1 ) | 0.3   ( 0.2 , 0.4 ) |
| Qatar | 26 | 114 | 22 | 14 | 6 | 2 | 62.2   ( 61.5 , 63 ) | 35.86   ( 35.4 , 36.4 ) | 26.7   ( 25 , 28.5 ) | 715.8   ( 715.2 , 716.4 ) | 105.3   ( 105.2 , 105.3 ) | 182.6   ( 182.3 , 182.8 ) | 1.8   ( 0.3 , 3.3 ) | 0.2   ( 0.1 , 0.2 ) | 0.3   ( 0.2 , 0.4 ) |
| Qatar | 27 | 118 | 26 | 5 | 16 | 5 | 36.8   ( 35.1 , 38.6 ) | 65.25   ( 64.7 , 65.7 ) | 25.8   ( 25.1 , 26.5 ) | 312.8   ( 311.5 , 314.2 ) | 207.5  ( 207.1 , 207.9 ) | 378   ( 377.6 , 378.3 ) | 0.9   ( -0.4 , 2.2 ) | 0.3   ( 0 , 0.5 ) | 0.7   ( 0.3 , 1.2 ) |
| Qatar | 28 | 136 | 20 | 9 | 9 | 2 | 54.3   ( 53.8 , 54.8 ) | 44.72   ( 44.2 , 45.2 ) | 50   ( - , - ) | 643.7   ( 643.2 , 644.1 ) | 89.8   ( 89 , 90.7 ) | 133.3   ( - , - ) | 1.8   ( 1.1 , 2.6 ) | 0.1   ( -0.2 , 0.5 ) | 0.6   ( - , - ) |
| Qatar | 29 | 180 | 28 | 8 | 7 | 13 | 48.5   ( 47.9 , 49.2 ) | 27.67   ( 26.2 , 29.1 ) | 49.2   ( 47.7 , 50.7 ) | 493.2   ( 492.1 , 494.4 ) | 48.3   ( 47.6 , 49 ) | 284.4   ( 282.1 , 286.7 ) | 1.5   ( -0.1 , 3 ) | 0.1   ( -0.6 , 0.8 ) | 1.4   ( -0.9 , 3.7 ) |
| Qatar | 30 | 269 | 20 | 8 | 10 | 2 | 39.4   ( 38.8 , 40 ) | 49.66   ( 49.4 , 49.9 ) | 14.3   ( 14.3 , 14.3 ) | 308   ( 307.1 , 308.9 ) | 64.3   ( 63.8 , 64.9 ) | 83.3   ( 82.5 , 84.1 ) | 1.6   ( 1.2 , 2.1 ) | 0.2   ( -0.2 , 0.5 ) | 0.3   ( 0.2 , 0.4 ) |
| Qatar | 31 | 365 | 21 | 9 | 11 | 1 | 52.5   ( 51.2 , 53.9 ) | 46.42   ( 45.7 , 47.2 ) | 10   ( - , - ) | 180.8   ( 179.6 , 182 ) | 40.3   ( 38.3 , 42.3 ) | 62.5   ( - , - ) | 1.3   ( 0.2 , 2.4 ) | 0.1   ( -1.8 , 2.1 ) | 0.3   ( - , - ) |
| Qatar | 32 | 455 | 27 | 10 | 12 | 5 | 37   ( 35 , 39 ) | 36.06   ( 34.3 , 37.9 ) | 18.4   ( 17.7 , 19.1 ) | 152.7   ( 151.4 , 154 ) | 34.8   ( 32.8 , 36.7 ) | 94.2   ( 93.2 , 95.1 ) | 1.2   ( -0.5 , 2.9 ) | 0.2   ( -1.8 , 2.1 ) | 0.5   ( -0.3 , 1.3 ) |
| Qatar | 33 | 698 | 26 | 10 | 15 | 1 | 33.4   ( 31.9 , 34.8 ) | 58.52   ( 57.8 , 59.2 ) | 100   ( - , - ) | 190.8   ( 189 , 192.6 ) | 29.8   ( 28.9 , 30.7 ) | 62.5   ( - , - ) | 1.7   ( 0 , 3.5 ) | 0.3   ( -0.5 , 1 ) | 0.3   ( - , - ) |
| Qatar | 34 | 1231 | 28 | 8 | 19 | 1 | 34.7   ( 33.4 , 36 ) | 55.84   ( 54.8 , 56.9 ) | 33.3   ( - , - ) | 116.4   ( 115.7 , 117 ) | 13.3   ( 11.6 , 15.1 ) | 31.2   ( - , - ) | 1.7   ( 1.3 , 2.1 ) | 0.2   ( -1.8 , 2.2 ) | 0.3   ( - , - ) |
| Qatar | 35 | 1840 | 32 | 19 | 12 | 1 | 59.7   ( 59.5 , 60 ) | 33.18   ( 32.4 , 33.9 ) | 20   ( - , - ) | 102.8   ( 102.1 , 103.5 ) | 6.9   ( 5.7 , 8.2 ) | 16.1   ( - , - ) | 3   ( 2.3 , 3.7 ) | 0.1   ( -1.2 , 1.5 ) | 0.3   ( - , - ) |
| Qatar | 36 | 3783 | 24 | 9 | 13 | 2 | 42.5   ( 41.1 , 44 ) | 67.51   ( 67.3 , 67.7 ) | 16   ( 14 , 18 ) | 21.4   ( 20 , 22.9 ) | 5.4  ( 4.1 , 6.6 ) | 7.7   ( 7.5 , 7.9 ) | 1.7   ( 0.2 , 3.2 ) | 0.2   ( -1.1 , 1.5 ) | 0.3   ( 0.3 , 0.3 ) |
| Qatar | 37 | 9295 | 28 | 9 | 15 | 4 | 30.2   ( 29.4 , 31.1 ) | 48.55   ( 47.9 , 49.2 ) | 19.5   ( 18.2 , 20.8 ) | 10.4   ( 9.4 , 11.4 ) | 1.9  ( 0.9 , 2.9 ) | 3.4   ( 2.5 , 4.4 ) | 1.5   ( 0.5 , 2.5 ) | 0.2   ( -0.9 , 1.3 ) | 0.4   ( -0.4 , 1.2 ) |
| Qatar | 38 | 20763 | 31 | 9 | 21 | 1 | 28.6   ( 27.5 , 29.6 ) | 63.89   ( 63.3 , 64.5 ) | 10   ( - , - ) | 6.8   ( 5.7 , 8 ) | 1.1   ( -0.1 , 2.3 ) | 1.2   ( - , - ) | 1.7   ( 0.6 , 2.8 ) | 0.3   ( -1 , 1.6 ) | 0.4   ( - , - ) |
| Qatar | 39 | 26731 | 28 | 9 | 18 | 1 | 41.2   ( 39.4 , 43 ) | 61.34   ( 60.8 , 61.9 ) | 50   ( - , - ) | 6.9   ( 5.7 , 8 ) | 0.8   ( -1.5 , 3.1 ) | 1.1   ( - , - ) | 3.1   ( 2.1 , 4.2 ) | 0.4   ( -2 , 2.7 ) | 0.5   ( - , - ) |
| Qatar | 40 | 20555 | 25 | 7 | 15 | 3 | 25.4   ( 23.7 , 27.2 ) | 54.22   ( 52.6 , 55.8 ) | 19.2   ( 18.8 , 19.6 ) | 6.3   ( 4.9 , 7.7 ) | 0.6   ( -1.5 , 2.7 ) | 2.2   ( 1.1 , 3.2 ) | 4.3   ( 3 , 5.6 ) | 0.4   ( -1.7 , 2.6 ) | 1.6   ( 0.5 , 2.7 ) |
| Qatar | 41 | 7007 | 13 | 3 | 8 | 2 | 50   ( 48.1 , 51.9 ) | 72.11   ( 71.4 , 72.8 ) | 50   ( - , - ) | 10.3   ( 9.1 , 11.5 ) | 1.8   ( 1.3 , 2.4 ) | 8.3   ( - , - ) | 8.8   ( 7.7 , 9.9 ) | 1.7   ( 1.2 , 2.3 ) | 7.9   ( - , - ) |
| Qatar | >=42 | 528 | 0 | 0 | 0 | 0 | -   ( - , - ) | -   ( - , - ) | -   ( - , - ) | -   ( - , - ) | -   ( - , - ) | -   ( - , - ) | -   ( - , - ) | -   ( - , - ) | -   ( - , - ) |

| Scotland | 24 | 628 | 335 | 94 | 211 | 30 | 26.9   ( 26.1 , 27.7 ) | 60.66   ( 60.2 , 61.1 ) | 12.9   ( 11.8 , 14 ) | 690.9   ( 690.3 , 691.5 ) | 258.5   ( 257.7 , 259.3 ) | 353.7   ( 352.8 , 354.7 ) | 1.4   ( 0.3 , 2.5 ) | 0.2   ( -0.9 , 1.3 ) | 0.2   ( -1 , 1.3 ) |
| --- | --- | --- | --- | --- | --- | --- | --- | --- | --- | --- | --- | --- | --- | --- | --- |
| Scotland | 25 | 667 | 272 | 94 | 161 | 17 | 32.9   ( 32.2 , 33.5 ) | 57.16   ( 56.7 , 57.6 ) | 10.4   ( 9.3 , 11.4 ) | 752   ( 751.5 , 752.6 ) | 219.5   ( 218.7 , 220.3 ) | 248.8   ( 248.2 , 249.4 ) | 1.5   ( 0.6 , 2.4 ) | 0.2   ( -0.6 , 0.9 ) | 0.1   ( -0.8 , 1 ) |
| Scotland | 26 | 946 | 270 | 92 | 156 | 22 | 31.3   ( 30.6 , 32 ) | 56.92   ( 56.5 , 57.4 ) | 10   ( 8.9 , 11.2 ) | 520.8   ( 519.9 , 521.6 ) | 159.1   ( 158.3 , 159.9 ) | 197.6   ( 196.5 , 198.6 ) | 1.3   ( 0.4 , 2.3 ) | 0.2   ( -0.7 , 1.1 ) | 0.1   ( -0.8 , 1 ) |
| Scotland | 27 | 1218 | 247 | 99 | 126 | 22 | 37.5   ( 36.7 , 38.2 ) | 48.46   ( 47.8 , 49.1 ) | 13.9   ( 12.9 , 15 ) | 442.4   ( 441.5 , 443.3 ) | 108.83   ( 108 , 109.7 ) | 151.4   ( 150.6 , 152.2 ) | 1.5   ( 0.5 , 2.5 ) | 0.1   ( -0.8 , 1.1 ) | 0.1   ( -0.9 , 1.1 ) |
| Scotland | 28 | 1650 | 254 | 112 | 113 | 29 | 40.9   ( 39.9 , 41.9 ) | 42.02   ( 41.4 , 42.7 ) | 13.8   ( 12.9 , 14.6 ) | 335.6   ( 334.8 , 336.5 ) | 75.46   ( 74.5 , 76.4 ) | 130.7   ( 129.5 , 131.9 ) | 1.7   ( 0.5 , 2.9 ) | 0.1   ( -1 , 1.2 ) | 0.1   ( -0.8 , 1.1 ) |
| Scotland | 29 | 1873 | 198 | 63 | 120 | 15 | 29.7   ( 28.8 , 30.5 ) | 58.35   ( 57.9 , 58.8 ) | 14.5   ( 13.6 , 15.4 ) | 270.8   ( 269.8 , 271.9 ) | 69.04   ( 68.2 , 69.9 ) | 106.8   ( 105.9 , 107.7 ) | 1   ( -0.2 , 2.1 ) | 0.1   ( -0.8 , 1.1 ) | 0.1   ( -0.8 , 1 ) |
| Scotland | 30 | 2539 | 190 | 49 | 112 | 29 | 25.7   ( 24.9 , 26.5 ) | 55.67   ( 54.8 , 56.6 ) | 17.5   ( 16.6 , 18.4 ) | 181.7   ( 180.8 , 182.5 ) | 46.7   ( 45.6 , 47.8 ) | 92.2   ( 91.2 , 93.2 ) | 0.8   ( -0.2 , 1.8 ) | 0.1   ( -1 , 1.2 ) | 0.1   ( -1 , 1.2 ) |
| Scotland | 31 | 3186 | 203 | 44 | 133 | 26 | 20.8   ( 19.9 , 21.7 ) | 65.26   ( 64.9 , 65.7 ) | 18.8   ( 18 , 19.6 ) | 138.7   ( 137.6 , 139.8 ) | 46.13   ( 45.4 , 46.9 ) | 103.6   ( 102.5 , 104.6 ) | 0.7   ( -0.2 , 1.5 ) | 0.2   ( -0.6 , 0.9 ) | 0.2   ( -1 , 1.4 ) |
| Scotland | 32 | 4847 | 235 | 66 | 155 | 14 | 25   ( 23.9 , 26.1 ) | 66.19   ( 65.7 , 66.6 ) | 10.2   ( 9.3 , 11 ) | 131.4   ( 130.2 , 132.6 ) | 35.94   ( 35.4 , 36.5 ) | 48.3   ( 47.7 , 48.9 ) | 1   ( -0.3 , 2.4 ) | 0.2   ( -0.4 , 0.8 ) | 0.1   ( -0.5 , 0.7 ) |
| Scotland | 33 | 6558 | 227 | 49 | 162 | 16 | 21.9   ( 21.2 , 22.7 ) | 71.89   ( 71.5 , 72.3 ) | 13.9   ( 13 , 14.9 ) | 118.1   ( 117.1 , 119.2 ) | 26.79   ( 26.1 , 27.5 ) | 48.9   ( 47.6 , 50.2 ) | 0.9   ( -0.2 , 1.9 ) | 0.2   ( -0.5 , 0.9 ) | 0.1   ( -0.9 , 1.2 ) |
| Scotland | 34 | 10837 | 263 | 66 | 178 | 19 | 24.3   ( 23.5 , 25.1 ) | 70.09   ( 69.6 , 70.5 ) | 9.5   ( 8.6 , 10.5 ) | 70.3   ( 69.2 , 71.4 ) | 18.2   ( 17.3 , 19.1 ) | 24.6   ( 23.8 , 25.5 ) | 1.2   ( 0.2 , 2.2 ) | 0.2   ( -0.6 , 1 ) | 0.1   ( -0.7 , 1 ) |
| Scotland | 35 | 17435 | 312 | 85 | 193 | 34 | 24.6   ( 23.7 , 25.5 ) | 60.53   ( 60.1 , 61 ) | 14.2   ( 12.8 , 15.5 ) | 43.9   ( 42.8 , 45 ) | 13.06   ( 12.4 , 13.7 ) | 23.4   ( 22.2 , 24.6 ) | 1.4   ( 0.5 , 2.3 ) | 0.2   ( -0.4 , 0.9 ) | 0.2   ( -1 , 1.4 ) |
| Scotland | 36 | 32835 | 348 | 104 | 211 | 33 | 26.4   ( 25.4 , 27.3 ) | 60.71   ( 60.4 , 61.1 ) | 9.7   ( 8.9 , 10.6 ) | 25.1   ( 23.8 , 26.4 ) | 7.93   ( 7.2 , 8.7 ) | 7.7   ( 6.6 , 8.9 ) | 1.6   ( 0.4 , 2.9 ) | 0.3   ( -0.4 , 0.9 ) | 0.1   ( -1 , 1.3 ) |
| Scotland | 37 | 69197 | 353 | 90 | 214 | 49 | 24.4   ( 23.6 , 25.3 ) | 57.26   ( 56.8 , 57.8 ) | 13.2   ( 11.9 , 14.5 ) | 12.9   ( 12 , 13.8 ) | 3.81   ( 2.7 , 5 ) | 4.1   ( 2.8 , 5.5 ) | 1.7   ( 0.8 , 2.5 ) | 0.3   ( -0.7 , 1.2 ) | 0.2   ( -1 , 1.4 ) |
| Scotland | 38 | 143175 | 391 | 96 | 236 | 59 | 22.3   ( 21.4 , 23.2 ) | 60.12   ( 59.7 , 60.5 ) | 13.7   ( 12.6 , 14.7 ) | 9.7   ( 8.6 , 10.7 ) | 2.19   ( 1.6 , 2.7 ) | 1.7   ( 0.6 , 2.8 ) | 2   ( 1 , 2.9 ) | 0.3   ( -0.2 , 0.9 ) | 0.2    ( -0.8 , 1.3 ) |
| Scotland | 39 | 268428 | 356 | 74 | 234 | 48 | 19   ( 18 , 20 ) | 62.75   ( 62.1 , 63.4 ) | 13.7   ( 12.6 , 14.7 ) | 5.7   ( 4.6 , 6.8 ) | 1.09   ( 0 , 2.1 ) | 0.8   ( -0.5 , 2 ) | 1.9   ( 0.8 , 3 ) | 0.4   ( -0.6 , 1.3 ) | 0.2   ( -0.9 , 1.4 ) |
| Scotland | 40 | 304758 | 396 | 87 | 251 | 58 | 20.8   ( 19.9 , 21.6 ) | 63.14   ( 62.7 , 63.5 ) | 14.1   ( 12.9 , 15.2 ) | 6.2   ( 5.3 , 7.1 ) | 1.07   ( 0.5 , 1.7 ) | 0.8   ( -0.4 , 2.1 ) | 3.5   ( 2.6 , 4.5 ) | 0.6   ( 0 , 1.2 ) | 0.4   ( -0.8 , 1.7 ) |
| Scotland | 41 | 225926 | 248 | 40 | 164 | 44 | 16.2   ( 14.9 , 17.5 ) | 63.37   ( 62.7 , 64 ) | 18.5   ( 17.6 , 19.4 ) | 4.4   ( 3 , 5.7 ) | 0.91   ( -0.2 , 2 ) | 0.8   ( -0.2 , 1.8 ) | 3.8   ( 2.4 , 5.2 ) | 0.8   ( -0.3 , 1.9 ) | 0.7   ( -0.3 , 1.7 ) |
| Scotland | >=42 | 27349 | 26 | 7 | 16 | 3 | 61.8   ( 60.9 , 62.6 ) | 70.02   ( 69.1 , 70.9 ) | 55   ( 53.9 , 56.1 ) | 15.5   ( 14.6 , 16.5 ) | 1.17   ( 0.3 , 2.1 ) | 3.3   ( 3.1 , 3.5 ) | 15.5   ( 14.6 , 16.5 ) | 1.2   ( 0.3 , 2.1 ) | 3.3   ( 3.1 , 3.5 ) |

| Sweden | 24 | 889 | 142 | 62 | 80 | 0 | 50   ( 49.1 , 51 ) | 70.2  ( 69.6 , 70.8 ) | -   ( - , - ) | 725.2   ( 724.5 , 725.9 ) | 159.6   ( 158.9 , 160.3 ) | -   ( - , - ) | 1.7   ( 1.1 , 2.3 ) | 0.1   ( -0.7 , 0.9 ) | -   ( - , - ) |
| --- | --- | --- | --- | --- | --- | --- | --- | --- | --- | --- | --- | --- | --- | --- | --- |
| Sweden | 25 | 1139 | 93 | 59 | 34 | 0 | 67.3   ( 66.4 , 68.1 ) | 53.9  ( 53.6 , 54.1 ) | -   ( - , - ) | 507.4   ( 506.3 , 508.4 ) | 102.6   ( 102 , 103.2 ) | -   ( - , - ) | 1.3   ( 1 , 1.7 ) | 0.1   ( -0.7 , 0.8 ) | -   ( - , - ) |
| Sweden | 26 | 1380 | 85 | 38 | 47 | 0 | 83.3   ( 82.5 , 84.1 ) | 92.9  ( 92.6 , 93.3 ) | -   ( - , - ) | 543.5   ( 542.8 , 544.2 ) | 90.6  ( 90 , 91.2 ) | -   ( - , - ) | 1.7   ( 1.4 , 2.1 ) | 0.1   ( -0.6 , 0.7 ) | -   ( - , - ) |
| Sweden | 27 | 1666 | 79 | 21 | 58 | 0 | 56.9   ( 56.1 , 57.7 ) | 80.4  ( 79.7 , 81 ) | -   ( - , - ) | 356.5   ( 356.3 , 356.8 ) | 78.3  ( 77.8 , 78.7 ) | -   ( - , - ) | 1.2   ( 1 , 1.3 ) | 0.1   ( -0.3 , 0.5 ) | -   ( - , - ) |
| Sweden | 28 | 1975 | 91 | 22 | 69 | 0 | 79.4   ( 78.6 , 80.2 ) | 92.6  ( 92.1 , 93 ) | -   ( - , - ) | 340.5   ( 339.8 , 341.2 ) | 80.8   ( 80.3 , 81.4 ) | -   ( - , - ) | 1.8   ( 1 , 2.6 ) | 0.1   ( -0.5 , 0.6 ) | -   ( - , - ) |
| Sweden | 29 | 2633 | 117 | 18 | 99 | 0 | 47.5   ( 47.4 , 47.6 ) | 86.2  ( 85.6 , 86.7 ) | -   ( - , - ) | 326.8   ( 326.2 , 327.3 ) | 62.4   ( 61.8 , 62.9 ) | -   ( - , - ) | 1.4   ( 0.9 , 1.8 ) | 0.1   ( -0.4 , 0.6 ) | -   ( - , - ) |
| Sweden | 30 | 3281 | 179 | 23 | 144 | 12 | 46.7   ( 45.6 , 47.7 ) | 85.7  ( 85.2 , 86.2 ) | 45.2   ( 44.7 , 45.7 ) | 249.4   ( 249.1 , 249.6 ) | 55.8   ( 55.1 , 56.5 ) | 343   ( 342.9 , 343.1 ) | 1.6   ( 1.3 , 1.8 ) | 0.1   ( -0.7 , 0.9 ) | 0.2   ( 0.2 , 0.2 ) |
| Sweden | 31 | 4314 | 161 | 21 | 134 | 6 | 36.8   ( 36.5 , 37.1 ) | 84.7  ( 84.2 , 85.2 ) | 54.5   ( - , - ) | 191.9   ( 191.7 , 192.1 ) | 44.0   ( 43.5 , 44.5 ) | 230.8   ( - , - ) | 1.4   ( 0.9 , 1.8 ) | 0.1   ( -0.4 , 0.6 ) | 0.2   ( - , - ) |
| Sweden | 32 | 6299 | 170 | 16 | 153 | 1 | 41.1   ( 40.7 , 41.5 ) | 91.4  ( 90.9 , 91.8 ) | 7.7   ( - , - ) | 178.6   ( 178 , 179.2 ) | 28.6   ( 27.9 , 29.2 ) | 40   ( - , - ) | 1.4   ( 1 , 1.8 ) | 0.1   ( -0.5 , 0.8 ) | 0   ( - , - ) |
| Sweden | 33 | 9528 | 236 | 30 | 190 | 16 | 35.9   ( 35.4 , 36.4 ) | 83.1   ( 82.6 , 83.5 ) | 32.1   ( 31.8 , 32.5 ) | 163.4   ( 162.6 , 164.2 ) | 22.8  ( 22.3 , 23.5 ) | 137.6   ( 137.5 , 137.8 ) | 1.4   ( 0.8 , 2.1 ) | 0.1   ( -0.5 , 0.8 ) | 0.2   ( -0.1 , 0.5 ) |
| Sweden | 34 | 15860 | 194 | 40 | 144 | 10 | 49.2   ( 48 , 50.3 ) | 81.5  ( 80.9 , 82 ) | 25.8   ( 25.1 , 26.5 ) | 80.2   ( 79.8 , 80.5 ) | 12.9  ( 12.2 , 13.7 ) | 59.1   ( 58.8 , 59.3 ) | 1.5   ( 1.2 , 1.8 ) | 0.1   ( -0.7 , 0.9 ) | 0.2   ( 0.1 , 0.3 ) |
| Sweden | 35 | 25114 | 285 | 82 | 187 | 16 | 46.7   ( 46 , 47.4 ) | 70.1  ( 69.5 , 70.8 ) | 28.6   ( 27.6 , 29.5 ) | 62.1    ( 61.5 , 62.7 ) | 9.8   ( 8.9 , 10.7 ) | 44.5   ( 44.3 , 44.8 ) | 1.9   ( 1.2 , 2.6 ) | 0.1   ( -0.7 , 1 ) | 0.2   ( 0 , 0.4 ) |
| Sweden | 36 | 47289 | 323 | 81 | 212 | 30 | 35.8   ( 35.3 , 36.3 ) | 68.3  ( 67.8 , 68.8 ) | 23.7    ( 22.2 , 25.2 ) | 29.3    ( 28.9 , 29.8 ) | 5.8  ( 5.3 , 6.4 ) | 13.5   ( 12.2 , 14.9 ) | 1.7   ( 1.2 , 2.1 ) | 0.2   ( -0.4 , 0.7 ) | 0.2   ( -1.3 , 1.6 ) |
| Sweden | 37 | 110994 | 408 | 90 | 293 | 25 | 30.9   ( 30.3 , 31.6 ) | 72.5   ( 72 , 72.9 ) | 22.4   ( 21.7 , 23.2 ) | 18   ( 17.3 , 18.7 ) | 3.3   ( 2.7 , 4 ) | 5.1   ( 5 , 5.2 ) | 2   ( 1.4 , 2.6 ) | 0.2   ( -0.4 , 0.8 ) | 0.2   ( 0 , 0.4 ) |
| Sweden | 38 | 292094 | 511 | 45 | 380 | 86 | 19.8   ( 19.4 , 20.1 ) | 74.4  ( 74 , 74.8 ) | 22.3   ( 21.8 , 22.8 ) | 9.2   ( 8.8 , 9.6 ) | 1.7  ( 1.2 , 2.2 ) | 1.9   ( 1.4 , 2.3 ) | 1.9   ( 1.5 , 2.3 ) | 0.3   ( -0.2 , 0.8 ) | 0.2   ( -0.2 , 0.7 ) |
| Sweden | 39 | 485699 | 512 | 41 | 386 | 85 | 23.6   ( 23.2 , 23.9 ) | 76.2  ( 75.9 , 76.5 ) | 21.9   ( 21.4 , 22.4 ) | 8.7   ( 7.9 , 9.5 ) | 1.1  ( 0.5 , 1.6 ) | 1.1   ( 0.7 , 1.4 ) | 2.8   ( 2 , 3.7 ) | 0.3   ( -0.2 , 0.9 ) | 0.3   ( -0.1 , 0.7 ) |
| Sweden | 40 | 577656 | 530 | 35 | 400 | 95 | 16.5   ( 16.3 , 16.7 ) | 77.9  ( 77.5 , 78.3 ) | 24.9   ( 24.3 , 25.4 ) | 6.3   ( 5.8 , 6.8 ) | 0.9  ( 0.4 , 1.5 ) | 1   ( 0.5 , 1.5 ) | 3.4   ( 2.8 , 3.9 ) | 0.5   ( 0 , 1 ) | 0.5   ( 0 , 1 ) |
| Sweden | 41 | 375626 | 379 | 69 | 284 | 26 | 28.1   ( 27.6 , 28.5 ) | 74.0  ( 73.6 , 74.5 ) | 25   ( 24.3 , 25.7 ) | 10.3   ( 9.8 , 10.7 ) | 1.1  ( 0.3 , 1.8 ) | 0.9   ( 0.6 , 1.2 ) | 7.3   ( 6.9 , 7.7 ) | 0.8   ( 0 , 1.5 ) | 0.7   ( 0.4 , 1 ) |
| Sweden | >=42 | 138938 | 169 | 16 | 147 | 6 | 54.8   ( 53.8 , 55.9 ) | 90.6  ( 90.2 , 91 ) | 46.2   ( - , - ) | 24.3   ( 24.2 , 24.5 ) | 1.8  ( 1.1 , 2.4 ) | 3   ( - , - ) | 24.3   ( 24.2 , 24.5 ) | 1.8   ( 1.1 , 2.4 ) | 3   ( - , - ) |

| USA | 24 | 78757 | 17790 | 6555 | 9142 | 2093 | 36.8   ( 36.6 , 37 ) | 51.3  ( 51.1 , 51.4 ) | 11.6   ( 11.4 , 11.9 ) | 510.1   ( 510 , 510.2 ) | 125.8   ( 125.8 , 126 ) | 185.9   ( 185.7 , 186.1 ) | 1.7   ( 1.4 , 1.9 ) | 0.2   ( 0 , 0.3 ) | 0.1   ( -0.1 , 0.4 ) |
| --- | --- | --- | --- | --- | --- | --- | --- | --- | --- | --- | --- | --- | --- | --- | --- |
| USA | 25 | 85543 | 14249 | 5932 | 6761 | 1556 | 41.6   ( 41.5 , 41.7 ) | 47.4  ( 47.3 , 47.5 ) | 10.7   ( 10.4 , 11.1 ) | 394.1   ( 394 , 394.3 ) | 93.7   ( 93.6 , 93.9 ) | 121.6   ( 121.4 , 121.8 ) | 1.5   ( 1.3 , 1.7 ) | 0.1   ( 0 , 0.3 ) | 0.1   ( -0.2 , 0.4 ) |
| USA | 26 | 96418 | 14156 | 5651 | 7026 | 1479 | 39.9   ( 39.8 , 40 ) | 49.6   ( 49.5 , 49.7 ) | 10.4   ( 10.1 , 10.6 ) | 350.8   ( 350.7 , 350.9 ) | 88.7   ( 88.5 , 88.8 ) | 96.9   ( 96.7 , 97.1 ) | 1.5   ( 1.3 , 1.6 ) | 0.1   ( 0 , 0.2 ) | 0.1   ( -0.1 , 0.3 ) |
| USA | 27 | 110030 | 12974 | 5329 | 6300 | 1345 | 41   ( 40.9 , 41.1 ) | 48.5  ( 48.4 , 48.6 ) | 10.3   ( 10 , 10.6 ) | 300.6   ( 300.5 , 300.7 ) | 72.0  ( 71.9 , 72.1 ) | 74.7   ( 74.5 , 75 ) | 1.4   ( 1.2 , 1.6 ) | 0.1   ( 0 , 0.2 ) | 0.1   ( -0.2 , 0.3 ) |
| USA | 28 | 136450 | 14347 | 5525 | 7130 | 1692 | 38.5   ( 38.4 , 38.6 ) | 49.7  ( 49.6 , 49.7 ) | 11.7   ( 11.6 , 11.9 ) | 286.6   ( 286.5 , 286.6 ) | 67.2   ( 67.1 , 67.4 ) | 66.3   ( 66.2 , 66.5 ) | 1.4   ( 1.3 , 1.6 ) | 0.1   ( 0 , 0.3 ) | 0.1   ( -0.1 , 0.3 ) |
| USA | 29 | 152537 | 11426 | 4161 | 5963 | 1302 | 36.3   ( 36.2 , 36.5 ) | 52.1   ( 52 , 52.2 ) | 11.4   ( 11.3 , 11.5 ) | 223   ( 222.9 , 223.1 ) | 49.9   ( 49.8 , 50.1 ) | 50.7   ( 50.4 , 51 ) | 1.1   ( 1 , 1.2 ) | 0.1   ( 0 , 0.2 ) | 0.1   ( -0.1 , 0.2 ) |
| USA | 30 | 206958 | 12995 | 4231 | 7083 | 1681 | 32.5   ( 32.3 , 32.6 ) | 54.5  ( 54.4 , 54.6 ) | 12.9   ( 12.7 , 13.1 ) | 198.9   ( 198.8 , 199 ) | 44.8   ( 44.6 , 44.9 ) | 41.9   ( 41.5 , 42.3 ) | 1.1   ( 1 , 1.2 ) | 0.1   ( 0 , 0.2 ) | 0.1   ( -0.1 , 0.3 ) |
| USA | 31 | 262218 | 12441 | 3987 | 7019 | 1435 | 31.9   ( 31.8 , 32.1 ) | 56.4  ( 56.3 , 56.5 ) | 11.4   ( 11.2 , 11.7 ) | 160.4   ( 160.3 , 160.5 ) | 34.6  ( 34.4 , 34.7 ) | 30.9   ( 30.4 , 31.3 ) | 1   ( 0.9 , 1.2 ) | 0.1   ( 0 , 0.3 ) | 0.1   ( -0.2 , 0.4 ) |
| USA | 32 | 400907 | 15012 | 4404 | 8845 | 1763 | 29.3   ( 29.2 , 29.4 ) | 58.9   ( 58.9 , 59 ) | 11.6   ( 11.4 , 11.9 ) | 134   ( 133.9 , 134.1 ) | 27.9   ( 27.8 , 28 ) | 27.1   ( 26.6 , 27.5 ) | 1.2   ( 1.1 , 1.3 ) | 0.1   ( 0.1 , 0.2 ) | 0.1   ( -0.1 , 0.4 ) |
| USA | 33 | 563577 | 14199 | 3573 | 9204 | 1422 | 25.1   ( 24.9 , 25.2 ) | 64.8  ( 64.7 , 64.9 ) | 9.9   ( 9.7 , 10.2 ) | 120.6   ( 120.5 , 120.8 ) | 19.9   ( 19.8 , 20.1 ) | 16.6   ( 16.2 , 17 ) | 1   ( 0.8 , 1.1 ) | 0.2   ( 0 , 0.3 ) | 0.1   ( -0.2 , 0.3 ) |
| USA | 34 | 1023518 | 15987 | 4752 | 9651 | 1584 | 29.6   ( 29.5 , 29.8 ) | 60.3   ( 60.3 , 60.4 ) | 9.9   ( 9.7 , 10.1 ) | 63.3   ( 63.1 , 63.5 ) | 11.8  ( 11.7 , 11.9 ) | 11.1   ( 10.7 , 11.5 ) | 1.3   ( 1.1 , 1.4 ) | 0.2   ( 0 , 0.3 ) | 0.1   ( -0.1 , 0.3 ) |
| USA | 35 | 1603296 | 16983 | 5274 | 9827 | 1882 | 31   ( 30.9 , 31.1 ) | 57.8  ( 57.8 , 57.9 ) | 11   ( 10.8 , 11.2 ) | 44.3   ( 44.1 , 44.4 ) | 7.9   ( 7.8 , 8 ) | 7.5   ( 7 , 8 ) | 1.5   ( 1.3 , 1.6 ) | 0.2   ( 0.1 , 0.3 ) | 0.1   ( -0.2 , 0.4 ) |
| USA | 36 | 3198731 | 20331 | 6026 | 11604 | 2701 | 29.5   ( 29.3 , 29.7 ) | 57.1   ( 57 , 57.2 ) | 13.2   ( 13 , 13.4 ) | 25   ( 24.7 , 25.2 ) | 4.7   ( 4.6 , 4.9 ) | 5   ( 4.6 , 5.5 ) | 1.7   ( 1.6 , 1.8 ) | 0.2   ( 0.1 , 0.3 ) | 0.2   ( -0.1 , 0.5 ) |
| USA | 37 | 7054527 | 20889 | 5628 | 11898 | 3363 | 26.9   ( 26.7 , 27 ) | 56.9   ( 56.9 , 57.0 ) | 16.1   ( 15.9 , 16.2 ) | 12.5   ( 12.1 , 12.9 ) | 2.3  ( 2.1 , 2.4 ) | 2.5   ( 2.3 , 2.8 ) | 1.7   ( 1.6 , 1.9 ) | 0.2   ( 0.1 , 0.3 ) | 0.2   ( 0 , 0.4 ) |
| USA | 38 | 14520393 | 21585 | 5040 | 12745 | 3800 | 23.2   ( 23 , 23.4 ) | 59.0  ( 59 , 59.1 ) | 17.6   ( 17.4 , 17.7 ) | 7.6   ( 7.3 , 7.8 ) | 1.2   ( 1 , 1.4 ) | 1.3   ( 0.8 , 1.7 ) | 1.8   ( 1.7 , 1.9 ) | 0.3   ( 0.2 , 0.4 ) | 0.3   ( 0.1 , 0.5 ) |
| USA | 39 | 26234497 | 17741 | 3958 | 10735 | 3048 | 22.2   ( 22.1 , 22.4 ) | 60.5  ( 60.4 , 60.6 ) | 17.1   ( 16.9 , 17.3 ) | 4.1   ( 3.8 , 4.4 ) | 0.6   ( 0.2 , 0.9 ) | 0.5   ( 0.2 , 0.9 ) | 1.8   ( 1.7 , 2 ) | 0.3   ( 0.1 , 0.4 ) | 0.3   ( 0.1 , 0.5 ) |
| USA | 40 | 18214984 | 12422 | 2810 | 7592 | 2020 | 22.5   ( 22.4 , 22.7 ) | 61.3  ( 61.2 , 61.4 ) | 16   ( 15.7 , 16.2 ) | 3.4   ( 3.1 , 3.6 ) | 0.5  ( 0.4 , 0.7 ) | 0.6   ( 0.3 , 0.9 ) | 2.4   ( 2.1 , 2.6 ) | 0.4   ( 0.3 , 0.6 ) | 0.4   ( 0.2 , 0.7 ) |
| USA | 41 | 5500130 | 3777 | 991 | 2213 | 573 | 25.9   ( 25.6 , 26.2 ) | 58.7  ( 58.6 , 58.9 ) | 14.8   ( 14.5 , 15.1 ) | 3.5   ( 3.2 , 3.9 ) | 0.5  ( 0.3 , 0.8 ) | 0.5   ( 0.1 , 1 ) | 2.9   ( 2.4 , 3.3 ) | 0.5   ( 0.3 , 0.7 ) | 0.5   ( 0.1 , 0.9 ) |
| USA | >=42 | 651762 | 1356 | 531 | 649 | 176 | 36.4   ( 35.9 , 36.9 ) | 48.6  ( 48.3 , 48.8 ) | 11.8   ( 10.6 , 13 ) | 8.4   ( 7.5 , 9.4 ) | 1.6  ( 0.9 , 2.2 ) | 1.7   ( 0.5 , 2.9 ) | 8.4   ( 7.5 , 9.4 ) | 1.6   ( 0.9 , 2.2 ) | 1.7   ( 0.5 , 2.9 ) |

| Uruguay | 24 | 144 | 58 | 0 | 30 | 28 | -   ( - , - ) | 49.6  ( 49.2 , 50 ) | 48.7   ( 48.3 , 49.1 ) | -   ( - , - ) | 183.9  ( 183 , 184.9 ) | 658.6   ( 658.1 , 659.2 ) | -   ( - , - ) | 0.1   ( -0.8 , 1.1 ) | 0.5   ( -0.1 , 1.2 ) |
| --- | --- | --- | --- | --- | --- | --- | --- | --- | --- | --- | --- | --- | --- | --- | --- |
| Uruguay | 25 | 187 | 68 | 5 | 42 | 21 | 9.1   ( 8.1 , 10.2 ) | 53.7   ( 52.7 , 54.7 ) | 33.5   ( 32 , 35 ) | 343.3   ( 342.7 , 343.9 ) | 185.2   ( 183.7 , 186.7 ) | 462   ( 461. , 462.5 ) | 0.6   ( -0.3 , 1.5 ) | 0.2   ( -1.6 , 1.9 ) | 0.4   ( -1 , 1.8 ) |
| Uruguay | 26 | 295 | 60 | 7 | 36 | 17 | 15.9   ( 14.8 , 16.9 ) | 59.5   ( 59.1 , 60 ) | 27   ( 26.6 , 27.5 ) | 441.8   ( 440.1 , 443.4 ) | 122.8   ( 121.9 , 123.7 ) | 272.9   ( 272.2 , 273.7 ) | 0.9   ( 0.1 , 1.7 ) | 0.2   ( -0.7 , 1 ) | 0.3   ( -0.8 , 1.4 ) |
| Uruguay | 27 | 316 | 57 | 13 | 31 | 13 | 22.3   ( 21.4 , 23.1 ) | 45.7  ( 44.4 , 47 ) | 20.8   ( 19.8 , 21.8 ) | 317.3   ( 316.2 , 318.4 ) | 96.6   ( 95 , 98.1 ) | 174.6   ( 173.7 , 175.4 ) | 1   ( 0.2 , 1.8 ) | 0.1   ( -1.5 , 1.8 ) | 0.2   ( -0.8 , 1.3 ) |
| Uruguay | 28 | 437 | 47 | 14 | 24 | 9 | 27.6   ( 26.9 , 28.4 ) | 49.0  ( 48.5 , 49.6 ) | 19.2   ( 18.5 , 19.9 ) | 278   ( 277.4 , 278.7 ) | 62.7   ( 61.7 , 63.6 ) | 98.9   ( 98.3 , 99.5 ) | 1   ( -0.1 , 2.2 ) | 0.1   ( -0.9 , 1.2 ) | 0.2   ( -0.5, 0.8 ) |
| Uruguay | 29 | 438 | 44 | 10 | 24 | 10 | 30.5   ( 29.6 , 31.5 ) | 53.3   ( 52.7 , 53.8 ) | 25.4   ( 24.6 , 26.3 ) | 309.2   ( 308.6 , 309.9 ) | 60.4   ( 59.5 , 61.3 ) | 172.2   ( 171.4 , 173.1 ) | 1.3   ( 0.6 , 1.9 ) | 0.1   ( -0.8 , 1 ) | 0.2   ( -0.8 , 1.3 ) |
| Uruguay | 30 | 666 | 50 | 15 | 30 | 5 | 30.4   ( 29.3 , 31.5 ) | 61.9   ( 61.1 , 62.6 ) | 16.8   ( 16.3 , 17.4 ) | 203.4   ( 201.8 , 204.9 ) | 56.8   ( 56.2 , 57.5 ) | 59.2   ( 58.5 , 59.9 ) | 1.5   ( -0.2 , 3.2 ) | 0.1   ( -0.5 , 0.8 ) | 0.1   ( -0.5 , 0.8 ) |
| Uruguay | 31 | 798 | 42 | 8 | 25 | 9 | 26.6   ( 25.3 , 27.8 ) | 55.6   ( 55 , 56.2 ) | 20.2   ( 19.3 , 21.1 ) | 136.6   ( 135 , 138.3 ) | 34.4  ( 33.4 , 35.4 ) | 77.9   ( 76.7 , 79.1 ) | 0.8   ( -0.5 , 2.2 ) | 0.1   ( -0.8 , 1 ) | 0.2   ( -0.8 , 1.1 ) |
| Uruguay | 32 | 1267 | 51 | 13 | 31 | 7 | 27.9   ( 26.9 , 29 ) | 60.4   ( 60 , 60.8 ) | 19   ( 18.3 , 19.6 ) | 108.6   ( 107.7 , 109.5 ) | 27.9   ( 27.3 , 28.6 ) | 78.9   ( 78 , 79.8 ) | 1.2   ( 0.1 , 2.2 ) | 0.1   ( -0.5 , 0.8 ) | 0.2   ( -0.5 , 0.9 ) |
| Uruguay | 33 | 1614 | 45 | 11 | 29 | 5 | 22 .0  ( 20.6 , 23.4 ) | 60.6   ( 60 , 61.2 ) | 17.4   ( 16.3 , 18.6 ) | 85.4   ( 84 , 86.8 ) | 19.3   ( 18.3 , 20.2 ) | 58.4   ( 57.4 , 59.4 ) | 0.9   ( -0.2 , 1.9 ) | 0.1   ( -0.8 , 1.1 ) | 0.2   ( -1 , 1.4 ) |
| Uruguay | 34 | 3032 | 51 | 19 | 24 | 8 | 33.3   ( 32.3 , 34.3 ) | 44.5   ( 43.7 , 45.2 ) | 23   ( 22.1 , 23.8 ) | 70.3   ( 69.2 , 71.4 ) | 8.7   ( 7.8 , 9.6 ) | 40.3   ( 39.3 , 41.3 ) | 1.5   ( 0.3 , 2.7 ) | 0.1   ( -0.7 , 1 ) | 0.2   ( -0.6 , 1 ) |
| Uruguay | 35 | 4869 | 42 | 5 | 36 | 1 | 36.5   ( 34.8 , 38.2 ) | 78.2   ( 77.4 , 79 ) | 14.3   ( - , - ) | 28.3   ( 26.6 , 30 ) | 7.9   ( 6.8 , 9.2 ) | 11.8   ( - , - ) | 1   ( -0.8 , 2.9 ) | 0.2   ( -1.1 , 1.4 ) | 0.1   ( - , - ) |
| Uruguay | 36 | 9848 | 69 | 15 | 48 | 6 | 21.8   ( 21.5, 22.2 ) | 69.8   ( 69.6 , 70 ) | 10.9   ( 10.1 , 11.7 ) | 19.4   ( 18.8 , 20 ) | 5.9   ( 5.6 , 6.4 ) | 6.3   ( 5.5 , 7.1 ) | 1.4   ( 1 , 1.8 ) | 0.2   ( -0.1 , 0.6 ) | 0.2   ( -0.7 , 1 ) |
| Uruguay | 37 | 24246 | 91 | 25 | 49 | 17 | 24.8   ( 23.8 , 25.7 ) | 49.2   ( 48.3 , 50.2 ) | 21.4   ( 20.5 , 22.3 ) | 14.3   ( 13.2 , 15.4 ) | 2.4  ( 1.3 , 3.5 ) | 4.6   ( 3.9 , 5.3 ) | 2.3   ( 1 , 3.5 ) | 0.2   ( -0.9 , 1.4 ) | 0.4   ( -0.3 , 1.1 ) |
| Uruguay | 38 | 56422 | 75 | 18 | 49 | 8 | 25.7   ( 24.7 , 26.8 ) | 62.6   ( 61.9 , 63.2 ) | 16.4   ( 15.9 , 16.9 ) | 9   ( 8.2 , 9.7 ) | 1.1   ( 0.3 , 1.9 ) | 1.1   ( 0.9 , 1.2 ) | 2.5   ( 1.7 , 3.2 ) | 0.3   ( -0.5 , 1.1 ) | 0.3   ( 0.1 , 0.5 ) |
| Uruguay | 39 | 84131 | 102 | 18 | 64 | 20 | 18.5   ( 18.1 , 18.8 ) | 64.1  ( 63.9 , 64.4 ) | 17.5   ( 16.3 , 18.7 ) | 6.9   ( 5.7 , 8 ) | 0.9   ( 0 , 1.8 ) | 0.9   ( -0.5 , 2.3 ) | 3.2   ( 2 , 4.3 ) | 0.5   ( -0.4 , 1.4 ) | 0.5   ( -0.9 , 1.9 ) |
| Uruguay | 40 | 60840 | 149 | 20 | 109 | 20 | 14.4   ( 13.5 , 15.4 ) | 70.3   ( 70 , 70.7 ) | 14.8   ( 14 , 15.5 ) | 8.3   ( 7.8 , 8.7 ) | 2.0   ( 0.9 , 3.2 ) | 1.5   ( 0 , 3.1 ) | 6   ( 5.5 , 6.4 ) | 1.7   ( 0.5 , 2.8 ) | 1.3   ( -0.3 , 2.9 ) |
| Uruguay | 41 | 12922 | 172 | 13 | 133 | 26 | 8.2   ( 6.2 , 10.3 ) | 75.1   ( 74.8 , 75.4 ) | 13.8   ( 12.9 , 14.7 ) | 14.1   ( 12.8 , 15.4 ) | 10.7   ( 9.3 , 12.2 ) | 9.8   ( 8.2 , 11.4 ) | 13.2   ( 11.9 , 14.5 ) | 10.5   ( 9 , 11.9 ) | 9.6   ( 8 , 11.2 ) |
| Uruguay | >=42 | 204 | 132 | 13 | 93 | 26 | 10.3   ( 7.9 , 12.6 ) | 70.2  ( 69.8 , 70.6 ) | 20.9   ( 20 , 21.9 ) | 214.3   ( 212.7 , 215.9 ) | 348.5  ( 347.8 , 349.2 ) | 601.6   ( 600.7 , 602.4 ) | 214.3   ( 212.7 , 215.9 ) | 348.5   ( 347.8 , 349.2 ) | 601.6   ( 600.7 , 602.4 ) |

# Table S6. Summary of metadata

| **Country** | **Units for recording** | **Data source for live and stillbirths** |  |  |  | **Reporting criteria for births** | | |
| --- | --- | --- | --- | --- | --- | --- | --- | --- |
|  | **Birthweight (e.g., gram/ oz’ lb)/ Gestational age (e.g., weeks, days)** | **And details of linkage where relevant** | **Livebirths: Exclusions criteria based on BW** | **Livebirths:**  **Exclusion criteria based on GA** | **Stillbirths: Exclusions criteria based on BW** | **Stillbirths:**  **Exclusion criteria based on GA according to legal requirement** | **Legal requirement for stillbirth reporting** | **Are births following induced Termination of Pregnancy included in the data source?** |
| Australia | Grams | Not applicable as livebirths, stillbirths and neonatal deaths are all included as part of the National Perinatal Data Collection | A small number of births <400 grams are included | A small number of births < 20 weeks are included |  | <20 weeks |  | Both livebirths and stillbirths may include termination of pregnancy after 20 weeks. |
|  | Completed weeks |  |  |  |  |  |  |  |
| Argentina | Grams |  | There are no birthweight limits if the baby was born alive | There are no gestational age limits if the baby was born alive |  |  |  | They are included among the stillbirths |
|  | Completed weeks |  |  |  |  |  |  |  |
|  | Completed weeks |  |  |  |  |  |  |  |
| Denmark | Grams | Information on livebirths and stillbirths were extracted from the Danish Medical Birth Registry. | None |  |  | <22 weeks^5^ |  | No |
|  | Completed weeks |  |  |  |  |  |  |  |
| England & Wales | Grams | Data linkage with birth notifications, and birth and stillbirth registrations | None |  |  | Stillbirths (>=24 weeks) |  | No |
|  | Weeks + days |  |  |  |  |  |  |  |
| Estonia | Grams | Estonian Medical Birth Register | None |  |  | <22 weeks. Abortions can be made up to 21 weeks and 6 days only in case of medical indication, all stillbirths (i.e. dead before expulsion or extraction ) are included from 22 weeks onwards  ^.^ |  | No |
|  | Days |  |  |  |  |  |  |  |
| Iran |  |  |  |  |  |  |  |  |
|  |  |  |  |  |  |  |  |  |
| Lebanon | Grams | None | None |  |  | <22 weeks |  | No |
|  | Weeks + days |  |  |  |  |  |  |  |
|  |  |  |  |  |  |  |  |  |
| Malaysia | Grams  Completed weeks | None | None |  |  | None |  | No |
| Mexico | Grams  Completed weeks | Livebirths and deaths records were linked using the variables sex, date of birth, place of residence and place of occurrence using CIDACS-RL software | None |  |  | None |  | No |
| The The Netherlands | Grams |  | Gestational age <22 weeks; if ga. missing, birthweight<500 g |  |  | Gestational age <22 weeks; if ga. missing, birthweight<500 g |  | Both livebirths and stillbirths up to 24 weeks gestation may include termination of pregnancy |
|  | Completed weeks |  |  |  |  |  |  |  |
| Qatar | Kilograms and grams |  | None |  |  | None |  | No |
|  | Weeks and days |  |  |  |  |  |  |  |
| Scotland | Grams | Live, stillbirth (>=24wks) and late fetal death (22/23 wks) records were derived from SMR02 (hospital IP/DC hospital return for obstetric data). Additional stillbirths were identified by matching the hospital record to the statutory stillbirth register using the Mother unique ID. | None |  |  | None | Stillbirths (>=24 weeks) must be registered with National Records of Scotland within 21 days of the delivery.  Late fetal deaths (22/23 weeks) are not registered. | Termination of pregnancy records are excluded. |
|  | Completed weeks |  |  |  |  |  |  |  |
| Sweden | Grams | national databases that are linked together using the person-unique national registration numbers of children | None |  |  | <23 weeks | In Sweden information on stillbirths is available from 28 weeks onward from 1997 to July 1, 2008, and thereafter from 22 gestational weeks | Included after 22 weeks |
|  | Days |  |  |  |  |  |  |  |
| Uruguay | Grams |  | None |  |  | None |  | No |
|  | Completed weeks |  |  |  |  |  |  |  |
| United States of America | Grams (and lb converted to grams) Completed weeks | Data on live births and fetal deaths are available from the NCHS – Vital Statistics online. It includes data from live birth certificates and fetal death certificates. | None |  |  | <20 weeks^5^ |  | The NCHS recommendation for fetal death definition is to exclude TOPs. Some states do exclude TOPs regardless of gestational age, while some states include TOPs |
